# Multi-habitat landscapes are more diverse and stable with improved function

**DOI:** 10.1038/s41586-024-07825-y

**Published:** 2024-08-21

**Authors:** Talya D. Hackett, Alix M. C. Sauve, Kate P. Maia, Daniel Montoya, Nancy Davies, Rose Archer, Simon G. Potts, Jason M. Tylianakis, Ian P. Vaughan, Jane Memmott

**Affiliations:** 1 School of Biological Sciences, University of Bristol, Bristol, UK; 2 Department of Biology, University of Oxford, Oxford, UK; 3 Department of Computer Science, University of Bristol, Bristol, UK; 4 University of Bordeaux, Integrative and Theoretical Ecology group, LabEx COTE, Pessac, France; 5 Institute of Biosciences Institute, University of São Paulo, São Paulo, Brazil; 6 Basque Centre for Climate Change (BC3), Parque Científico UPV-EHU, Leioa, Spain; 7 IKERBASQUE, Basque Foundation for Science, Bilbao, Spain; 8 Centre for Agri-Environmental Research, School of Agriculture, Policy and Development, University of Reading, Reading, UK; 9 Bioprotection Aotearoa and Centre for Integrative Ecology, School of Biological Sciences, University of Canterbury, Christchurch, New Zealand; 10 School of Biosciences, Cardiff University, Sir Martin Evans Building, Cardiff, UK

**Keywords:** Food webs, Ecological networks, Ecosystem services

## Abstract

Conservation, restoration and land management are increasingly implemented at landscape scales^1,2^. However, because species interaction data are typically habitat- and/or guild-specific, exactly how those interactions connect habitats and affect the stability and function of communities at landscape scales remains poorly understood. We combine multi-guild species interaction data (plant–pollinator and three plant–herbivore–parasitoid communities, collected from landscapes with one, two or three habitats), a field experiment and a modelling approach to show that multi-habitat landscapes support higher species and interaction evenness, more complementary species interactions and more consistent robustness to species loss. These emergent network properties drive improved pollination success in landscapes with more habitats and are not explained by simply summing component habitat webs. Linking landscape composition, through community structure, to ecosystem function, highlights mechanisms by which several contiguous habitats can support landscape-scale ecosystem services.

## Abstract (web summary)

Species interaction data, a field experiment and modelling of plant–insect communities show that landscapes with more habitat types support more even species, more complementary interactions, are more consistently robust to species loss, and confer greater pollination function.

## Main

Conservation policy and landscape management have moved from the historic protection of species and their habitats to ecosystem and landscape-level approaches^1,2^. Habitat heterogeneity^3,4^ and the number of habitats in a landscape^5,6^ contribute to species richness and ecosystem functioning, especially in agricultural landscapes^7,8^. At present, we lack a mechanistic understanding of how the number of habitats contributes to community structure and function. This understanding is key to the landscape-scale management of ecosystem services that depend on species interactions, such as pollination and pest control and to maintaining functioning ecosystems more generally. Ecological networks of species’ interactions provide a route to understanding functional responses to biodiversity changes^9,10^. Although communities host several guilds and transcend different habitats, network datasets encompassing both these characteristics remain scarce, meaning we might be missing important cross-habitat or guild cascades or functional effects. Researchers have recently started linking several networks, of one interaction type across habitats^11^, several interaction types across habitats^12^ or several interactions at replicated sites of similar habitat composition^8^. However, a lack of independently derived measures of function has prevented these network changes from being linked mechanistically to functional outcomes.

Both structure and function of local communities can be affected by organisms dispersing between habitats^13^. Immigrating individuals may have a similar role to local species (that is, redundancy) or fill an empty ecological niche (that is, complementarity). If immigrating and local species respond differently to disturbances, this can reduce functional variability by ensuring that overall functionality is maintained^14–17^. Community impacts of dispersal may differ across trophic groups^18^, making the combination of habitat and guild replication critical. Despite the importance to both pure and applied ecology, it remains unknown whether landscapes are simply the sum of their habitat parts, in terms of both interaction structure and community function, or whether there are emergent properties, such as increased stability or functioning, that cannot be explained by their component habitats alone.

**Fig. 1:**
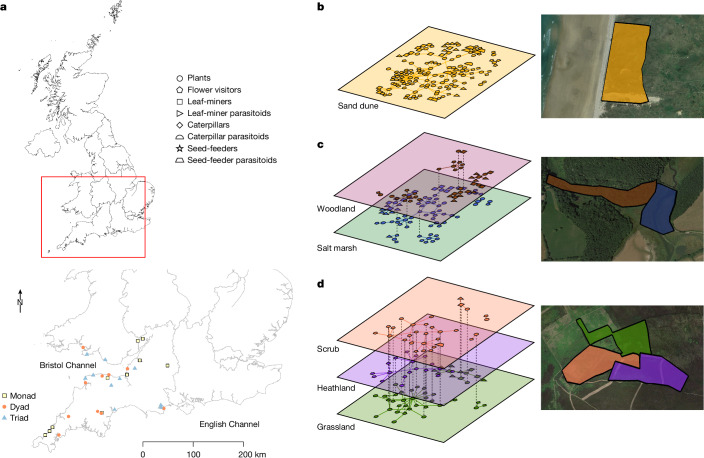
Map of sites and example networks. **a**, Map of sites for monads of one 9 ha habitat, dyads of two 4.5 ha habitats and triads of three 3 ha habitats in southwest United Kingdom. **b**–**d**, Visualization of the plant–insect interactions as multilayer networks and satellite images of Penhale Sands, a sand dune monad (**b**), Seet Bridge, a woodland and salt marsh dyad (**c**) and Hangman’s Hill, a scrub and heathland and grassland triad (**d**). Each layer corresponds to one habitat with nodes as species and shapes coding for the species type. Interspecific interactions in each habitat (layer) are represented with solid lines. Dashed lines connect nodes between layers representing the same species in different habitats. Map data sources: Office for National Statistics licensed under the Open Government Licence v.3.0; contains OS data © Crown copyright and database right 2021. Google Earth Pro Image © 2024 (CNES/Airbus (**b**) and Landsat/Copernicus (**c** and **d**)).

Here we evaluate how the number of habitats in a given area influences biodiversity, network structure, community stability and function across several interaction types (plant–pollinator and three types of plant–herbivore–parasitoid networks) in replicate landscapes. Using 30 independent field sites in southwest United Kingdom (Fig. 1a), we test how landscapes with more habitats affect the plant and insect communities in them: specifically, we quantify the effects on species’ abundances, species richness, evenness (both in terms of insect species and the degree to which interactions are uniformly distributed among species) and robustness to species loss. Using a manipulative field experiment, we then test whether the number of habitats affects the ecological function of insect pollination. Finally, we develop a modelling approach to investigate if landscape-scale networks have emergent properties which cannot be explained by their component habitat networks.

We standardized field site size at 9 ha and varied the number of constituent habitats from one to three while selecting sites to allow a balanced replication of multi-habitat landscape types: thus, ten sites contained a single habitat (9 ha ‘monads’), ten sites contained two habitats (‘dyads’ with two 4.5 ha habitats) and ten sites contained three habitats (‘triads’ with three 3 ha habitats). Each habitat was selected from a pool of six habitats: grassland, heathland, woodland, salt marsh, sand dune and scrub (Fig. 1b–d and Supplementary Table 3) to avoid a habitat identity effect confounding that of the number of habitats, while also avoiding the issue of triads always having the same composition whereas monads and dyads differ. Over 2 years, we collected data on 11,482 interactions among 154 plant species and 954 insect species (5,729 flower–visitor interactions, 2,345 plant–leaf miner interactions, 697 plant–caterpillar interactions, 1,240 plant–seed feeding interactions and 1,471 herbivore–parasitoid interactions; see Fig. 1b–d for example networks, whereby species are depicted as nodes connected by interactions as links).

## Community diversity and structure

**Fig. 2:**
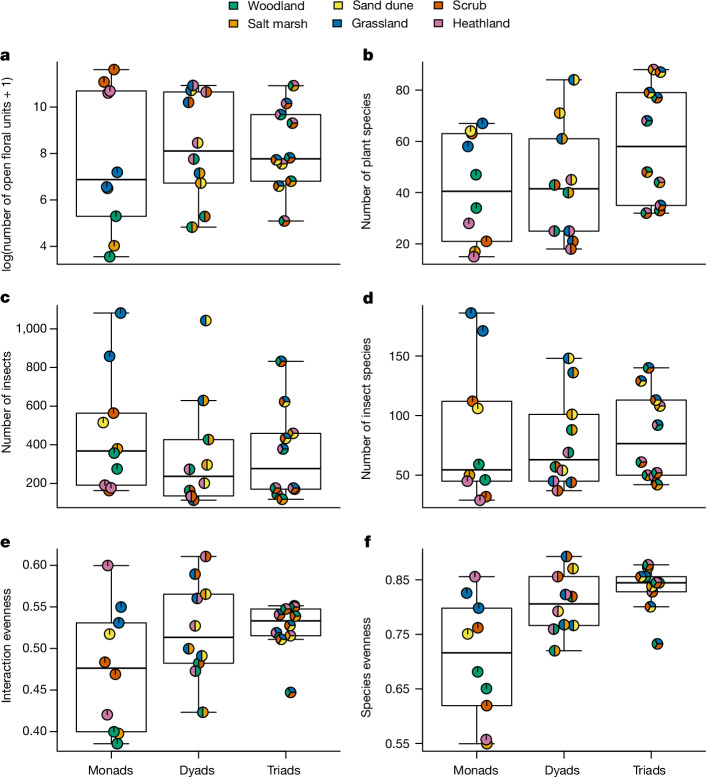
Community diversity and structure. **a**–**f**, Differences among monads, dyads and triads in terms of floral abundance (**a**), plant species richness (**b**), insect abundance (**c**), insect species richness (**d**), interaction evenness (**e**) and species evenness (**f**). Circles indicate each site and the habitat combination therein, with a random horizontal jitter to reduce overlap. Data are from 30 independent field sites, 558,386 open floral units (154 plant species) and 11,482 interactions (954 insect species). Boxes represent the 25% (Q1) and 75% (Q3) quartiles around the median line and whiskers are Q1 − 1.5× IQR to Q1 and Q3 to Q3 + 1.5× IQR. See Extended Data Fig. 1 and Supplementary Information section 6 for subwebs.

There was a significant difference in community composition and network structure among monads, dyads and triads. Thus, insect species richness and abundance, plant species richness, floral abundance, insect species evenness and interaction evenness in 9 ha landscapes were higher when there were more habitats (multiple analysis of variance (MANOVA), *F*_1,28_ = 5.366; *P* = 0.001; Fig. 2 and Supplementary Table 2). Specifically, pairwise MANOVAs demonstrated significant increases from monads to triads (*F*_1,18_ = 5.552; *P* = 0.005; Fig. 2). To better understand the specific aspects driving the MANOVA results, generalized linear models showed that more habitats in the landscape supported non-significant increases of plant species richness (mean ± s.d. for monads, 41.4 ± 20.77; dyads, 43.3 ± 22.5; triads, 59.1 ± 22.99; *F*_1,28_ = 3.244; *P* = 0.082; Fig. 2b) and interaction evenness (monads, 0.48 ± 0.07; dyads, 0.52 ± 0.06; triads, 0.52 ± 0.03; *F*_1,28_ = 3.767; *P* = 0.062; Fig. 2e) and a significant increase in insect species evenness (monads, 0.71 ± 0.11; dyads, 0.81 ± 0.06; triads, 0.83 ± 0.04; *F*_1,28_ = 14.92; *P* < 0.001; Fig. 2f); all other factors showed no significant difference when considered independently (floral abundance, insect species richness and insect abundance all *F*_1,28_ < 0.714; *P* > 0.405; Fig. 2a,c,d).

## Community robustness

**Fig. 3:**
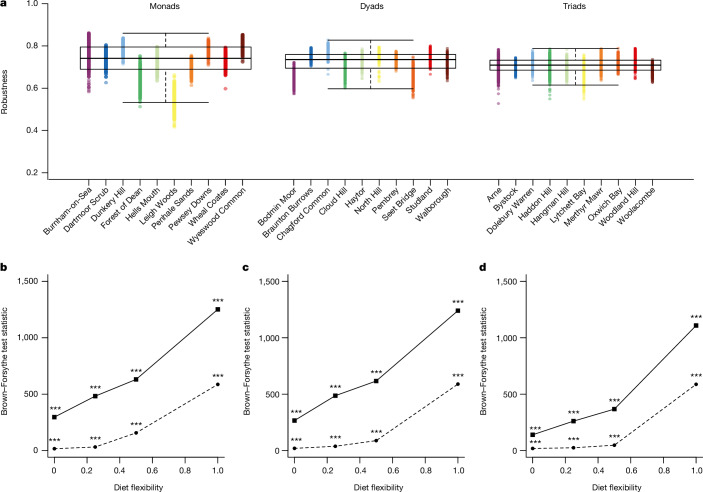
Community robustness. **a**, Robustness calculations for each site with 100% dietary flexibility for insects and 50% extinction threshold. Each point is a robustness calculation, colour-coded for each site within landscape type. Boxplots indicate the variation for all sites of a landscape type (monad, dyad or triad). **b**–**d**, Brown–Forsythe’s test statistic (two-sided), measuring the equality of group variance, for three extinction thresholds (25% (**b**); 50% (**c**); 75% (**d**)) and various levels of dietary flexibility. Dashed lines correspond to the random extinction scenario, stars code for the significance level of the test. For each landscape type (monad, dyad and triad), *n* = 5,000 (ten sites × 500 replicates). Boxes represent the 25% (Q1) and 75% (Q3) quartiles around the median line and whiskers are Q1 − 1.5× IQR to Q1 and Q3 to Q3 + 1.5× IQR. Asterisks indicate significance (***P < 0.001; all less than *P* = 7.46 × 10^−8^).

We measured stability as community robustness, a network-level calculation of the resistance of a community to species loss through secondary extinction, including all interaction types. Although stability has several components, to address the scope of this study, our data sampling design focused on spatial variability across a landscape and did not consider temporal dynamics. Mimicking bottom-up habitat degradation, we simulated removal of plant species across the landscape from least to most abundant at a given site because rare species are more likely to go extinct first^15^. Our robustness analysis (Methods) allows for rewiring, whereby species reallocate their interactions following the loss of a resource^16,19^ and accounts for shared species between interaction types resulting from ontogenetic diet shifts (for example, herbivorous caterpillar to pollinating butterfly); thus measuring the effects of species loss propagating through the multilayer network across different interaction types. There was no difference in mean robustness among monads, dyads and triads (*F*_2,27_ = 0.183; *P* = 0.83) but the variability of robustness decreased significantly as habitat number increased (interquartile range (IQR) of 0.105 in monads, 0.064 in dyads and 0.047 in triads; Brown–Forsythe’s test *F*_2,14997_ = 1,272.9; *P* < 0.001; Fig. 3). More flexible rewiring rules make the effect on robustness variability more apparent (Fig. 3 and Extended Data Fig. 2). This variability trend is stronger with sequential removal of rare-to-common species than with random removal of species, indicating that secondary extinction trajectories are not purely a result of species loss but rather associated with the distribution of rare species across habitats (Fig. 3 and Extended Data Fig. 3). Plant beta-diversity was similar in the monad, dyad and triad sites, indicating that the variability trend in robustness is unlikely to be a consequence of sampling bias because of specific habitat combinations overlapping among triads (Supplementary Information section 1).

## Community function

We conducted a manipulative field experiment to test how several habitats affected pollination function, defined here as fruit weight and quality. At the centre of each monad and triad, we placed 20 potted wild-type strawberry plants, *Fragaria vesca*, grown under standardized conditions, as they began flowering (Extended Data Fig. 4). Strawberries are an excellent bioassay plant as pollination quality can be easily quantified^8,20^ with several high-quality insect pollinator visits leading to larger and more symmetrical fruits (Class I fruits versus Class II fruits; Extended Data Fig. 5)^21^. Plants were left on site for 14 days to be pollinated and then kept in a pollinator-free greenhouse for 28 days. Ripe strawberries were weighed and graded as being Class I, if perfectly symmetrical, or Class II, if otherwise (Methods; Extended Data Fig. 5). Strawberries from triads were not heavier (*F*_1,16_ = 0.091; *P* = 0.122; Extended Data Fig. 6a), but they were 30.3% more frequently Class I than those at monad sites (*t*_11.3_ = 3.263; *P* = 0.007; Extended Data Fig. 6b), indicating that pollination was more effective at sites with more habitats. Triad sites were also more consistent in yielding high proportions of Class I fruits (Brown–Forsythe’s test *F*_1,11_ = 10.65; *P* = 0.007; Extended Data Fig. 6b).

**Extended Data Fig. 1:**
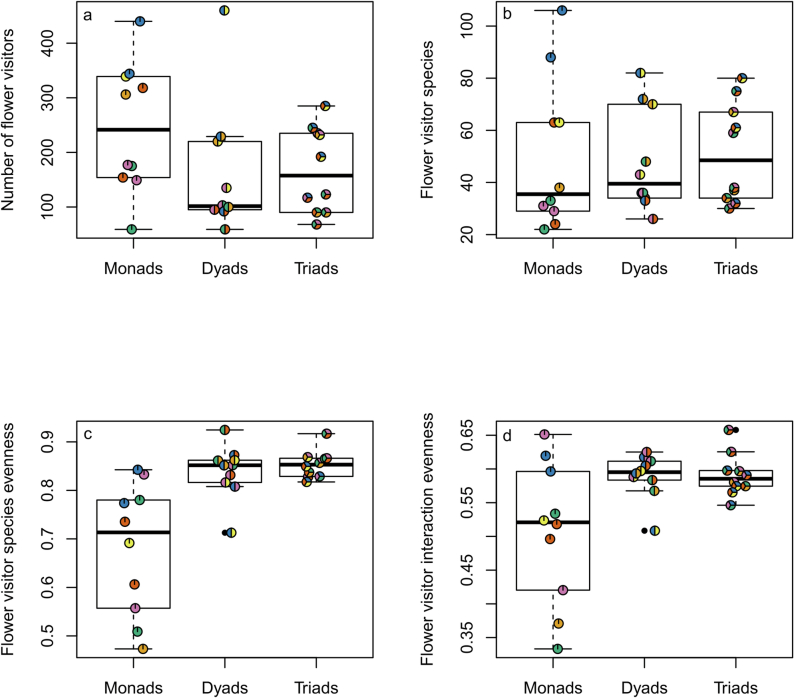
Flower visitors structural box plots. Differences among flower-visitors at monads, dyads and triads in terms of: A. abundance B. species richness, C. interaction evenness and D. species evenness. Circles indicate each site and the habitat combination therein with a random horizontal jitter to reduce overlap. Data are from 30 independent field sites and 5,729 flower-visitor interactions (526 species). Boxes represent the 25% (Q1) and 75% (Q3) quartiles around the median line, and whiskers are Q1 − 1.5xIQR to Q1 and Q3 to Q3 + 1.5xIQR.

Pollination is improved by several and varied pollinators^21,22^ but, although we found overall community differences between monads, dyads and triads (Fig. 2), pollinators were not more abundant or more species-rich at triads (Extended Data Fig. 1a,b). Therefore, to investigate if differences in pollination function could be explained by community interaction differences, we assessed the interaction complementarity of the pollinator community at each site by calculating the dietary dissimilarity of all recorded flower visitors, which has been shown experimentally to improve pollination success^23^. We then used a principal coordinate analysis and calculated the dispersion of the community to evaluate the breadth of dietary dissimilarity across all species at each site; thus sites with more dispersed diets will have higher interaction complementarity (Methods). Flower-visiting species at triads differed more in their diets (that is, had greater interaction complementarity) than those at monads (*t*_10_ = 8.42, *P* < 0.001; Extended Data Fig. 6c). Thus, although triads do not support more abundant or rich insect communities, they do host sets of pollinator species with more complementary diets; this observation is robust to the removal of rare or under-sampled species (Supplementary Information section 2). Flower–visitor interaction complementarity predicted the proportion of Class I strawberries (*F*_3,14_ = 3.475; *P* = 0.045; Extended Data Fig. 6d), which were higher with greater interaction complementarity but not fruit weight (*F*_3,14_ = 1.211; *P* = 0.342) which did not differ across monad and triad sites.

## Additive effects versus emergent properties

**Fig. 4:**
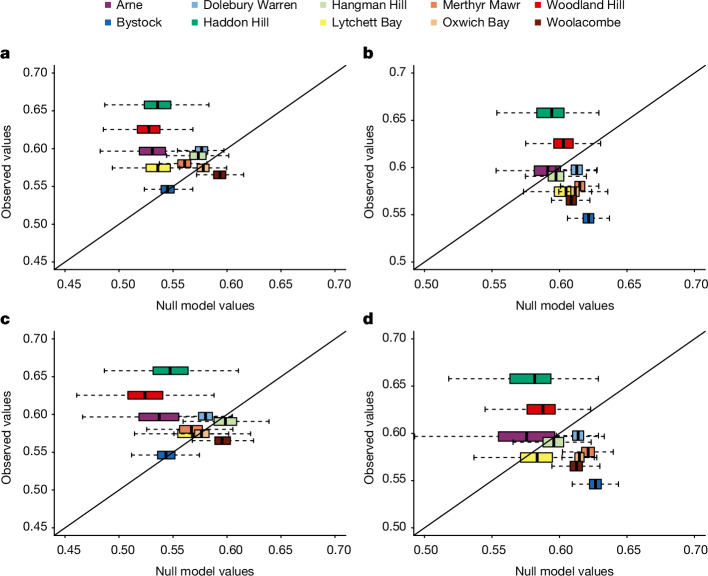
Additive effects versus emergent properties. Two variants of the same null model examining the potential for emergent properties at real triad habitat combinations. **a**–**d**, Controlling only for the number of interactions (**a**,**b**); also controlling for the number of plant species (**c**,**d**). Boxplots of the null model values of the interaction evenness (**a**,**c**) and functional dispersion (**b**,**d**) are plotted against the observed value at each site. Interaction complementarity is calculated as the functional dispersion of species at the real or null triads. If the boxplot overlaps the 1:1, then the observed value falls within the null hypothesis; if it is below, the observed value is less than under the null hypothesis and, if above, more than under the null hypothesis. For each boxplot (one per site), *n* = 1,000 replicates. Boxes represent the 25% (Q1) and 75% (Q3) quartiles around the median line and whiskers are Q1 − 1.5× IQR to Q1 and Q3 to Q3 + 1.5× IQR.

Lastly, we sought to understand whether the observed patterns in community structure and interaction complementarity of triads were due to an additive effect of several habitats or whether habitat combinations in the landscape present emergent properties. We used a null model and focused on the largest of the component networks: the plant–pollinator network. For each triad site, we created 1,000 null triads from independent observations at the component habitats (monads), while preserving the number of sampled interactions. We then calculated interaction evenness and complementarity for each null triad and compared it to the corresponding empirical triad (Methods). Interaction evenness was typically higher in empirical triads than null counterparts (7 of 10 triads; Fig. 4a) whereas interaction complementarity was lower (7 of 10 triads; Fig. 4b). These differences weakened but did not reduce to zero when controlling for the number of plant species at the site, indicating that the greater plant species richness of triads (Fig. 2b) only partially explains these emergent properties (Fig. 4c,d). It is possible that interaction evenness and complementarity could be due to differences in the plant phylogenetic diversity at a site if distinct phylogenetic groups are associated with different habitats and thus establish distinct sets of interactions. Increased interaction evenness and decreased complementarity at empirical versus null triads could, therefore, be associated with differences in plant phylogenetic diversity, which is indeed positively correlated with interaction complementarity (repeated measures correlation test *r*_9,969_ = 0.158, *P* < 0.001 and *r*_9,939_ = 0.158, *P* < 0.001 in Extended Data Fig. 7b,d, respectively) and negatively correlated with interaction evenness (*r*_9,969_ = −0.238, *P* < 0.001 and *r*_9,939_ = −0.201, *P* < 0.001 in Extended Data Fig. 7a,c, respectively). When constraining both models to include equal sampling completeness, the trend is weakened but broadly similar (Supplementary Information section 3 and Extended Data Fig. 8).

Landscape-scale effects are various and likely to contain trade-offs. Our results show that landscapes comprising several habitats support higher species and interaction evenness, more functionally diverse communities, with more consistent stability and greater pollination function, probably due to increased environmental heterogeneity at the landscape scale. Indeed, at the habitat-scale, a heterogeneous habitat structure allows for more niches and therefore higher biodiversity^3,7,24^. Our conclusions are unlikely to be confounded by surrounding patch size (Supplementary Information section 4) or sampling completeness differences (Supplementary Information section 5). For practicality, many management plans are habitat specific and focus on protecting habitat patches that are large and connected to similar ones (for example, many of the Living Landscapes, United Kingdom^2^; and Natura 2000 networks, European Union; along with prairie restoration projects, USA^25^). If multi-habitat landscapes are supporting communities with improved structure and functionality and more consistent robustness to species loss, as shown here, then maintaining diverse connected natural habitats across the wider landscape is likely also to be important. This is key to species conservation, as some species may depend on several habitats for different life stages (for example, different habitat requirements for larval herbivory and adult floral resources), ecological needs (for example, nesting in one habitat but foraging in another) or maximizing of resource availability across seasons (for example, the phenology of flowering plants varies among habitats).

Landscape simplification and habitat loss are significant stressors on biodiversity, community structure and ecosystem function. We found that more natural habitats provide a greater consistency in robustness. The higher variability in single-habitat landscapes means that extremes of robustness are more common, putting communities at individual sites more frequently at risk of cascading effects of species loss. At the landscape scale, the benefits of multi-habitat configurations therefore allow for a buffering effect, even if there is no average loss of robustness in landscapes with fewer habitats; more robust habitats might compensate for lower robustness in contiguous habitats resulting in a landscape-level decrease in robustness variability. To explore the mechanistic path underlying this trend (for example, with path analysis) would require several replications for each habitat combination; however, our results point to potential effects of landscape heterogeneity on community stability being mediated through interaction evenness^26^ (Fig. 2e) and tuned by plant phylogenetic diversity^27^ (Extended Data Fig. 7a,c). Our model indicates that this relationship between several habitats and robustness variability is more pronounced when species have greater flexibility to switch resources. This rewiring flexibility might be an important community response to stressors such as climate change and biodiversity loss^28^, suggesting that multi-habitat landscape configurations could provide even greater protection against environmental change.

Robustness to species loss and the rewiring of interactions are both related to interaction generalism. A greater proportion of generalists in the empirical triads could explain lower interaction complementarity compared to the null triads; yet, empirically, triad interaction complementarity was still higher than in empirical monads. Our field experiment indicates that several habitats supported better pollination with better-quality fruit set, which is explained not by more flower-visiting species or increased visits but rather by this higher interaction complementarity of pollinators at triads. Interaction complementarity, which could reduce the effect of conspecific pollen deposition^29^, positively correlates with the phylogenetic diversity of the plants supporting the food webs, suggesting that multi-habitat landscapes might increase complementarity through an increase in plant phylogenetic (and presumably functional) diversity.

Several habitat landscapes may therefore support both more interaction complementarity (for successful plant reproduction^30^) than single-habitat landscapes and greater redundancy through generalist species (which is important for robustness^31^) than expected by compiling the interactions from several independent habitats. Collectively, this link from landscape composition, through the plant–insect community structure, to ecosystem function provides a mechanism through which several habitats across the landscape can support stability and better ecosystem services.

## Methods

### Field sites

We sampled 30 sites in southwest England and southern Wales, each containing one, two or three of six habitat types: woodland, heathland, grassland, salt marsh, sand dunes and scrub. We sampled at ten single-habitat sites, monads, ten two-habitat sites, dyads and ten three-habitat sites, triads. The site size sampled remained constant, thus monads were a single habitat 9 ha in size, dyads consisted of two adjacent 4.5 ha habitats and triads consisted of three adjacent 3 ha habitats. A 9 ha field site size was selected to capture the diversity of different taxa across monads, dyads and triads, while also allowing effective sampling of all taxa at each site (plants, herbivores, pollinators and parasitoids). All sites were surrounded by the same habitats as those in the field site or water, urban environment or farmland habitats and each field site was visited once in May–September 2014 and three times in April–September 2015. In 2015, all sites were visited before any site was repeated. In each sampling round, sites were visited in ten three-site cycles, each comprising one monad, dyad and triad. The order of visited sites was randomized within both cycle and round.

Potential sites were initially selected with Arc GIS v.10.1 using the 2007 Land Cover Map^32^. Three GIS models selected sites that were (1) single-habitat 9 ha sites with a 500 m buffer that did not include any other habitat of interest (for example, urban, farmland or water were allowed in the buffer); (2) two 4.5 ha contiguous habitats with a 500 m buffer that did not include any other habitat of interest; and (3) three 3 ha contiguous habitats with a 500 m buffer that did not include any other habitat of interest. This created a long list of potential sites that were then verified and narrowed down using satellite images (Google Earth 2013) and finally ground-truthed to confirm habitat types, make final selections and outline appropriate habitat plots. The final site list was based on ease of access, travel time, the need to avoid geographic clustering of any site (Fig. 1a) or habitat type, along with our ability to secure permission to sample. We selected habitat combinations such that habitats were represented equally across monads, dyads and triads, while accounting for the restrictions of what was available in the southwest United Kingdom. Because some habitat combinations did not exist in accordance with our selection criteria and others consistently occur together (for example, sand dunes and salt marshes often border grasslands), across monads, dyads and triads, we sampled fewer sand dunes and salt marshes and more grassland and scrub. For a full list of sites see Supplementary Table 3.

### Data collection

At each site, on each visit, we sampled along six 35 m transects arranged as follows: six transects in the one monad habitat, three in each dyad habitat and two in each triad habitat. The transect start location and direction were randomly selected before arrival on site and changed on each of the four visits. Thus, in total we sampled along 24 transects at each site.

We designed our data collection under the assumption that sampling intensity is the main driver of species–area relationships, whereas the influence of patch size on per-unit-area (alpha) diversity is weak or absent^33,34^. Thus, larger patches typically have more species in total because they contain a variety of microhabitats. Repeated sampling across these larger patches would capture more microhabitats and therefore show high between-sample (beta) diversity^34^. Therefore, to control for these area effects on richness, the sampling effort was consistent at the site level^33,34^ and thus we standardized the number of samples per site to avoid a patch size bias. Collection protocol closely followed ref. ^12^ and is described below.

#### Plant sampling

On each visit, a 0.5 × 0.5 m^2^ gridded quadrat was placed on alternating sides of the transect every 10 m, resulting in four quadrats per transect. All plants were identified and given a vegetation abundance score (as in refs. ^12,35^). Category 1 species were rare, only present once to a few times (vegetation occupied 1–2% of the quadrat area), category 2 were present in high enough numbers to be seen easily (occupied less than 10% of the quadrat area), category 3 could be seen throughout the quadrat (less than 50% of the area) and category 4 were dominated by the given species (more than 50% of the area). Tree vegetation to a height of 2 m and grasses, the latter collectively pooled, were all classified on this 1–4 scale. All other flowering plant species were identified^36^ and floral abundance was further classified with buds, open, wilted and seed-set floral units counted. We calculated these per floral unit for flowers arranged in umbels, heads, capitula and spikes. Vegetation cover for flowering species was determined by the number of times a plant touched one of the 36 cross points formed by the intersecting grids on the quadrat. Any plant species that did not fall within a quadrat but which occurred within 30 m of the transect, were recorded but not included in quantitative analysis.

#### Plant–flower visitor network

On each visit, between 09:00 and 17:30 in dry, warm (minimum 15 °C) conditions with little to no wind, flower visitors were sampled by haphazardly walking for 20 min, no more than 30 m from the transect. All insects found on a flower head were collected using a hand net. Visited flowers were identified to species in the field and flower visitors were identified to species by taxonomists (see ‘Acknowledgements’).

#### Plant–herbivore–parasitoid networks

On each visit, we collected leaf miners and caterpillars from 1 m^2^ quadrats every 10 m on either side of the transect by visual searching of leaves to a height of 2 m. They were collected and stored individually and returned to the laboratory for rearing.

Leaf miners were initially identified from the leaf mine pattern^37,38^ and caterpillar species were identified at larval stage^39^. Individual larvae from both groups were reared in separate pots and checked every 2–3 days for emergence. Emerged adults, either parasitoid or herbivore, were identified by taxonomists (see Acknowledgements). We used adult identification of surviving individuals to confirm larval identifications, where possible, to ensure accurate identification for herbivores that were either killed by a parasitoid or died during rearing.

Seed herbivores and their parasitoids were collected in seeds in the first and fourth sampling rounds (that is, once in September 2014 and once in August–September 2015). Along each transect, we collected up to 50 seeds from plants expected to host seed feeders^12,40^. Seeds were collected from haphazardly sampled plants within 10 m of the transect and, where possible, from different plants, equally spaced along the transect. Each sample of up to 50 seeds was stored collectively in the same pot and checked weekly until adult herbivores and parasitoids emerged (up to 8 months). Each emerged insect, seed feeder or parasitoid, was collected, stored individually and identified by taxonomists. Insects were successfully reared from 23 plant species (*Anthyllis vulneraria, Aster tripolium, Centaurea nigra, Cirsium arvense, Cirsium eriophorum, Cirsium palustre, Cirsium vulgare, Crataegus monogyna, Lathyrus pratensis, Lotus corniculatus, Ornithopus perpusillus, Rhinanthus minor, Rosa rugose, Rubus fruticosus* agg., *Senecio jacobaea, Succisa pratensis, Trifolium arvense, Trifolium pratense, Trifolium repens, Ulex europaeus, Ulex galii, Viccia sativa* and *Vicia cracca*).

#### Pollination experiment

Between 4 and 14 July 2015, we placed 20 wild strawberry plants (*F. vesca*) in four 15 l buckets in the centre of each monad and triad; dyads were excluded to allow all plants to be placed and retrieved in the flowering time. Each bucket was surrounded by chicken wire to discourage disturbance by wildlife and livestock (Extended Data Fig. 4). Plants were at the point of flowering when put in the field, then left for 14 days to allow for natural pollination and then retrieved. It was not possible to collect data on strawberry visitation as the plants could only be left in position for a short period (as seed set occurs relatively quickly) and it was not feasible to simultaneously sample 20 geographically distant field sites (Fig. 1) in a meaningful fashion to record pollinator visitation during this period. *F. vesca* was selected as it grows naturally in the region, is visited by a wide range of pollinators^8^ and, although partly wind pollinated, insects are crucial for its successful, uniform pollination^21^, leading to higher seed set and more symmetrical fruits. Moreover, in commercial varieties, better pollination is linked with increased shelf life and market value^13^. At the end of the field experiment, we removed all new flower buds and stored the plants in an insect-free greenhouse, watering daily. Strawberries were picked when ripe, weighed and graded according to commercial symmetry ratings^41^: fruit containing only mild defects in shape (Class I) and those with more severe defects (Class II) (see Extended Data Fig. 5 for example pictures); fruit symmetry significantly affects the market value of commercial strawberries, hence the existence of a grading system. Fruit classes were assigned blindly by an assessor with no knowledge of the field sites, to avoid assessment bias. We stopped fruit collection on greenhouse-stored plants after 28 days.

### Data analysis

Data across all visits were summed to create one network per site with edges weighted by interaction frequency. All analyses were performed and graphs created in the R statistical environment (v.3.6.0)^42^. All data and code are available at Zenodo (10.5281/zenodo.11184586)^43^.

#### Community structure

We used a MANOVA to test for overall differences in community and network structure among monads, dyads and triads, based on plant species richness, floral abundance, insect species richness, insect abundance, Pielou’s species evenness and interaction evenness, which are expected to change according to land use^44^. Species evenness was calculated using vegan^45^ and interaction evenness using bipartite^46^. To determine the factors contributing to the MANOVA results, we performed pairwise MANOVAs between landscape types and general linear models for each of the six structural aspects (response variables). All residuals were normally distributed and homoscedastic, except for floral abundance, which required a log-transformation: log(*x* + 1).

#### Community robustness

To determine the response of ecological communities to species extinction, we evaluated the robustness of plant–insect networks to extinction of plant species from the least to the most common (as in ref. ^8^). We evaluated how common a plant species is by its average proportion in the landscape; the commonness *C*_*is*_ of plant species *i* in site *s* calculated as:

$${C}_{{is}}=\frac{1}{{H}_{s}}\mathop{\sum }\limits_{j=1}^{{H}_{s}}\frac{{a}_{{ij}}}{{A}_{j}}$$

where *H*_*s*_ is the number of habitats in site *s*, *a*_*ij*_/*A*_*j*_ is the proportion of plant species *i* in habitat *j*, defining *A*_*j*_ as the total abundance of plant species in habitat *j* and *a*_*ij*_ being the abundance of plant species *i* in habitat *j*. To calculate this metric for a given plant species, we used its average number of quadrat cross points in each habitat for a given site as a proxy of its local relative abundance.

**Extended Data Fig. 2:**
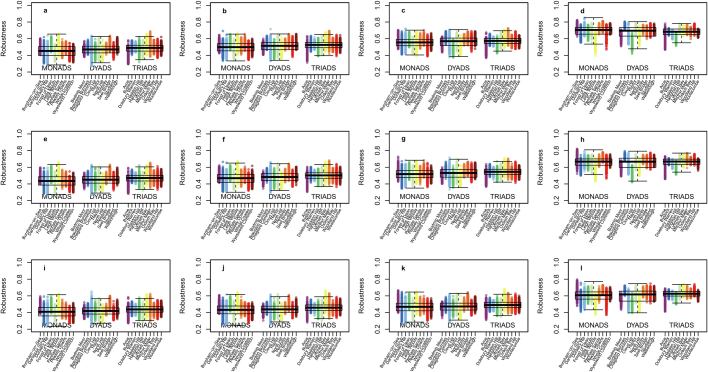
Least-to-most abundant scenario. Robustness calculations for each site under multiple extinction scenarios and with different levels of dietary flexibility. Dietary flexibility for insects was set at 0% (A, E, I), 25% (B, F, J), 75% (C, G, K) and 100% (D, H, L) dietary flexibility. Species’ sensitivity to interaction loss was set at: 25% (A-D), 50% (E-H) and 75% (I-L) of interactions. Plant species were removed from least to most abundant. For each landscape type (monad, dyad and triad), n = 5,000 (10 sites x 500 replicates). Boxes represent the 25% (Q1) and 75% (Q3) quartiles around the median line, and whiskers are Q1 − 1.5xIQR to Q1 and Q3 to Q3 + 1.5xIQR.

**Extended Data Fig. 3:**
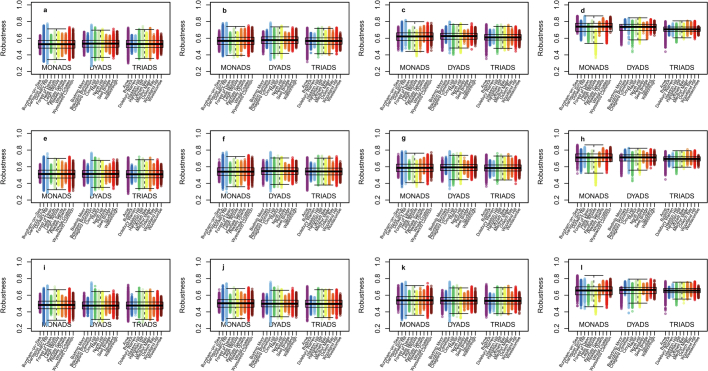
Random removal scenario. Robustness calculations for each site under multiple extinction scenarios and with different levels of dietary flexibility. Dietary flexibility for insects was set at 0% (A, E, I), 25% (B, F, J), 75% (C, G, K) and 100% (D, H, L) dietary flexibility. Species’ sensitivity to interaction loss was set at: 25% (A-D), 50% (E-H) and 75% (I-L) of interactions. Plant species were removed randomly. For each landscape type (monad, dyad and triad), n = 5,000 (10 sites x 500 replicates). Boxes represent the 25% (Q1) and 75% (Q3) quartiles around the median line, and whiskers are Q1 − 1.5xIQR to Q1 and Q3 to Q3 + 1.5xIQR.

**Extended Data Fig. 4:**
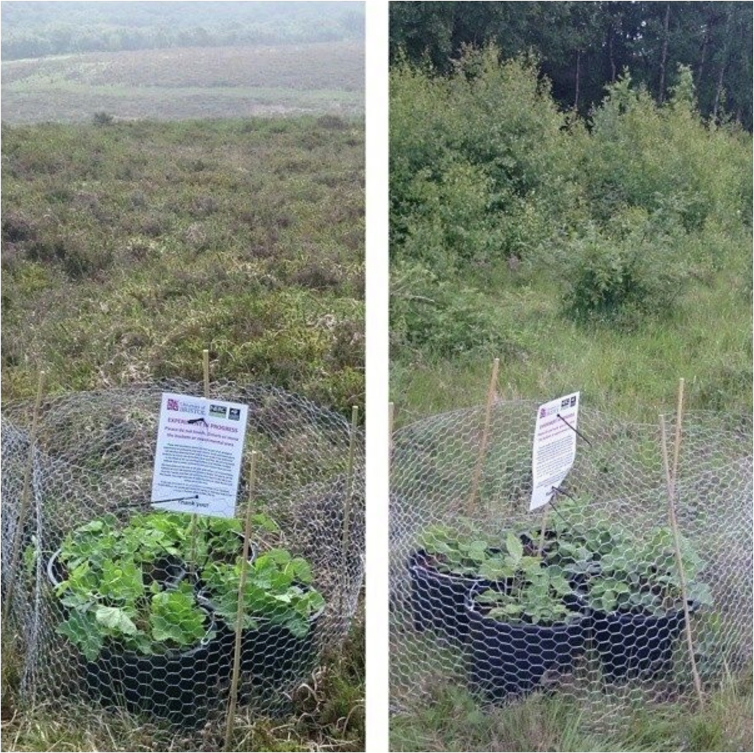
Photograph of pollination strawberry bioassay experiment. Pollination experiment set-up at a monad (left) and triad (right). Five wild-type strawberry (*Fragaria vesca)* plants were placed in each of the four buckets (total 20 plants) and surrounded with chicken wire to prevent grazing. Plants in flower bud were placed at habitat boundaries, where relevant, and as close to the centre of the plot as possible, left for two weeks and then retrieved and kept in a pollinator-free greenhouse.

**Extended Data Fig. 5:**
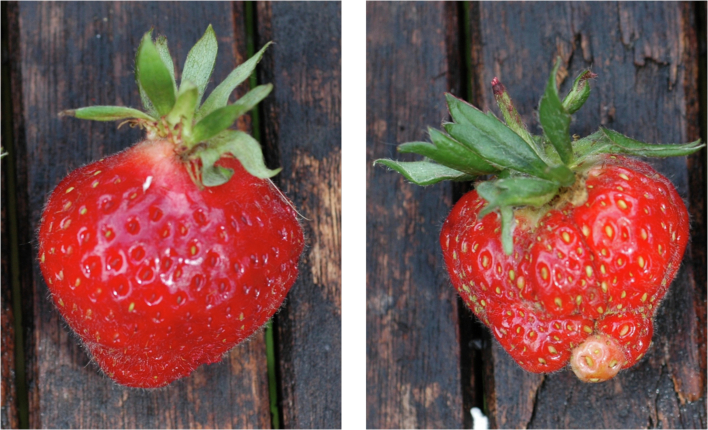
Example of a Class I and Class II strawberry. Perfect pollination allows all achenes to be pollinated evenly and fully and the surrounding tissue will swell to form fleshy fruit. This results in a symmetrical, Class I fruit (left). Imperfect pollination leads to an asymmetric, Class II (right), fruit as flesh only forms around fully pollinated achenes. In the same mechanism, better pollination also leads to larger fruit. While the Class I and II terminology was developed for commercial strawberries, it can be applied to wild-type strawberries too.

**Extended Data Fig. 6:**
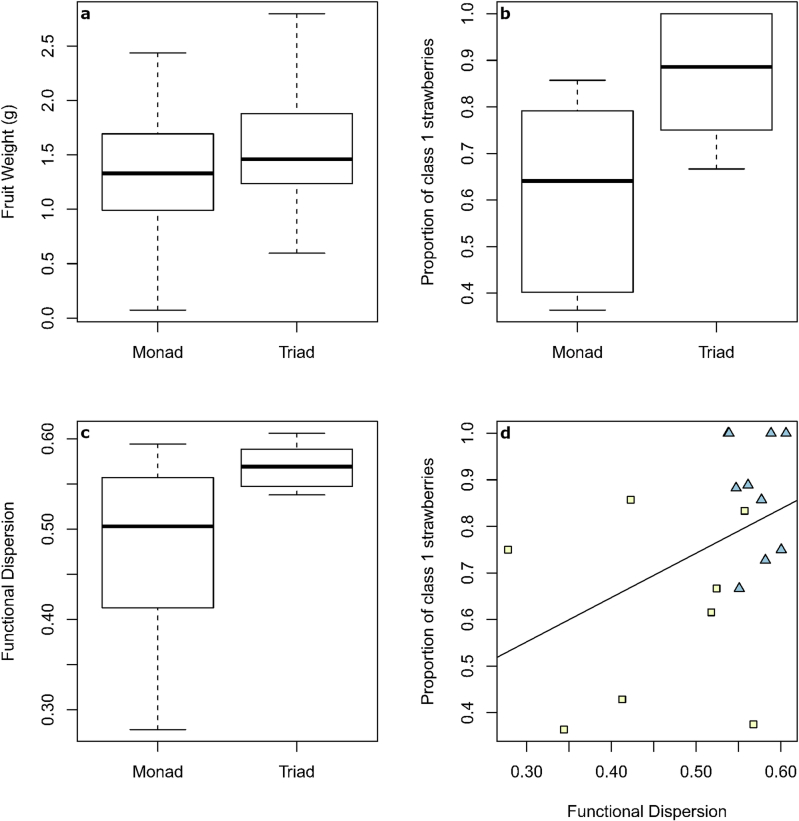
Strawberry fruit weight and quality and interaction complementarity. Difference in (A) Fruit weight, (B) Fruit quality and (C) Interaction complementarity at Monad and Triad sites. (D) The relationship between Interaction complementarity and the proportion of class 1 strawberries. Yellow squares are Monad sites and blue triangles are triads. Interaction complementarity is calculated as the functional dispersion of the species at each site and measured as the sum of the distances of all species in that site to the community centroid. Twenty strawberry plants were placed at 20 independent field sites, yielding a total of 144 strawberries. Boxes represent the 25% (Q1) and 75% (Q3) quartiles around the median line, and whiskers are Q1 − 1.5xIQR to Q1 and Q3 to Q3 + 1.5xIQR.

**Extended Data Fig. 7:**
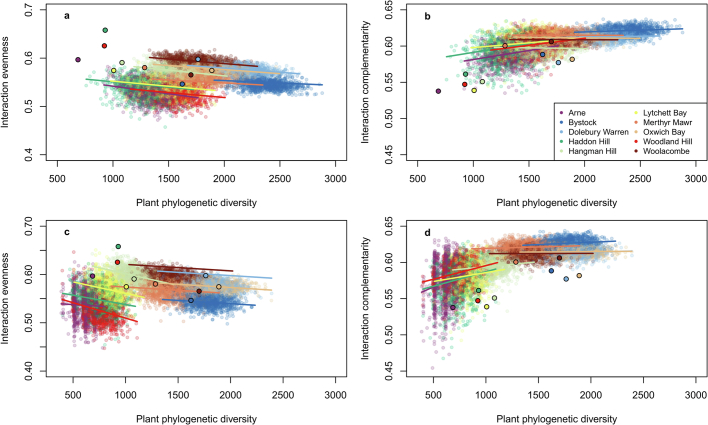
Null model 1&2: Effect of phylogenetic diversity. Interaction evenness (A&C) and interaction complementarity (B&D) as a function of plant phylogenetic diversity in random triads (coloured points) and observed triads (circles with coloured fill). Interaction complementarity is calculated as the functional dispersion of species at the real or null triads. Two variants of the same null model are displayed here, one controlling only for the number of interactions (A&B), and the other (C&D) also controlling also for the number of plant species.

We modelled a flexible behavioural response of upper trophic levels to their host plant’s extinction. Specifically, the extinction of a plant species can generate cascading loss of species which then allows for rewiring of the network. Herein, we assume that insects are able to reallocate part of their diet on similar resources/hosts, which we determined by identifying the taxa on which species with a similar niche (that is, sharing part of their diet with the focal insect species) feed^16^. Following ref. ^19^, we allowed species to reallocate lost interactions to alternative resources/hosts following a primary extinction and probability to interact with a given alternative resource/host was proportional to its abundance (approximated with interaction frequencies). We explored a range of species flexibility, from 0% flexibility (no rewiring allowed) to 100% flexibility (full reallocation of all lost interactions), including intermediate flexibility levels: 25% and 50%. We also explored the range of species’ sensitivity to interaction loss, expressed as a percentage of observed feeding interaction events below which a species is considered extinct: 25%, 50% and 75% of lost interactions (all cases are shown in Extended Data Figs. 2 and 3 and robustness to extinctions with full reallocation and 50% sensitivity level is shown in Fig. 3). We extend the approach of ref. ^19^ to multipartite networks with species interacting at different life stages, by assuming that if one life stage of one species goes extinct (for example, caterpillars), so do the others (for example, the corresponding adult butterfly in the flower–visitor network). Thus, species loss can propagate between two types of networks as a result of species being pollinators, herbivores or even parasitoids during different life stages or ecological requirements. Our approach allows rewiring alternatives and species extinction to be evaluated, respectively, for each interaction type and, for each life stage, when applicable.

We repeated this simulation 100 times, both under this scenario and under random removal. Whereas the former tests the response to plant species loss, the latter provides a control scenario accounting for the contribution of basic properties of the networks (size, number of links and so on) to community robustness.

#### Pollination experiment

The effect of habitat numbers (one or three) on fruit weight and the proportion of Class 1 strawberries (a measure of fruit quality that is determined by pollination success) was assessed using a mixed effect model with site as a random effect and the landscape type (monad or triad) as a fixed effect, using the package lme4 (ref. ^47^).

#### Interaction complementarity

Our field experiment supported the idea that pollination function is higher in sites with three habitats than in sites with a single habitat, even if pollinator richness and abundance were similar in sites with different numbers of habitats. Therefore, we asked whether the pollinator communities of sites with more habitats use floral resources in more complementary ways, compared with single-habitat sites, given that there is evidence that interaction complementarity can be associated with increased function^23^. Our measure of interaction complementarity was adapted from functional diversity analysis methods (for example, refs. ^48,49^) which measures, for instance, the breadth in species functional traits in ecological communities. Here we measure the breadth of pollinators’ use of flower resources; under the assumption that pollinator species with more dissimilar patterns of resource use complement the resource use of other pollinators, thereby increasing pollination function of the community^22^.

We started by computing the dissimilarity in resource use of pollinators from the plant–pollinator network data. To: (1) have a more complete picture of how complementary pollinator species interactions were in our communities and (2) create a multidimensional functional space which was comparable across sites, we computed a Bray–Curtis dissimilarity matrix from the complete plant–pollinator interaction network, that is all 30 sites pooled together in one interaction network. To control for a potential sampling completeness bias across pollinator species, we normalized pollinator interaction weights, so that interaction weights for each pollinator species summed to 1. We then performed a principal coordinate analysis (PCoA), to place pairwise dissimilarities into a multidimensional space, in which each species is represented by one point and Euclidean distances between species are proportional to their dissimilarities in resource use (Supplementary Fig. 1). We assessed the quality of the functional space using the mean squared deviation index^50^ and with no obvious break point, selected the space with ten dimensions. Using the FD package^51^, we then calculated the functional dispersion of each monad and triad site, measured as the sum of the distances of all species in that site to the community centroid. We took this to represent the overall pollinator interaction complementarity at the site level.

Data were normally distributed and had unequal variance, thus we used a Welch’s two sample *t*-test to determine the difference between the interaction complementarity at monads and triads. Using a linear model, we then tested whether interaction complementarity at each site predicted fruit weight and the proportion of Class 1 strawberries.

#### Null model tests for additive effects versus emergent properties of several habitats

Using a null model approach^27,52^, we tested whether the network properties we measured on the sites with several habitats are different from those expected if landscape-scale food webs are simply the sum of their habitats (the null hypothesis). To do so, we first created null triads, that is landscape-scale networks constructed from data collected at monad sites randomly assembled to represent the empirical triad landscapes. Then, we quantified interaction evenness and interaction complementarity for null triads and compared these to empirical triads.

Similar to other measures of biodiversity, network properties are expected to be affected by the size of the sampling area^53^ as in Supplementary Fig. 2 for three hypothetical habitats: *H*_1_, *H*_2_ and *H*_3_. If landscape-scale food webs are simply the sum of their habitats, then the null hypothesis should translate as follows in terms of species richness and number of interspecific interactions: a triad made of {*H*_1_, *H*_2_, *H*_3_} (each of area *Δ*/3, *Δ* being the area of the sampled site) should have fewer or an equal number of links ${L}_{\left\{{H}_{1},{H}_{2},{H}_{3}\right\},\left\{\varDelta /3,\varDelta /3,\varDelta /3\right\}}$ than the sum of the links in each habitat of size *Δ*/3 (that is, ${L}_{{H}_{1},\varDelta /3}+{L}_{{H}_{2},\varDelta /3}+{L}_{{H}_{3},\varDelta /3}$). Indeed, a lower number of links would occur if interactions are shared across several habitats. The same rationale applies to the number of species *S*:

$${S}_{\{{H}_{1},{H}_{2},{H}_{3}\},\{\varDelta /3,\varDelta /3,\varDelta /3\}}\le {S}_{{H}_{1},\varDelta /3}+{S}_{{H}_{2},\varDelta /3}+{S}_{{H}_{3},\varDelta /3}$$

We created two nested null models that generate random plant–insect interaction networks to test emergent properties within triads.

*Null model no. 1*. For an observed triad *T*_*k*_ made of {*H*_1_*, H*_2_*, H*_3_}, we created three random networks for each habitat *H*_*i*_ by subsampling the observed monad networks in the corresponding habitats. Thus, a null triad of woodland, heathland and grassland would be created by subsampling the equivalent number of interactions from woodland, heathland and grassland monads. A random network for the habitat *H*_*i*_ in the triad *T*_*k*_ was generated by subsampling ${{N}}_{{{T}}_{{k}},{{H}}_{{i}}}$ (the number of interaction events to consider for the {triad *T*_*k*_, habitat *H*_*i*_}-tuple) interactions among those observed in the monad with habitat type *H*_*i*_ (hereafter, monad ${{M}}_{{{H}}_{{i}}}$). The probability of sampling one interaction was assumed to be proportional to the number of times it had been observed in the monad ${{M}}_{{{H}}_{{i}}}$. For an interaction between species *b* and species *a* in monad ${{M}}_{{{H}}_{{i}}}$, this probability is:

$$P((a,b),{M}_{{H}_{i}})={N}_{ab,{M}_{{H}_{i}}}/{N}_{{M}_{{H}_{i}}}$$

where ${{N}}_{{ab},{{M}}_{{{H}}_{{i}}}}$ is the number of individuals of species *b* recorded interacting with species *a* in monad ${{M}}_{{{H}}_{{i}}}$ and ${{N}}_{{{M}}_{{{H}}_{{i}}}}$ is the total number of insect individuals seen interacting within monad ${{M}}_{{{H}}_{{i}}}$.

*Null model no. 2*. In this null model, we also controlled the diversity of the plant community to test whether it contributes to the differences between the observed triads and their random counterparts generated with null model no. 1. To this end, we follow the same steps as for the null model no. 1 but sampled interactions within a subset of monad ${{M}}_{{{H}}_{{i}}}$ involving the same number of plant species as observed interacting in the habitat *H*_*i*_ of the triad *T*_*k*_.

#### Evaluating emergent properties

**Extended Data Fig. 8:**
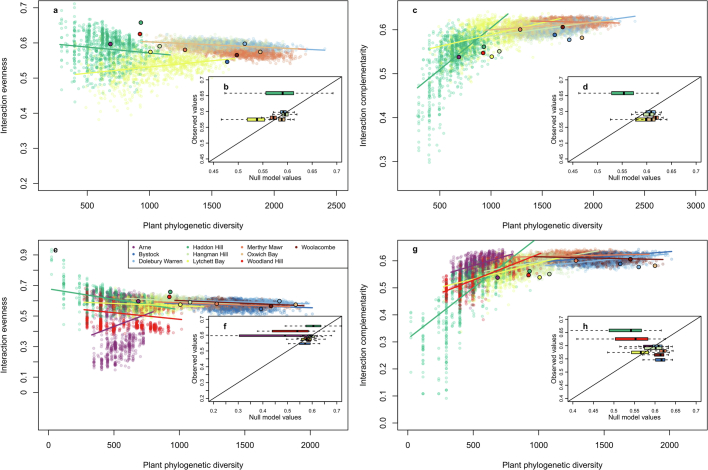
Null model 3: Preserving sampling completeness. Null Model results while additionally constraining for equal sampling completeness: Interaction evenness (A and E) and functional dispersion (C and G) as a function of plant phylogenetic diversity in random triads (coloured points) and observed triads (circles with coloured fill). Two variants of the same null model are displayed here, one only controlling only for the number of interactions (first row), and the other controlling also for the number of plant species. Within each plot, insets (B, D, F, H) compare the expected value of interaction evenness and functional dispersion against the observed values. For each boxplot (1 per site), n = 1,000 replicates. Boxes represent the 25% (Q1) and 75% (Q3) quartiles around the median line, and whiskers are Q1 − 1.5xIQR to Q1 and Q3 to Q3 + 1.5xIQR.

Following the construction of each null triad, we calculated the network properties of interest (interaction evenness and interaction complementarity) and compared them to those of the observed triads. The boxplots in the insets of Fig. 4 and Extended Data Fig. 8 show the range of values for a given network value that we can reasonably expect for a given site under the null hypothesis. If the boxplot overlaps the 1:1 line (that is, *x* = *y* line), then the observed value falls within the null hypothesis. If it is below that line, the observed value is less than under the null hypothesis and, if above, more than under the null hypothesis.

#### Evaluating phylogenetic diversity

To account for the effect of plant phylogenetic diversity in the empirical and simulated triads, we constructed plant phylogenetic trees for each community and measured their phylogenetic diversity as the mean tree branch length. To build one tree per community, we cropped the Daphne phylogeny, a comprehensive dated phylogeny of the European flora^54^, to only include plant species available in each of our communities. Our visitation dataset contains 149 plant species, out of which 139 species (93%) can be found in the Daphne phylogenetic tree. We standardized synonyms for species that were separately identified using Global Biodiversity Information Facility (GBIF). The remaining ten plant species that are not recorded in the Daphne phylogeny can be divided into two groups as follows (Supplementary Table 1):

1. Plant species that are the only representatives of their genus in our pollination dataset (*n* = 3).
2. Plant species that are not the only representatives of their genus in the dataset (*n* = 7).

These plant species were assigned an alternative replacement species, in their genus, selected from the available species in the phylogeny dataset. The replacements for species in the first group were randomly chosen, as fine-scale phylogenetic distances will probably be less important when a genus is represented by a single species in the data. The replacements for species in the second group were chosen more carefully, as they co-occur with congeneric species, following three steps:

(i) We selected the species from the focal genus available in Daphne phylogeny which were not already part of the interaction dataset.
(ii) In GBIF we obtained the coordinates of the occurrences—if any—of these possible species in the United Kingdom.
(iii) For each of the seven species in the second group, we ordered their alternative species from the most to least likely species (measured as number of occurrences in our sampling area). We then selected the top most likely species, which had at least twice as many occurrences as the next likely species. Therefore, the selected species had similarly high occurrences, whereas non-selected species had half or fewer occurrences than the selected species with least occurrences.

If a community included species from the second group and for these species more than one alternative was selected, alternative trees for that community were built and their mean phylogenetic diversity calculated.

We calculated repeated measures correlations between plant phylogenetic diversity and both interaction evenness and interaction complementarity using rmcorr^55^.

### Reporting summary

Further information on research design is available in the Nature Portfolio Reporting Summary linked to this article.

## Online content

Any methods, additional references, Nature Portfolio reporting summaries, source data, extended data, supplementary information, acknowledgements, peer review information; details of author contributions and competing interests; and statements of data and code availability are available at 10.1038/s41586-024-07825-y.

## Supplementary information

Supplementary Information Supplementary Information sections 1–7, including Figs. 1–14, Tables 1–3 and references.

Reporting Summary

Peer Review File

## Data Availability

All data and code are available at Zenodo (10.5281/zenodo.11184586)^43^.
