# Supplementary material for: Multi-habitat landscapes are more diverse and stable with improved function
Source: Nature. 2024 Aug 21;633(8028):114–9. doi: 10.1038/s41586-024-07825-y (PMC11374697; doi:10.1038/s41586-024-07825-y)
Supplement: Supplementary file 1 — Supplementary Information sections 1–7, including Figs. 1–14, Tables 1–3 and references. [file 41586_2024_7825_MOESM1_ESM.pdf]

nature portfolio

https://doi.org/10.1038/s41586-024-07825-y

Supplementary information

Multi-habitat landscapes are more diverse and stable with improved function

In the format provided by the authors and unedited

Supplementary Information

1. Variation associated with shared habitats ... 2
2. Interaction complementarity ... 4
Supplementary Figure 1: Evaluation of functional space ... 4
3. Null model: Additive effects vs. emergent properties of multiple habitats ... 6
Supplementary Figure 2: Area-habitat relationship ... 6
Supplementary Figure 3: Sampling coverage curves of the number of pollination interactions in various habitats and sites ... 9
Supplementary Figure 4: Sampling completeness curves of the number of distinct pollination interactions for grassland in one monad (Wyeswood Common) and one triad (Bystock) ... 10
Supplementary Figure 5: Sampling completeness and necessary number of interaction events.. 11
Supplementary Table 1: Plant species present in the interaction dataset which were not present in the phylogeny dataset ... 12
4. The potential effect of surrounding habitat patch size ... 13
Supplementary Figure 6: Patch area of the surrounding habitat for all monad sites plotted against six structural metrics ... 14
5. The potential effects of sampling completeness on interaction evenness and robustness 15
Supplementary Figure 7: Boxplots of the interaction sampling coverage for monads, dyads and triads ... 15
Supplementary Figure 8: Robustness calculations and sampling coverage ... 16
Supplementary Figure 9: Interaction accumulation curves ... 17
6. Network structural analysis ... 18
Supplementary Table 2: Structural MANOVA output. ... 18
Supplementary Figure 10: Leaf miners and parasitoids structural box plots ... 19
Supplementary Figure 11: Caterpillars and parasitoids structural box plots ... 20
Supplementary Figure 12: Seed feeders and parasitoids structural box plots ... 21
Supplementary Figure 13: Herbivores structural box plots ... 22
Supplementary Figure 14: Parasitoids structural box plots ... 23
7. Field site details ... 24
Supplementary Table 3. The 30 field sites, their habitat composition and GPS coordinates. ... 24
Supplementary information references ... 25

1. Variation associated with shared habitats

We sampled at sites with one, two or three habitats. As this number increases, drawing from a finite pool of habitat types, the probability that pairs of sites will have one or more habitats in common will also increase (i.e. a pair of dyads or triads could contain one, two or three habitats in common) as a simple sampling effect. Therefore, it is possible that, on average, pairs of dyads or triads could be more similar to each other because they will share a proportion of the same habitat type more frequently. We mitigated against this effect in our study design by:

1. Having twice as many habitats in our pool (n = 6) as the maximum at individual sites (n = 3), such that there was a diverse mix of habitat combinations among our replicates.
2. Distributing our sites across a large geographic area and thus variable regional species pools, so that the same habitat type at different locations will not be an identical 'treatment'

To check that an increasing frequency of shared habitat types was unlikely to have caused the decreased variability in robustness we observed moving from monads to triads, we tested two aspects of variability using the floral abundance at each site. First, we used variance partitioning to estimate how much variation in the plant communities among triads could be accounted for by the study site versus the habitat type, using the 'varpart' function in the R package *vegan*[1], with Bray-Curtis dissimilarities. This indicated that site (14.4%) accounted for almost as much variation in plant communities as habitat type (17.9%), highlighting the effect of sampling across a large geographical extent (point 2 above). This indicates that even where two sampling sites shared habitat types, substantial variation in the plant community was still present.

Secondly, again using Bray-Curtis dissimilarities, we tested whether beta-diversity between the 10 replicates declined moving from monads, through dyads to triads, consistent with this sampling effect. There was a small decrease in the mean Bray-Curtis dissimilarities (0.88, 0.86 and 0.79 respectively), but this was not statistically-significant (*vegan*'s 'betadisper' function; $F_{2,27} = 0.58$, $p = 0.56$).

We therefore concluded that while an important consideration, the variability effects we detect are unlikely to be explained by multiple habitat sites being simply more similar to each other through overlapping habitat types.

2. Interaction complementarity

Evaluation of functional space

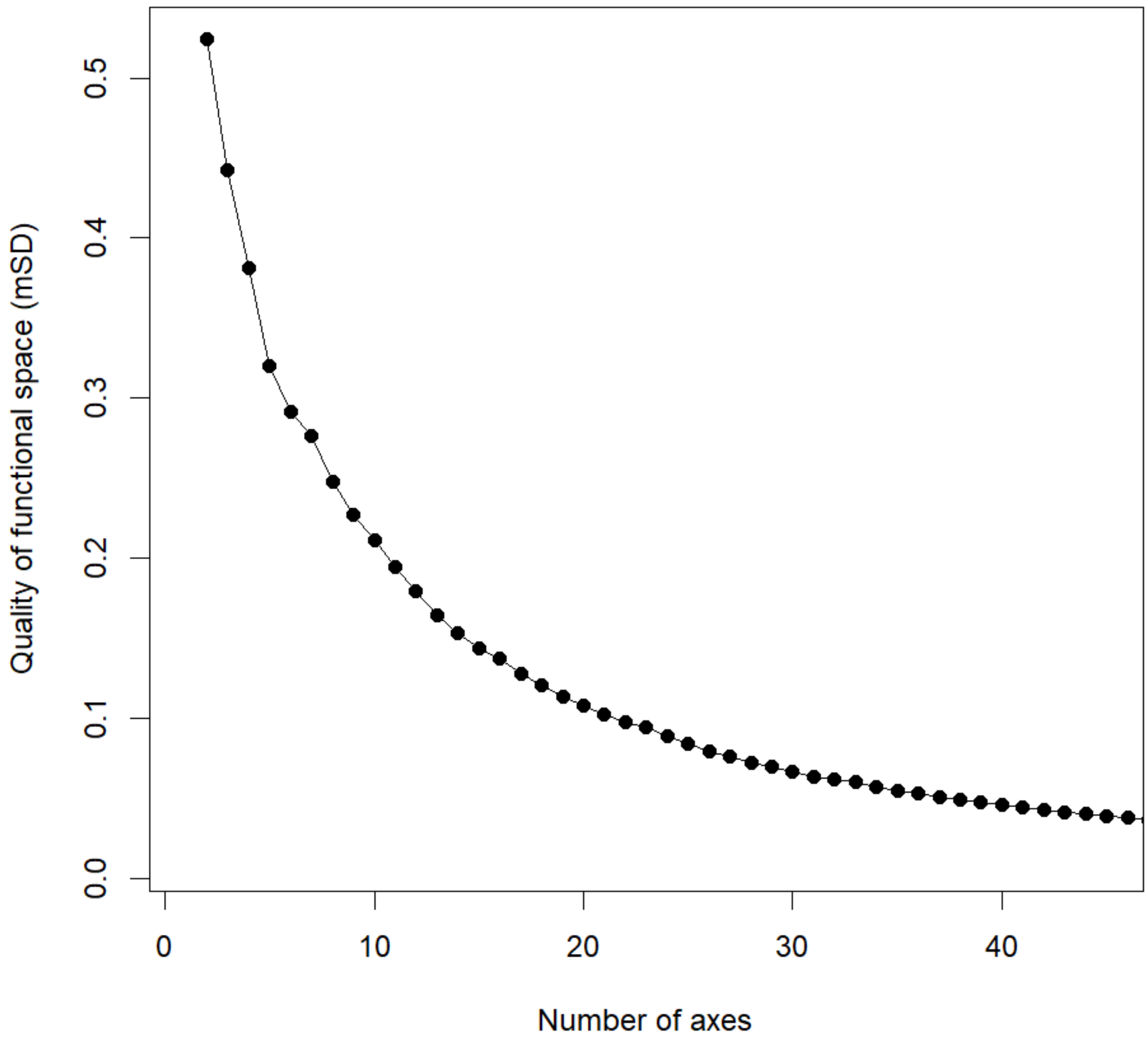

Supplementary Figure 1: Evaluation of functional space

The quality of the functional space as a function of its number of axes. The quality of the functional space is assessed with the mean squared deviation between the interaction dissimilarities of species pairs (Bray-Curtis dissimilarity matrix) and the standardized Euclidean distances between species in the functional space (see[2] for details). The mSD tends towards 0 when interaction dissimilarities are equal to distances in the functional space, *i.e.* distances accurately represent interaction dissimilarities.

Interaction complementarity preserving high sampling completeness

In our dataset, species with low abundance can either be rare and/or under-sampled. In our functional diversity analysis, rare and under-sampled insect species will appear as highly specialised, as few individuals will have been sampled; this will result in a low niche overlap and therefore low interaction similarity, with other insect species. In order to deal with this potential bias, we ran two more-conservative versions of our functional diversity analysis; in these analyses we only included insect species which had both:

1) Ten or more individuals sampled.
2) A sampling completeness, measured as the ratio of an insect species' observed number of interactions with plant species and the estimated (Chao index) number of interactions, of first 50% or higher and then 70% or higher.

These criteria reduced our number of insects from 524 insect species in the complete analysis to 76 when sampling completeness was 50% or higher and 56 species when 70% or higher. Nevertheless, by combining insect abundances with distance, our interaction complementarity calculation is robust to rarity effects, as rare species will contribute less to estimates. We found that, despite drastically reducing our dataset in order to only include well sampled species, habitat diversity continues to have a significant effect on pollinator insect functional diversity (50% sampling completeness: $t_{10} = 8.82$, $p < 0.001$; 70% sampling completeness: $t_{10} = 8.83$, $p < 0.001$). Therefore, even if just the most commonly sampled species are considered, landscapes with multiple habitats support greater interaction complementarity.

3. Null model: Additive effects vs. emergent properties of multiple habitats

Area-habitat relationship

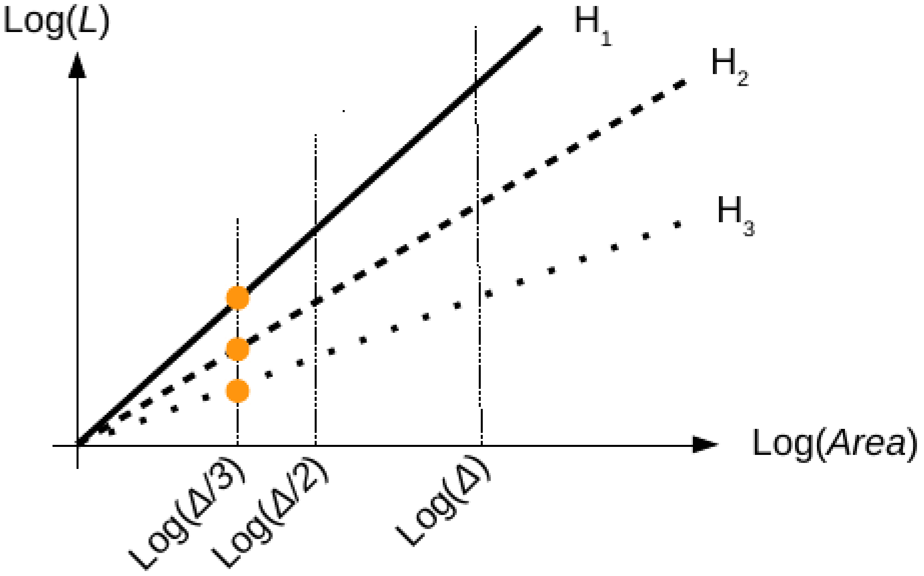

Supplementary Figure 2: Area-habitat relationship

Number of links (L) sampled in three different habitats (H_1, H_2, H_3, ranked from the most to the least interaction rich) against the sampled area. Δ corresponds to the area sampled in each site described in the manuscript. Orange dots correspond to the number of interactions expected if a third of Δ were sampled.

Preserving sampling completeness

Using the same number of recorded interaction events for null triads as empirical triads does not guarantee the same level of sampling completeness, which may affect the measured network properties[3]. The same level of sampling effort may produce different levels of sampling completeness in different sites because they are likely to differ in their population sizes (plants and insects) and relative frequencies of interactions (Supplementary Figure 3). We therefore also ran both null models while preserving the sampling completeness, rather than the number of caught individuals each time.

Two sites, the monad Wyeswood Common and the triad Bystock, provide a good example of this (Supplementary Figure 4); both sites contain grassland areas where plant-flower visitor interactions have been recorded. After estimating the number of distinct interactions with the Chao 1 estimator, we compared the level of sampling completeness of these two grasslands. Sample coverage is 67.4% with 116 captured individuals and 70.9% with 440 individuals in Bystock and Wyeswood Common grasslands, respectively. To achieve the same level of sampling completeness for Bystock grassland when sub-sampling pollination interactions in the grassland part of Wyeswood Common, we would need to collect ~316 interactions/insect individuals. These estimates were calculated with the function 'estimateD' from the R package *iNEXT*[4], with option "base" set to "coverage".

We therefore preserved sampling completeness for each habitat when generating random triads by using an estimate of the number of interaction events to sample in the corresponding monads with the rarefaction/extrapolation approach illustrated in Supplementary Figure 4.

However, this is not possible for all triads. Some of the triad habitats were sampled with a greater coverage proportion than their corresponding monads (e.g. the grassland subset of

Woolacombe (Supplementary Figure 3A), heathland in Bystock and Woodland Hill (Supplementary Figure 3B), and woodland in Arne (Supplementary Figure 3F). Sampling monad interactions to preserve sampling completeness while also preserving the plant community richness within habitats of the null triads (*null model #2*), consequently required additional care. Sub-sampling one reference monad to a given number of plant species may affect its level of sampling completeness (cf., Supplementary Figure 5A for an example). We therefore assessed the required number of interaction events to sample for each monad sub-sampling; this varied for the same monad and number of plant species being retained (Supplementary Figure 5B). As a result, preserving sampling completeness reduced the number of possible sites used in this analysis from 10 to 6 (Extended Data Fig. 8) but the general trend remains.

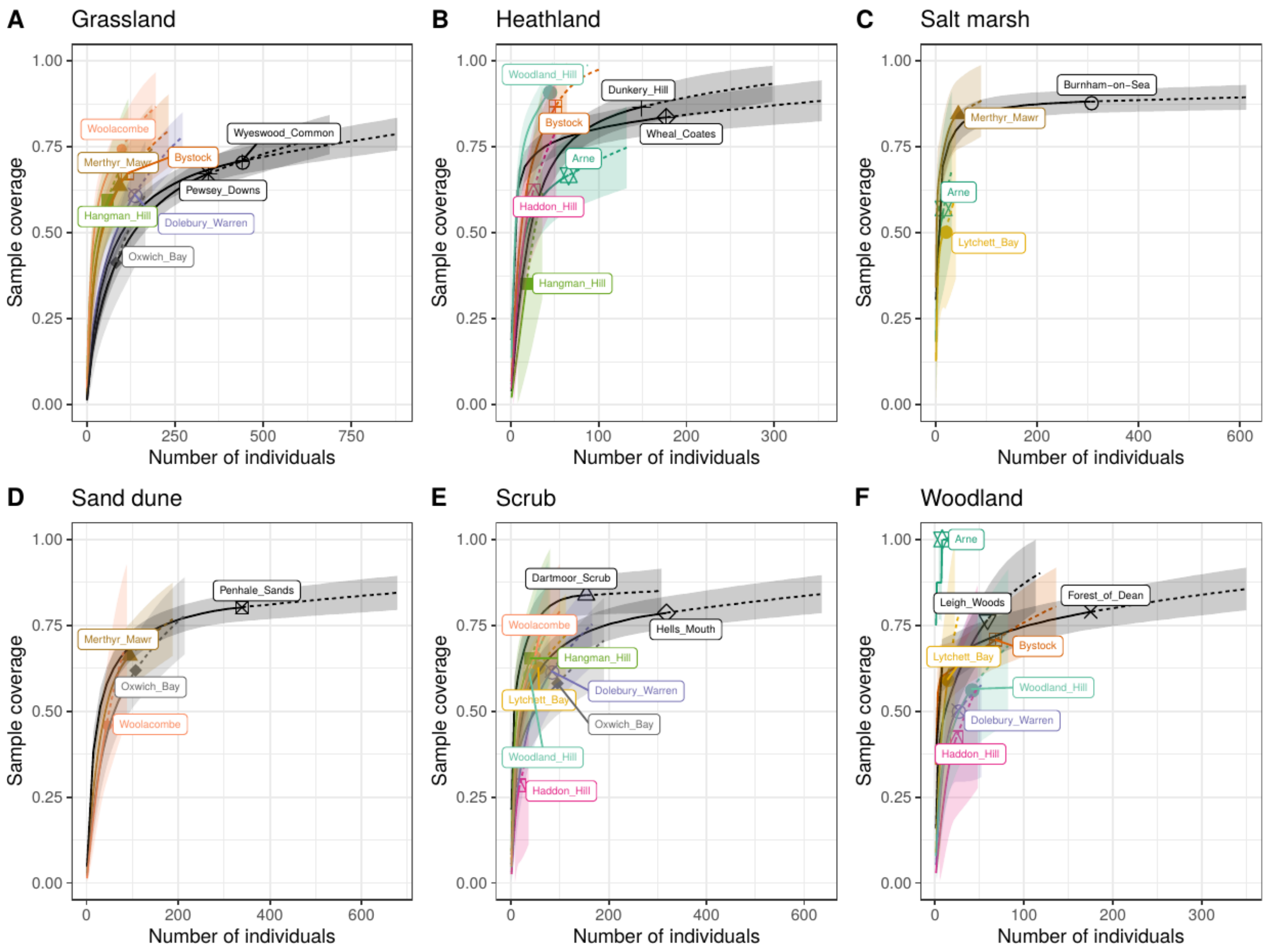

Supplementary Figure 3: Sampling coverage curves of the number of pollination interactions in various habitats and sites

Each symbol corresponds to the observed sampling coverage in one site-habitat pair (or sampling completeness), solid lines are interpolation of the sampling coverage for lower sampling efforts (in number of interaction events recorded, i.e., number of individuals collected) while dashed lines are extrapolations of sampling coverage for higher sampling efforts. Black lines correspond to monads, while coloured ones match the habitat section of various triads. Asymptotes were estimated using a Chao1 estimator, coverage-based sampling curves are drawn based on Chao and Jost's method[5], and confidence intervals around the mean were based on bootstrap sampling[3].

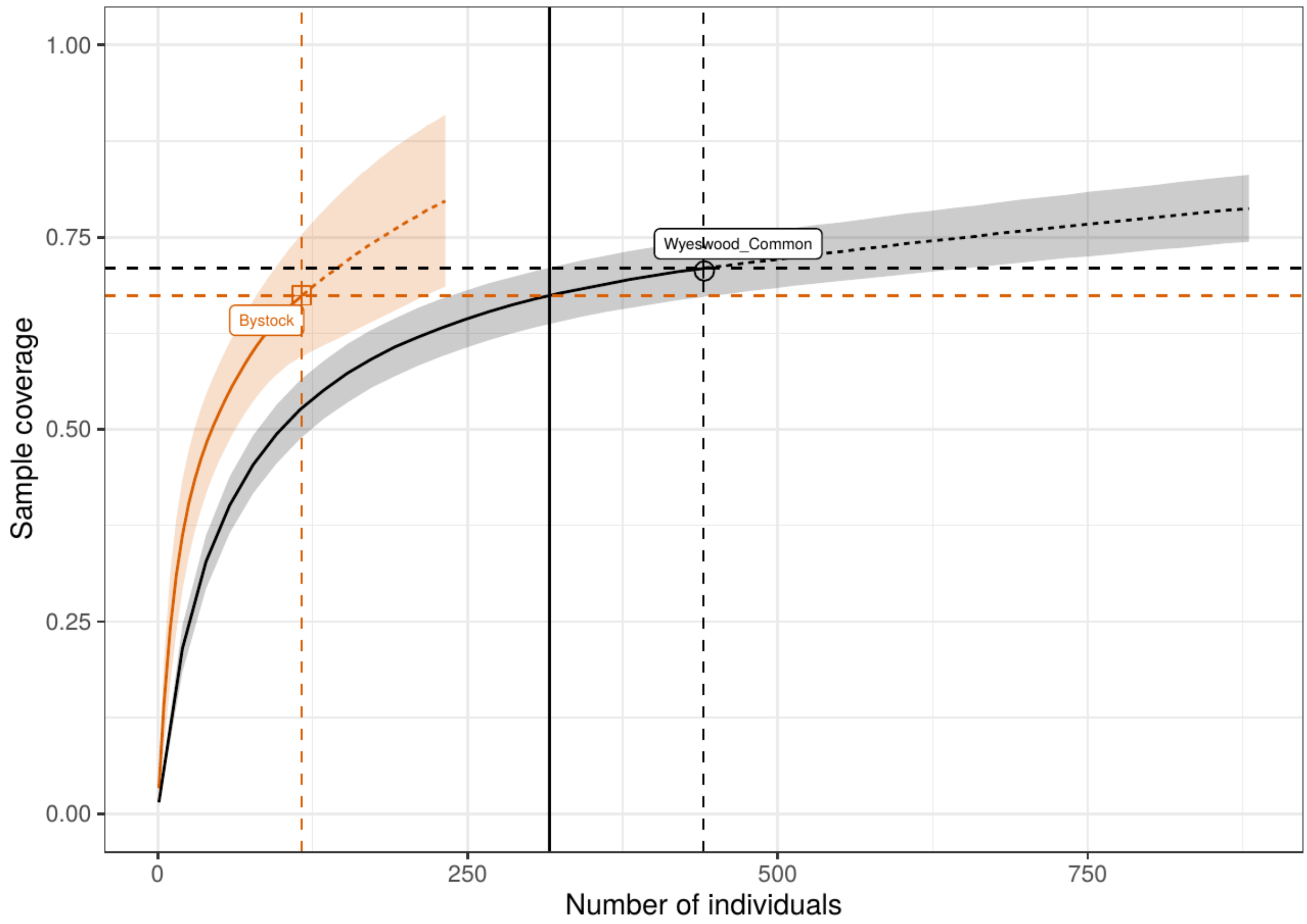

Supplementary Figure 4: Sampling completeness curves of the number of distinct pollination interactions for grassland in one monad (Wyeswood Common) and one triad (Bystock)

Solid curves are interpolation of the sampling coverage for lower sampling efforts (in number of interaction events recorded, i.e., number of individuals collected) while dashed curves are extrapolations of sampling coverage for higher sampling efforts. Asymptotes were estimated using a Chao1 estimator, coverage-based sampling curves are drawn based on Chao and Jost's method[5], and confidence intervals around the mean were based on bootstrap sampling[3]. Dashed vertical lines indicate the number of interaction events recorded in grassland for each site. Dashed horizontal lines indicate the sampling coverage achieved for each. The vertical solid line marks the number of interaction events to sample in Wyeswood Common dataset to attain the same level of sampling completeness as in the grassland of Bystock.

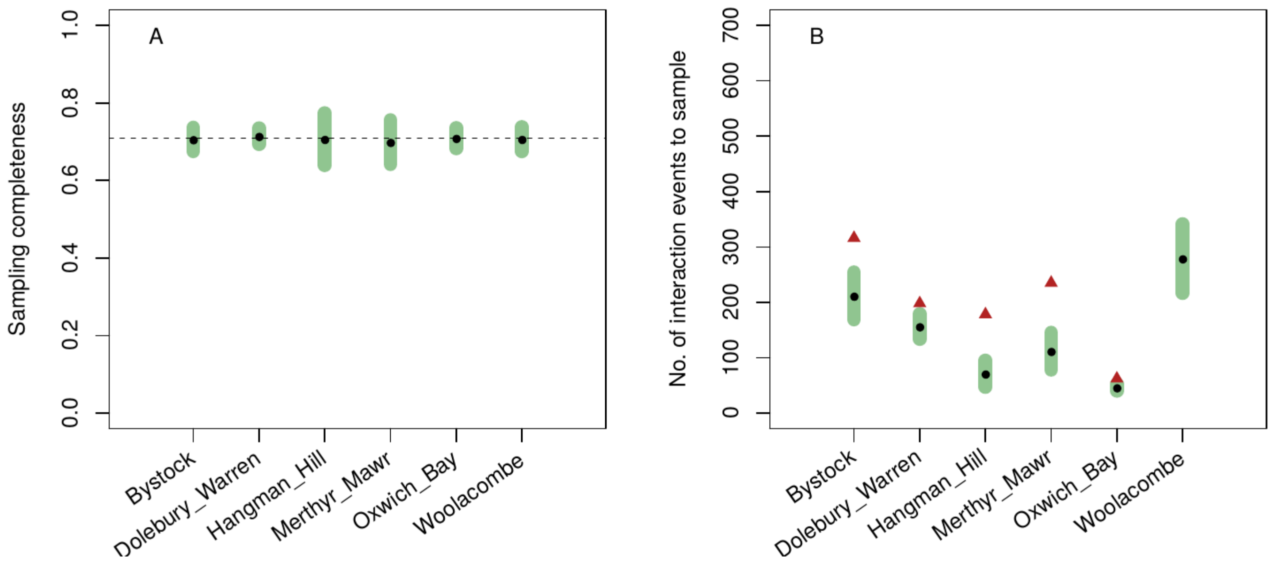

Supplementary Figure 5: Sampling completeness and necessary number of interaction events

A) Sampling completeness of pollination interactions in Wyeswood Common when sub-sampling interactions to a number of plant species that is the same as in triads comprising grassland (named on the x-axis). This, in turn, affects B) the number of interaction events to sample while preserving the same level of sampling completeness as in the sub-triads. To generate these graphs, we repeat the sub-sampling of pollination interactions for Wyeswood Common 100 times for a given number of plant species for each triad. Black circles indicate either the mean of the resulting sampling completeness (A) or the mean of the number of interaction events (B) to sample to preserve the same level of sampling completeness as the corresponding triad (x-axis) in Wyeswood Common. Note the large error bars in green. Red triangles indicate the number of interaction events to sample in the dataset of Wyeswood Common if the whole plant community were considered.

Plant phylogenetic diversity

Supplementary Table 1: Plant species present in the interaction dataset which were not present in the phylogeny dataset

Species fall into two groups: the single representatives of their genus in the plant-pollinator interaction dataset (group 1); not the single representatives of their genus in the plant-pollinator interaction dataset (group 2).

Species	***Problem***	***Solution***
Convolvulus vulgaria	alternative	*Convolvulus cantabrica*
Crataegus sp.	alternative	*Crataegus laevigata*
Heracleum sp.	alternative	*Heracleum mantegazzianum*
Hieracium agg.	alternative	*Hieracium sabaudum*
		Hieracium umbellatum
		Hieracium maculatum
Oenanthe sp.	alternative	*Oenanthe pimpinelloides*
		Oenanthe fistulosa
		Oenanthe lachenalli
Oxalis perennis	alternative	*Oxalis corniculata*
Primula arvensis	alternative	*Primula vulgaris*
Rhododendron sp.	genus	randomly sampled *Rhododendron* species
Taraxacum agg.	genus	randomly sampled *Taraxacum* species
Thymus_polytrichus	genus	randomly sampled *Thymus* species

4. The potential effect of surrounding habitat patch size

To ensure that our results were not confounded by the size of the surrounding habitat patch[6], for all monads, we calculated the contiguous patch size of the surrounding habitat (Grassland, Heathland, Salt Marsh, Sand Dune, Scrub, and Woodland) on ArcGIS v.10.8. We used the Land Cover 2007 data[7] for 7 sites (confirmed against ground truthing, photos and site maps). For the remaining 3 sites ("Wyeswood Common", "Pewsey Down" and "Dartmoor Scrub"), the Land Cover data did not match our ground truthing, so we calculated the surrounding area using a combination of photos, site maps, Google Earth data and details from Natural England or the Gwent Wildlife Trust. We then plotted the surrounding patch size against our six structural measures included in the MANOVA, indicating the habitat to confirm there was indeed no confounding effect of patch size on any metric (Supplementary Figure 6).

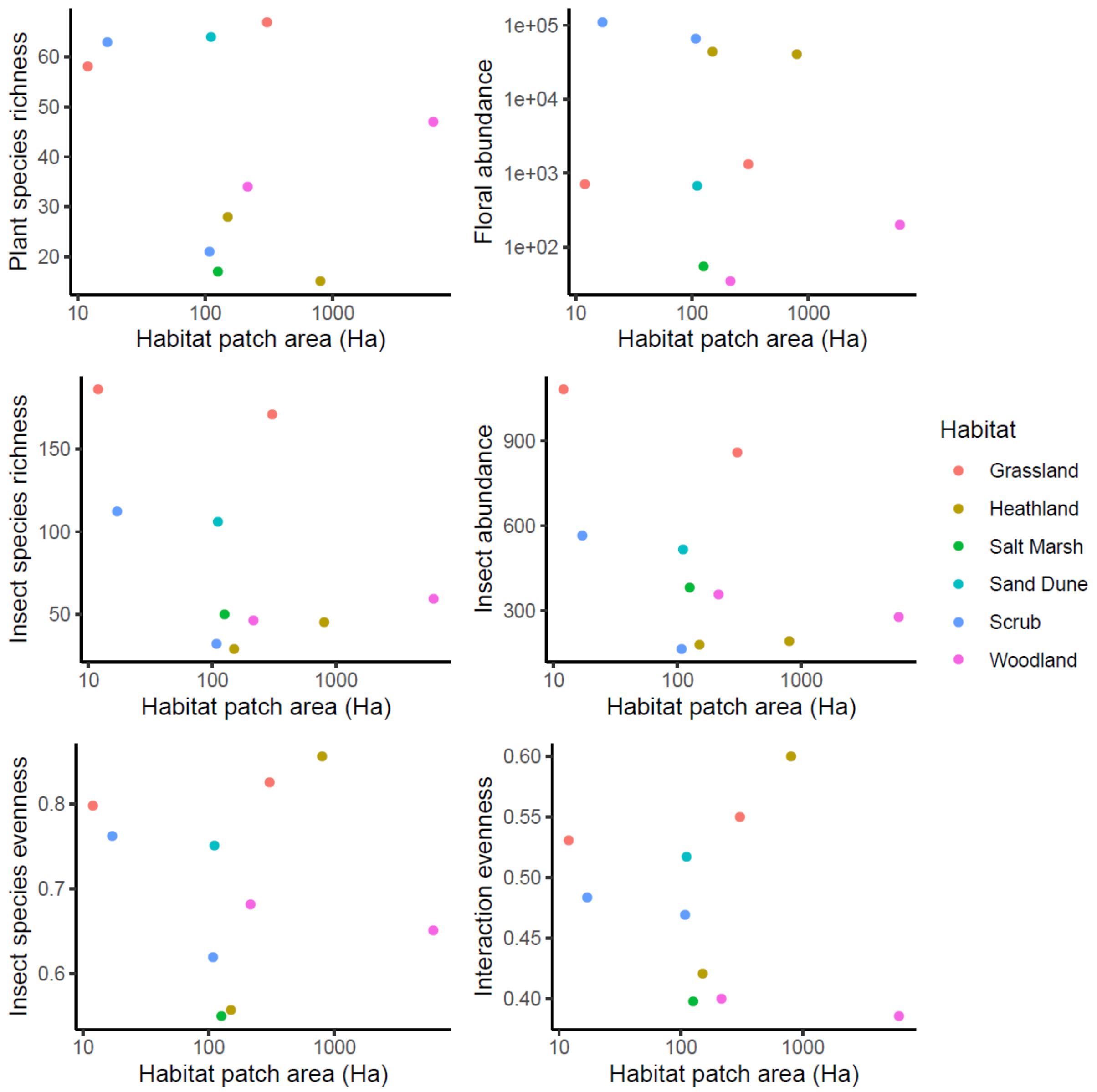

Supplementary Figure 6: Patch area of the surrounding habitat for all monad sites plotted against six structural metrics

Surrounding patch area plotted against plant species richness, floral abundance, insect species richness, insect species evenness and interaction evenness. The habitat (grassland, heathland, salt marsh, sand dune, scrub and woodland) is indicated by the colour of the point. There is no influence of patch size on any of the extracted metrics.

5. The potential effects of sampling completeness on interaction evenness and robustness

The effect of both species and interaction sampling completeness are important considerations in network ecology. Therefore, our study design ensured that sampling effort was equal across all sites, such that differences in abundances and frequencies would represent real differences. However, controlling for sampling effort does not prevent differences in sampling completeness associated with specific within-site habitats (Supplementary Figure 7).

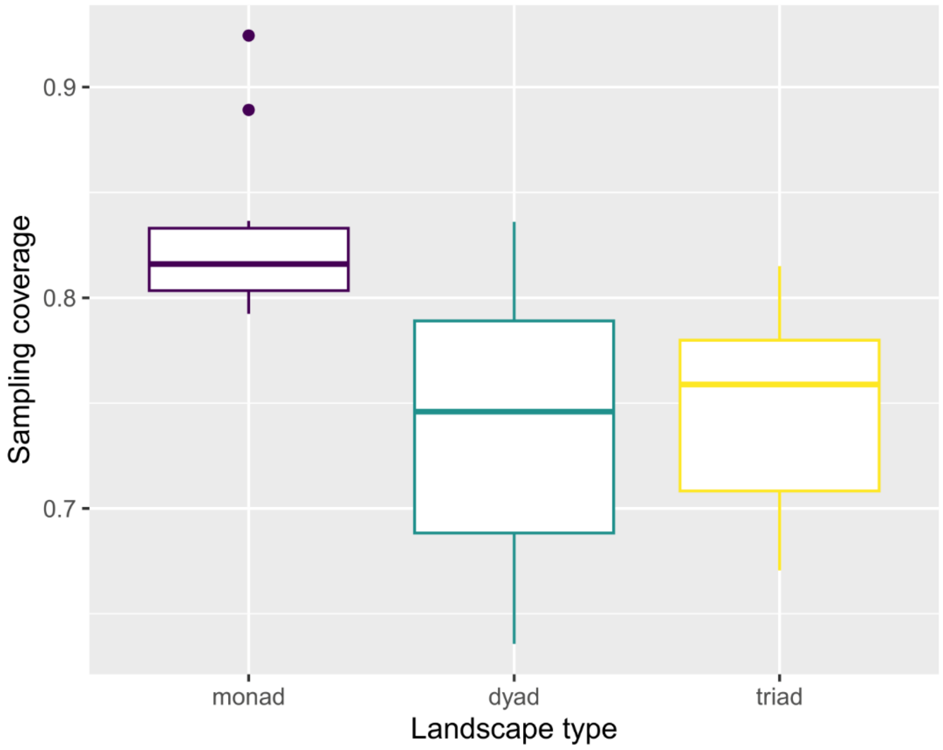

Supplementary Figure 7: Boxplots of the interaction sampling coverage for monads, dyads and triads

Monads have higher interaction sampling completeness than dyads and triads. For each box plot n = 200 (10 sites x 20 replicates). Boxes represent the 25% (Q1) and 75% (Q3) quartiles around the median line, and whiskers are Q1-1.5xIQR to Q1 and Q3 to Q3+1.5xIQR.

One way in which we addressed this problem was by using quantitative network metrics (e.g. robustness and interaction evenness) which are less biased by sampling incompleteness[3,8,9]. Moreover both interaction evenness and robustness are less sensitive to sampling completeness

differences[8,10]. Nevertheless, to investigate if sampling completeness differences were significantly contributing to our results, we plotted both robustness and interaction evenness against interaction sampling completeness, with colour-coded datapoints for monads, dyads and triads (Supplementary Figure 8). There is no relationship between sampling completeness and robustness and this relationship does not change between site types. For interaction evenness, we ran a repeated measures correlation analysis testing for a correlation between interaction evenness and sampling completeness across replicated monads, dyads and triads (Supplementary Figure 8). This also showed no significant correlation (r_{26} = -0.257; p = 0.187).

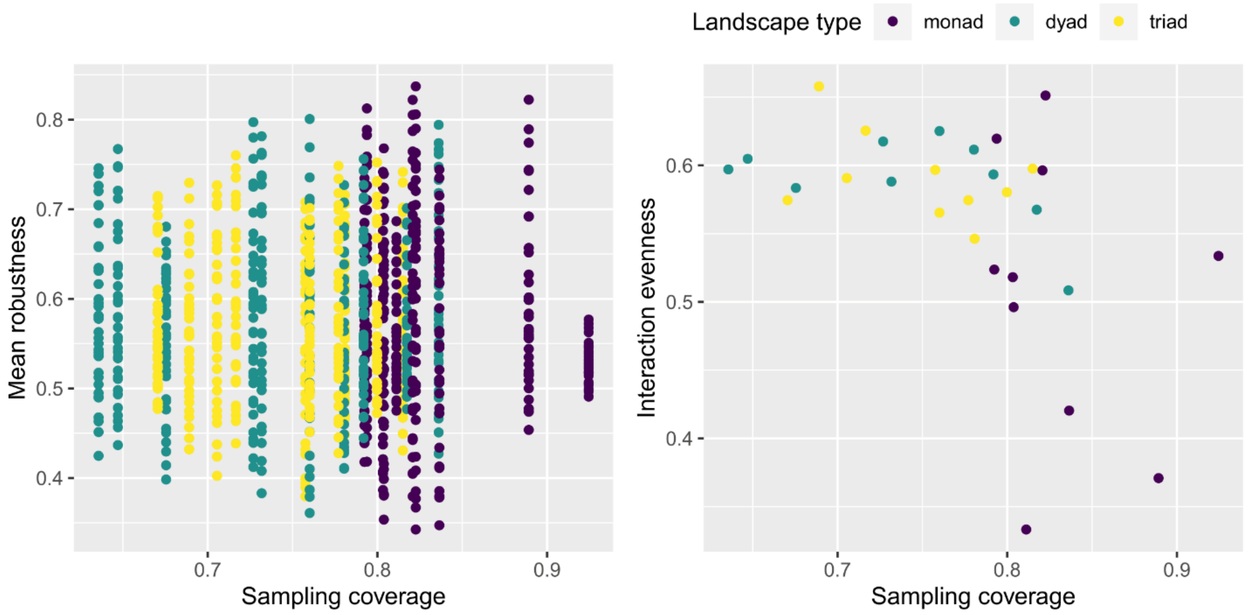

Supplementary Figure 8: Robustness calculations and sampling coverage
Robustness calculations of all extinction and threshold scenarios (left) for each monad (purple), dyad (teal) and triad (yellow) plotted against interaction sampling coverage. Robustness does not correlate with differences in sampling completeness. Interaction evenness (right) for each monad, dyad and triad plotted against sampling completeness. A repeated measures correlation analysis shows that this correlation is also not significant.

We finally confirmed that there was no confounding effect of sampling completeness on interaction evenness by simulating interaction accumulation using the iNext package[4] (20

replicates for each of the 24 sampling completeness values tested at each site). Although rarefying the networks might reveal some relationship between increasing sampling completeness and interaction evenness, this is not consistent. The effect ranges from no effect to a larger one when networks are particularly species poor, and the direction of the effect is variable. Moreover, restricting measurements to an equal level of completeness (e.g. 75%) would not affect our conclusions (Supplementary Figure 9).

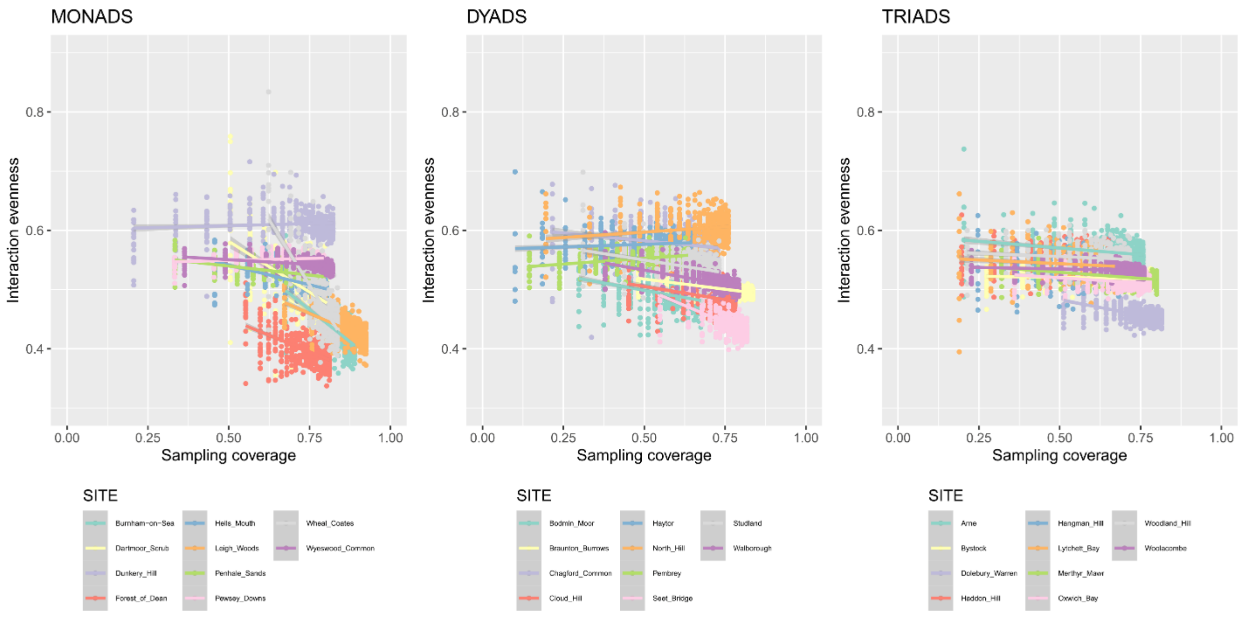

Supplementary Figure 9: Interaction accumulation curves

Interaction accumulation curves with interaction evenness calculations plotted against sampling completeness for each monad (left), dyad (middle) and triad (right) site. For each site, networks were subsampled (20 times) to 24 values of sampling completeness below the maximum attained when preserving sampling effort. Although rarefying the networks may show some relationship between increasing sampling coverage and interaction evenness, it is not consistent and if sampling completeness were calculated at a consistent level (e.g. 75%), the conclusions would not change.

6. Network structural analysis

Supplementary Table 2: Structural MANOVA output.

Output from structural MANOVA. All tests are 2-tailed and unadjusted p-values are reported. Post-hoc results following a significant MANOVA remain significant with Bonferroni adjustment for the 6 structural metrics. Thus, for All Sites, Insect species evenness: adjusted p = 0.0042 and for Monads v. Triads, insect species evenness: adjusted p = 0.018. MANOVA was not significant for Monads v. Dyad nor Dyads v. Triads.

	df	*F*	*p*	
All Sites				
MANOVA	**1,28**	**5.65**	**0.0018**	**
Floral abundance	1,28	0.18	0.674	
Plant species richness	1,28	3.24	0.082	.
Insect abundance	1,28	0.71	0.405	
Insect species richness	1,28	0	1	
Interaction evenness	1,28	3.16	0.086	.
Insect species evenness	**1,28**	**14.92**	**0.0007**	***
Monads v. Dyads				
MANOVA	1,18	1.48	0.259	
Floral abundance	1,18	0.22	0.657	
Plant species richness	1,18	0.04	0.847	
Insect abundance	1,18	0.73	0.405	
Insect species richness	1,18	0.07	0.799	
Interaction evenness	1,18	2.48	0.133	
Insect species evenness	1,18	6.86	0.017	
Monads v. Triads				
MANOVA	**1,18**	**4.01**	**0.017**	*
Floral abundance	1,18	0.17	0.689	
Plant species richness	1,18	3.26	0.088	
Insect abundance	1,18	0.73	0.405	
Insect species richness	1,18	0	1	
Interaction evenness	1,18	3.83	0.066	.
Insect species evenness	**1,18**	**12.231**	**0.003**	**
Dyads v. Triads				
MANOVA	1,18	1.4	0.286	
Floral abundance	1,18	0.01	0.91	
Plant species richness	1,18	2.41	0.138	
Insect abundance	1,18	0.01	0.937	
Insect species richness	1,18	0.11	0.742	
Interaction evenness	1,18	0.015	0.903	
Insect species evenness	1,18	1.65	0.215	

Leaf miners and parasitoids

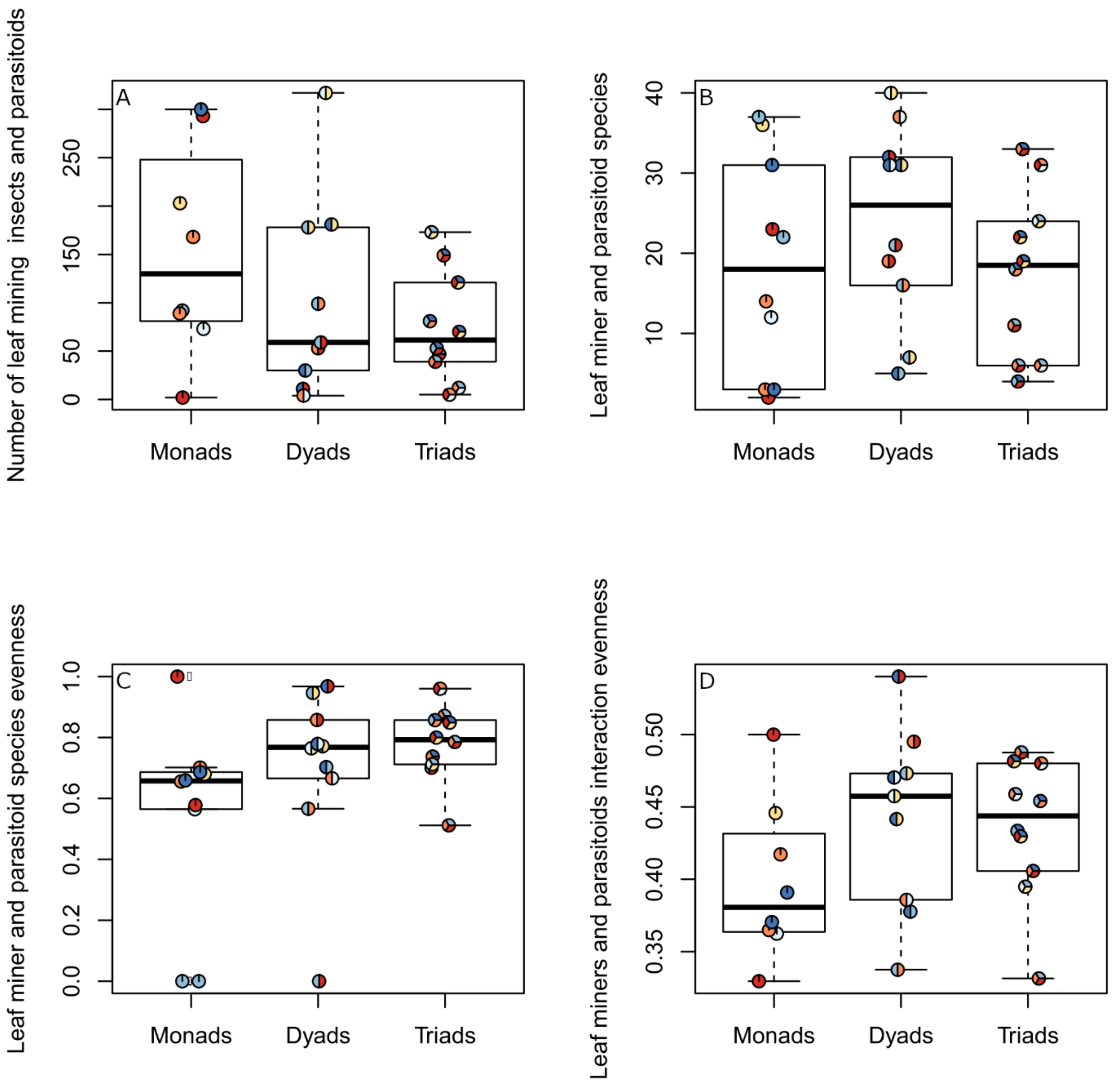

Supplementary Figure 10: Leaf miners and parasitoids structural box plots

Differences among leaf mining insects and their parasitoids at monads, dyads and triads in terms of: A. abundance B. species richness, C. interaction evenness and D. species evenness. Circles indicate each site and the habitat combination therein with a random horizontal jitter to reduce overlap. Data are from 30 independent sites, 2,345 plant-leaf miner interactions (149 species) and 557 leaf miner-parasitoid interactions (84 species). Boxes represent the 25% (Q1) and 75% (Q3) quartiles around the median line, and whiskers are Q1-1.5xIQR to Q1 and Q3 to Q3+1.5xIQR.

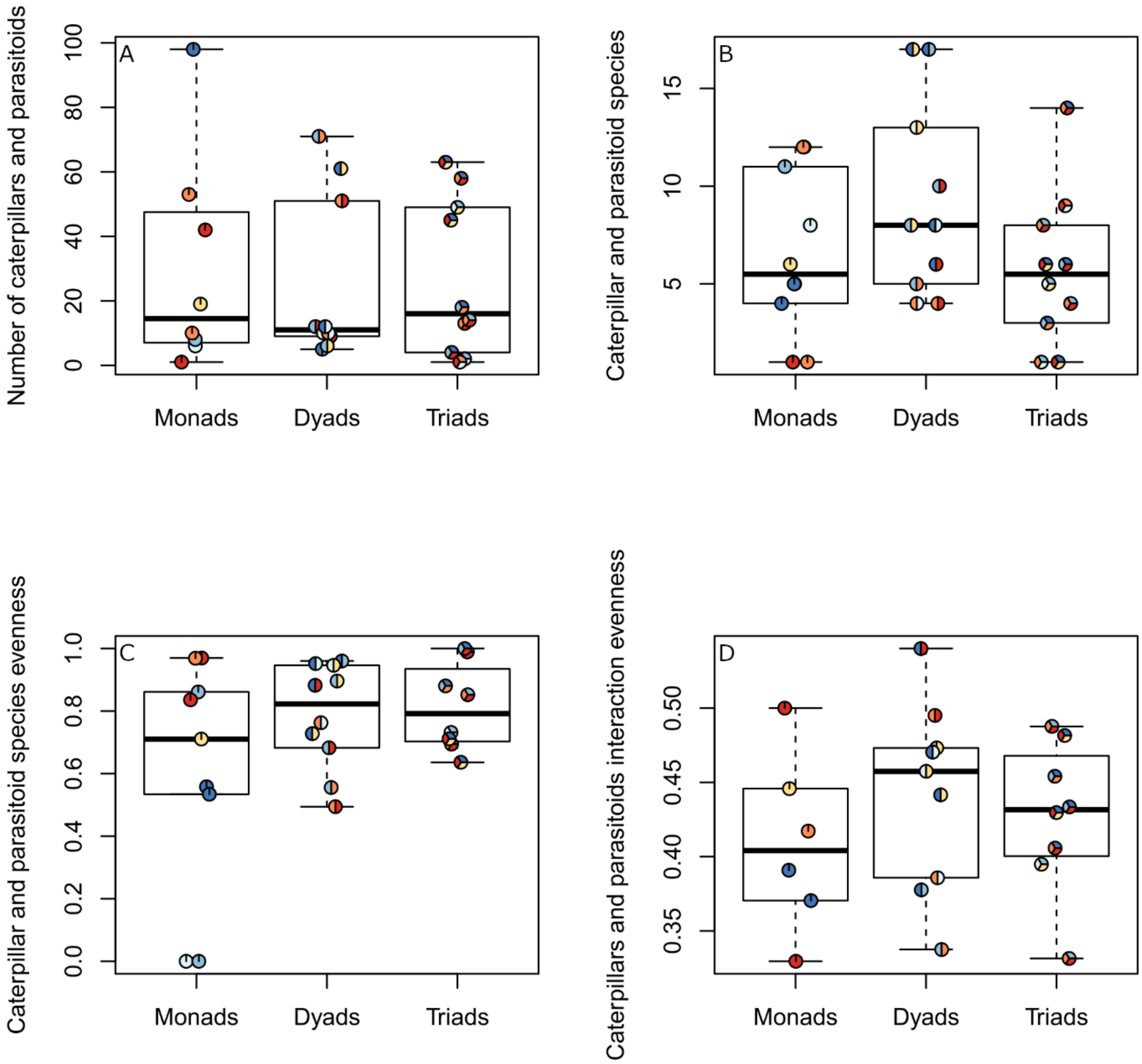

Supplementary Figure 11: Caterpillars and parasitoids structural box plots

Differences among caterpillars and their parasitoids at monads, dyads and triads in terms of: A. abundance B. species richness, C. interaction evenness and D. species evenness. Circles indicate each site and the habitat combination therein with a random horizontal jitter to reduce overlap. Data are from 30 independent sites, 697 plant-caterpillar interactions (90 species) and 54 caterpillar-parasitoid interactions (27 species). Boxes represent the 25% (Q1) and 75% (Q3) quartiles around the median line, and whiskers are Q1-1.5xIQR to Q1 and Q3 to Q3+1.5xIQR.

Seed feeders and parasitoids

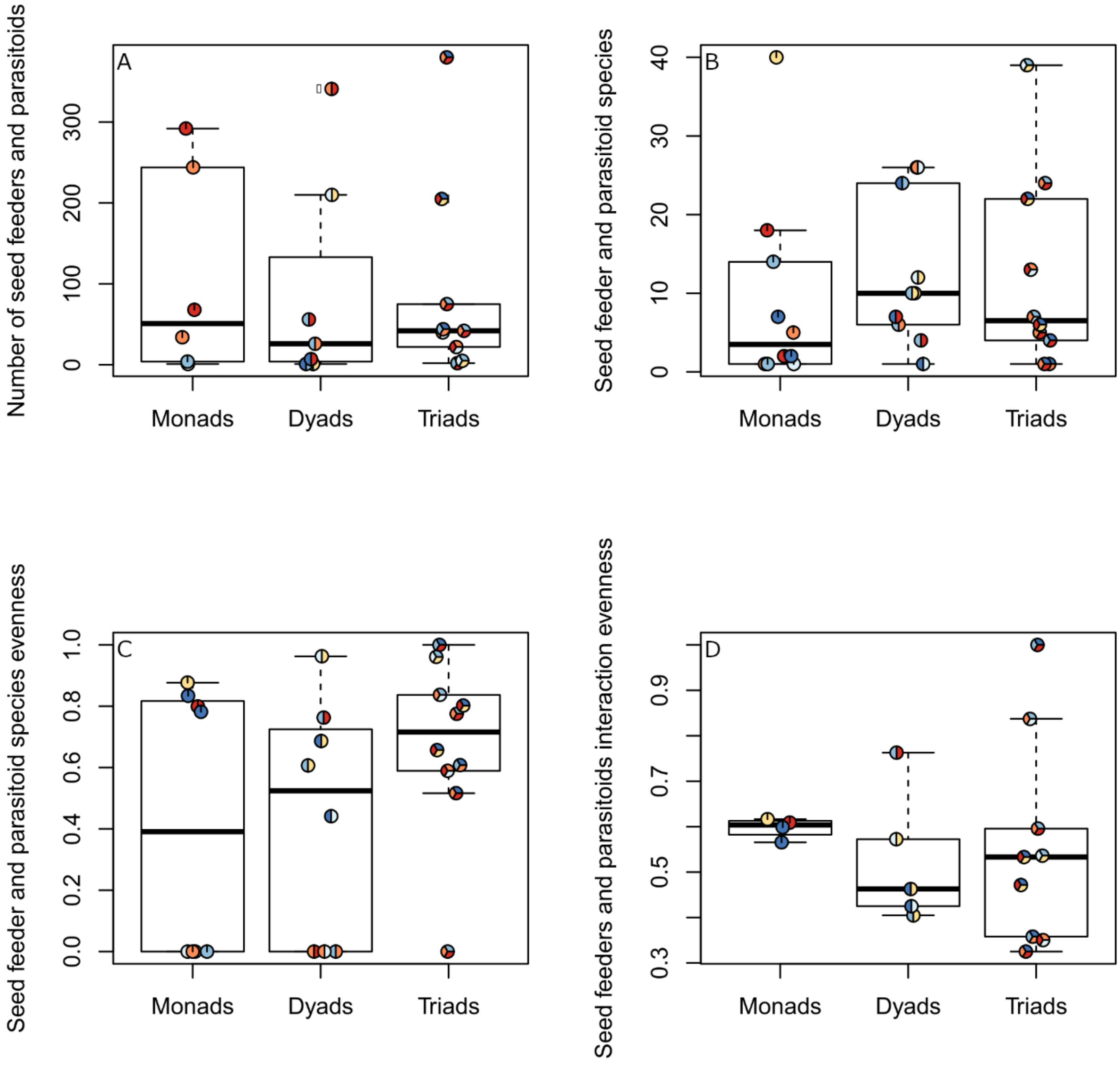

Supplementary Figure 12: Seed feeders and parasitoids structural box plots

Differences among seed feeding insects and their parasitoids at monads, dyads and triads in terms of: A. abundance B. species richness, C. interaction evenness and D. species evenness. Circles indicate each site and the habitat combination therein with a random horizontal jitter to reduce overlap. Data are from 30 independent sites, 1,240 plant-seed feeder interactions (61 species) and 860 plant-parasitoid interactions (54 species). Boxes represent the 25% (Q1) and 75% (Q3) quartiles around the median line, and whiskers are Q1-1.5xIQR to Q1 and Q3 to Q3+1.5xIQR.

Herbivores

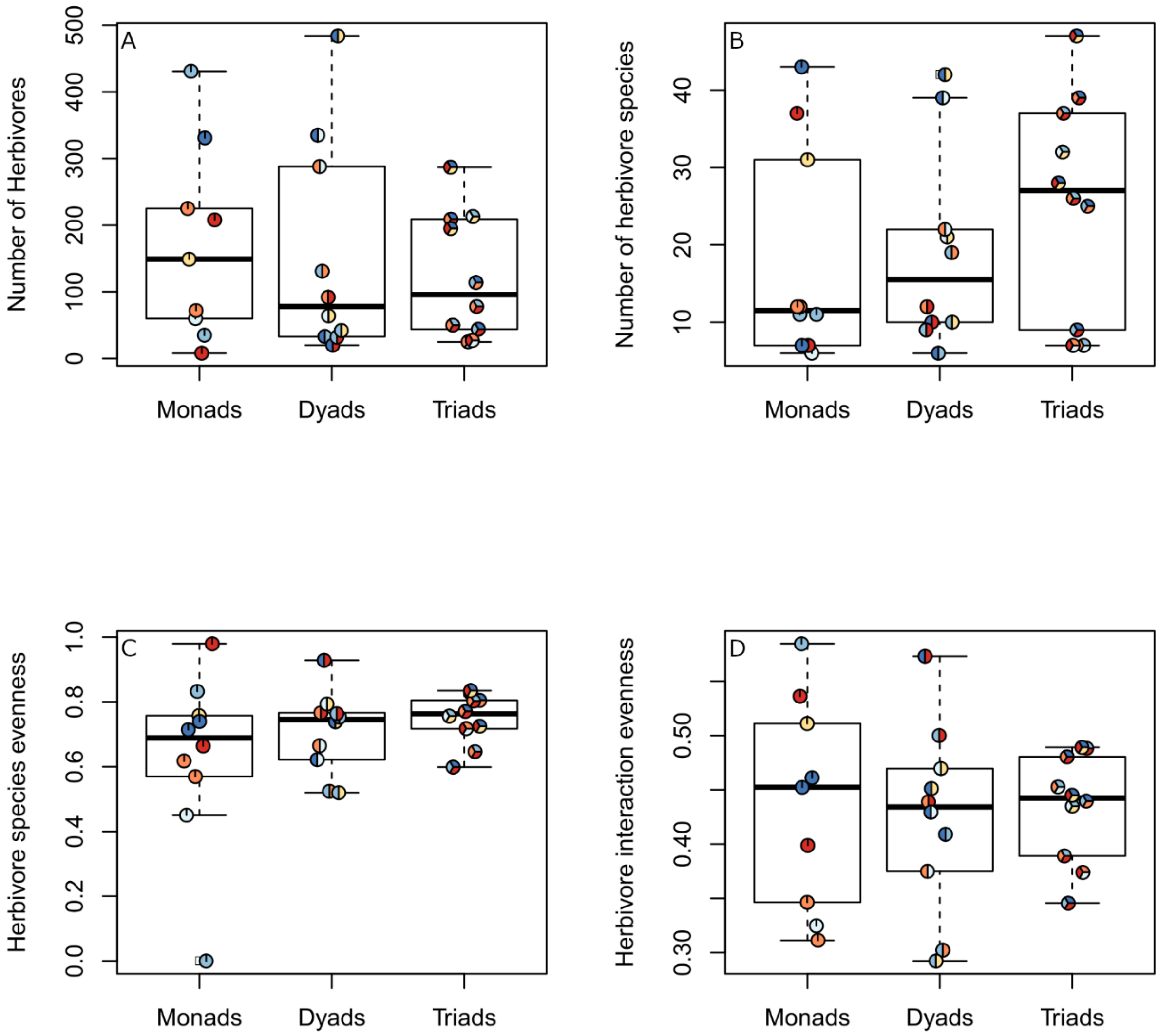

Supplementary Figure 13: Herbivores structural box plots

Differences among herbivores at monads, dyads and triads in terms of: A. abundance B. species richness, C. interaction evenness and D. species evenness. Circles indicate each site and the habitat combination therein with a random horizontal jitter to reduce overlap. Data are from 30 independent sites, 4,282 plant-herbivore interactions (300 species). Boxes represent the 25% (Q1) and 75% (Q3) quartiles around the median line, and whiskers are Q1-1.5xIQR to Q1 and Q3 to Q3+1.5xIQR.

Parasitoids

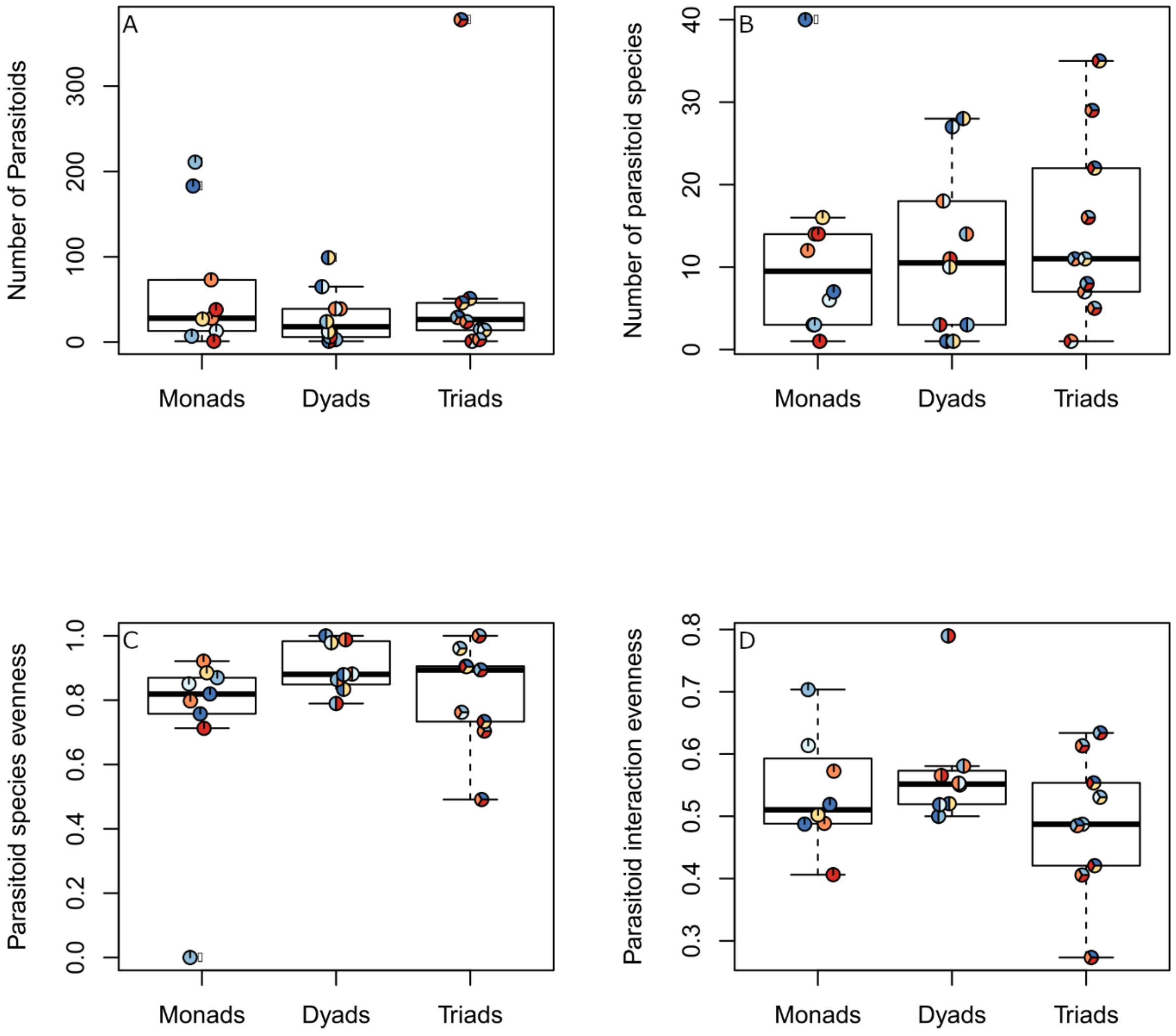

Supplementary Figure 14: Parasitoids structural box plots

Differences among parasitoids at monads, dyads and triads in terms of: A. abundance B. species richness, C. interaction evenness and D. species evenness. Circles indicate each site and the habitat combination therein with a random horizontal jitter to reduce overlap. Data are from 30 independent sites, 1,471 herbivore-parasitoid interactions (165 species). Boxes represent the 25% (Q1) and 75% (Q3) quartiles around the median line, and whiskers are Q1-1.5xIQR to Q1 and Q3 to Q3+1.5xIQR.

7. Field site details

Supplementary Table 3. The 30 field sites, their habitat composition and GPS coordinates.

Type	*Habitat(s)*	*Site name*	*Latitude*	*Longitude*
Monad	Sand Dune	Penhale Sands	50°21'57.03"N	5° 8'51.01"W
Monad	Salt Marsh	Burnham-on-sea	51°13'23.34"N	3° 0'3.57"W
Monad	Heathland	Dunkery Hill	51°10'2.03"N	3°34'16.95"W
Monad	Heathland	Wheal Coates	50°18'8.98"N	5°13'58.27"W
Monad	Scrub	Hudder Down	50°14'18.10"N	5°21'24.01"W
Monad	Scrub	East Dartmoor NNR	50°36'9.94"N	3°44'9.91"W
Monad	Grassland	Pewsey Downs	51°22'12.54"N	1°50'32.29"W
Monad	Grassland	Wyeswood Common	51°46'46.31"N	2°41'28.36"W
Monad	Woodland	Forest of Dean	51°48'45.57"N	2°33'10.79"W
Monad	Woodland	Leigh Woods	51°27'42.77"N	2°38'12.56"W
Dyad	Sand Dune & Grassland	Braunton Burrows	51° 5'25.68"N	4°12'17.30"W
Dyad	Salt Marsh & Grassland	Walborough	51°19'4.91"N	2°59'8.69"W
Dyad	Sand Dune & Heathland	Studland	50°40'28.61"N	1°56'50.10"W
Dyad	Sand Dune & Salt Marsh	Pembry Beach	51°40'21.03"N	4°16'20.19"W
Dyad	Salt Marsh & Woodland	Seet Bridge	50°14'29.27"N	4°57'56.87"W
Dyad	Grassland & Scrub	Haytor	50°35'33.62"N	3°45'15.46"W
Dyad	Woodland & Scrub	Bodmin Moor	50°32'25.41"N	4°41'25.38"W
Dyad	Heathland & Scrub	North Hill	51°13'11.14"N	3°32'9.10"W
Dyad	Woodland & Heathland	Cloud Hill	51°12'4.17"N	3°43'44.80"W
Dyad	Grassland & Heathland	Chagford Common	50°37'30.82"N	3°51'26.36"W
Triad	Salt Marsh & Woodland & Scrub	Lychett Bay	50°43'46.89"N	2° 1'58.95"W
Triad	Sand Dune & Grassland & Scrub	Woolacombe	51° 9'46.91"N	4°12'27.04"W
Triad	Sand Dune & Grassland & Scrub	Oxwich Bay	51°33'37.95"N	4° 9'41.70"W
Triad	Salt Marsh & Heathland & Woodland	Arne	50°41'32.77"N	2° 1'43.02"W
Triad	Sand Dune & Salt Marsh & Grassland	Merthyr Mawr	51°28'20.20"N	3°38'21.78"W
Triad	Woodland & Heathland & Scrub	Woodland Hill	51° 9'18.27"N	3°12'34.34"W
Triad	Heathland & Grassland & Scrub	Hangman's Hill	51°12'48.51"N	3°58'57.27"W
Triad	Woodland & Grassland & Scrub	Dolbury Warren	51°19'29.37"N	2°46'38.81"W
Triad	Woodland & Grassland & Heathland	Bystock	50°39'16.12"N	3°22'15.55"W
Triad	Woodland & Heathland & Scrub	Haddon Hill	51° 2'49.13"N	3°28'1.46"W

Supplementary information references

1. Oksanen, J. *et al. Package 'vegan' Title Community Ecology Package Version 2.5-7*. (2020).
2. Maire, E., Grenouillet, G., Brosse, S. & Villéger, S. How many dimensions are needed to accurately assess functional diversity? A pragmatic approach for assessing the quality of functional spaces. *Glob. Ecol. Biogeogr.* (2015) doi:10.1111/geb.12299.
3. Henriksen, M. V., Chapple, D. G., Chown, S. L. & McGeoch, M. A. The effect of network size and sampling completeness in depauperate networks. *J. Anim. Ecol.* (2019) doi:10.1111/1365-2656.12912.
4. Hsieh, T. C., Ma, K. H. & Chao, A. iNEXT: an R package for rarefaction and extrapolation of species diversity (Hill numbers). *Methods Ecol. Evol.* (2016) doi:10.1111/2041-210X.12613.
5. Chao, A. & Jost, L. Coverage-based rarefaction and extrapolation: standardizing samples by completeness rather than size. *Ecology* **93**, 2533–2547 (2012).
6. Aizen, M. A., Sabatino, M. & Tylianakis, J. M. Specialization and Rarity Predict Nonrandom Loss of Interactions from Mutualist Networks. *Science* **335**, 1486–1489 (2012).
7. Morton, D. *et al. Final Report for LCM2007-the New UK Land Cover Map*. (2011).
8. Vizentin-Bugoni, J. *et al.* Influences of sampling effort on detected patterns and structuring processes of a Neotropical plant–hummingbird network. *J. Anim. Ecol.* **85**, 262–272 (2016).
9. Banašek-Richter, C., Cattin, M.-F. & Bersier, L.-F. Sampling effects and the robustness of quantitative and qualitative food-web descriptors. *J. Theor. Biol.* **226**, 23–32 (2004).
10. Rivera-Hutinel, A., Bustamante, R. O., Marín, V. H. & Medel, R. Effects of sampling completeness on the structure of plant-pollinator networks. *Ecology* (2012) doi:10.1890/11-1803.1.
