# Supplementary material for: Multi-habitat landscapes are more diverse and stable with improved function
Source: Nature. 2024 Aug 21;633(8028):114–9. doi: 10.1038/s41586-024-07825-y (PMC11374697; doi:10.1038/s41586-024-07825-y)
Supplement: Supplementary file 2 — Reporting Summary [file 41586_2024_7825_MOESM2_ESM.pdf]

nature portfolio

Corresponding author(s): Talya D. Hackett
Last updated by author(s): 13/05/2024

Reporting Summary

Nature Portfolio wishes to improve the reproducibility of the work that we publish. This form provides structure for consistency and transparency in reporting. For further information on Nature Portfolio policies, see our Editorial Policies and the Editorial Policy Checklist.

Statistics

For all statistical analyses, confirm that the following items are present in the figure legend, table legend, main text, or Methods section.

n/a	Confirmed	
	x	The exact sample size (*n*) for each experimental group/condition, given as a discrete number and unit of measurement
	x	A statement on whether measurements were taken from distinct samples or whether the same sample was measured repeatedly
	x	The statistical test(s) used AND whether they are one- or two-sided<br>*Only common tests should be described solely by name; describe more complex techniques in the Methods section.*
	x	A description of all covariates tested
	x	A description of any assumptions or corrections, such as tests of normality and adjustment for multiple comparisons
	x	A full description of the statistical parameters including central tendency (e.g. means) or other basic estimates (e.g. regression coefficient) AND variation (e.g. standard deviation) or associated estimates of uncertainty (e.g. confidence intervals)
	x	For null hypothesis testing, the test statistic (e.g. *F*, *t*, *r*) with confidence intervals, effect sizes, degrees of freedom and *P* value noted<br>*Give P values as exact values whenever suitable.*
x		For Bayesian analysis, information on the choice of priors and Markov chain Monte Carlo settings
	x	For hierarchical and complex designs, identification of the appropriate level for tests and full reporting of outcomes
x		Estimates of effect sizes (e.g. Cohen's *d*, Pearson's *r*), indicating how they were calculated

Our web collection on statistics for biologists contains articles on many of the points above.

Software and code

Policy information about availability of computer code

Data collection	No software was used for data collection. Arc GIS v.10.1 was used to initial select potential field sites before narrowing down using Google Earth 2013 and ground truth-ing.
Data analysis	All analyses were performed in the R statistical environment and all code is publicly accessible here: 10.5281/zenodo.11184586

For manuscripts utilizing custom algorithms or software that are central to the research but not yet described in published literature, software must be made available to editors and reviewers. We strongly encourage code deposition in a community repository (e.g. GitHub). See the Nature Portfolio guidelines for submitting code & software for further information.

Data

Policy information about availability of data

All manuscripts must include a data availability statement. This statement should provide the following information, where applicable:

- Accession codes, unique identifiers, or web links for publicly available datasets
- A description of any restrictions on data availability
- For clinical datasets or third party data, please ensure that the statement adheres to our policy

All data is publicly accessible here: 10.5281/zenodo.11184586

Human research participants

Policy information about studies involving human research participants and Sex and Gender in Research.

Reporting on sex and gender	N/A
Population characteristics	N/A
Recruitment	N/A
Ethics oversight	N/A

Note that full information on the approval of the study protocol must also be provided in the manuscript.

Field-specific reporting

Please select the one below that is the best fit for your research. If you are not sure, read the appropriate sections before making your selection.

☐ Life sciences ☐ Behavioural & social sciences ☒ Ecological, evolutionary & environmental sciences

For a reference copy of the document with all sections, see nature.com/documents/nr-reporting-summary-flat.pdf

Ecological, evolutionary & environmental sciences study design

All studies must disclose on these points even when the disclosure is negative.

Study description	This is empirical field based research examining community differences between landscapes with single and multiple habitats. We sampled plants, flower-visitors and herbivores (caterpillars, seed feeders and leaf miners) at 30 sites across SW England and Southern Wales consisting of 1, 2 or 3 habitats (monads, dyads and triads, respectively). Habitats were selected from a pool of six: Heathland, Woodland, Grassland, Sand Dune, Salt Marsh and Scrub.<br>We also conducted a field experiment with 20 strawberry plants placed at each site with 1 or 3 habitats to determine differences in pollination success.<br>Finally, we used the field data to model whether triads had emergent properties that were different from null triads constructed from component habitat interactions (using monad data) .
Research sample	Plants were identified and floral abundance and vegetative cover assessed in situ. Flower-visitors were sampled by hand net through 20 min of haphazard walking, euthanized with ethyl acetate and stored in a freezer before pinning or mounting for ID. Caterpillars were returned to the lab, housed individually and reared until an adult Lepidoptera or Parasitoid emerged. Insects in mined leaves, were similarly reared individually until an adult fly, moth or parasitoid emerged. Seeds likely to contain feeders were stored collectively with up to 50 seeds in a single container; all emerged adult seed feeders or parasitoids were collected individually and stored in a -20°C freezer before being pinned or mounted, where necessary.<br>Strawberries from the field experiment were picked when ripe up to 28 days from being brought to a green house (following 14 days in the field).
Sampling strategy	On each visit, we sampled along six 35 m transects. Fites were visited 4 times in total over 2 years for a total of 24 transects per site. For plants, a 0.5 m x 0.5 m gridded quadrat was placed on alternating sides of the transect every 10 m, resulting in 4 quadrats per transect. All plants within the quadrat were identified, and buds, open, wilted and seed set floral units were counted. Vegetation cover for flowering species was determined by the number of times a plant touched one of the 36 cross points formed by the intersecting grids on the quadrat.<br>Flower visitors were sampled with a hand net from within 30 m of each transect through 20 min of haphazard walking.<br>We collected leaf miners and caterpillars from 1 m2 quadrats every 10 m on either side of the transect by visual searching of leaves to a height of 2 m. They were collected and stored individually and returned to the laboratory for rearing.<br>Along each transect, we collected up to 50 seeds from plants expected to host seed feeders. Seeds were collected from within 10 m of the transect and, where possible from different plants, equally spaced along the transect. Seeds were stored separately for each plant species and returned to the lab for rearing.<br>Strawberry plants were placed in the field while in bud at the point of flowering within an 11 day period in an order that allowed 2 people in 1 car to deposit the buckets at each site across the study area within this tight time period (e.g. Sites in Wales were placed during a single return trip from Bristol, sites in Cornwall placed on a similar return trip). Strawberry plants were retrieved in the same order after they had been in the field for 14 days. Unopened flowers were removed and plants were returned to a pollinator free greenhouse for fruits to ripen.
Data collection	Field data were collected by TDH, ND, RA and field assistants (see Acknowledgments). Teams were divided such that 1 person collected floral data, another caterpillars, and a 3rd leaf miners. For flower-visitors, one person caught insects on flowers, a 2nd moved them from the net to individually labeled tubes with ethyl acetate, and the 3rd recorded the data. All data was collected by hand on field sheets or notebooks and then photocopied and digitised back in the lab. Error checking took place during digitisation, pinning, when entering IDs from taxonomists and a final time for spelling and typoes when the complete data set was compiled.
Timing and spatial scale	We sampled at 10 single habitat sites, 'monads', 10 two-habitat sites, 'dyads' and 10 three-habitat sites, 'triads'. The site size

	sampled remained constant, thus monads were a single habitat 9 ha in size, dyads consisted of two adjacent 4.5 ha habitats and triads consisted of three adjacent 3 ha habitats. All sites were surrounded by either the same habitats as those within the site, water, urban environment or farmland. Sites were spaced across SW England and Southern Wales (~250 km2). See Figure 1 for details.<br>Plants, herbivores and flower visitors were sampled from all 30 sites once May-Sept. 2014 and 3 times April-Sept. 2015 to capture the community of plants and insects through the flowering season of all habitats (e.g. woodlands in spring, grasslands in early-mid summer and heathlands towards the end of summer). Seed feeders were sampled Sept. 2014 and Aug.-Sept. 2015 when the target plants were in seed. Flower visitors were collected between 9:00 and 17:30 in dry, warm (minimum 15 ^{o}C) conditions with little to no wind.<br>Strawberry plants were placed at all monad and triad sites between 4-14 July 2015. And retrieved between 18-28 July.
Data exclusions	No data were excluded, but herbivores did not always survive until pupation; they were still included in the herbivore network. Individuals were identified from their larval stage or mine pattern and IDs confirmed by surviving adults.
Reproducibility	The sampling methods here are very close to previously published methods in Hackett et al. 2019 Ecology Letters and based on well established network ecology sampling methods (e.g. Pocock et al. 2012 Science, Baldock et al. 2019 Nature Ecology and Evolution and similar). Because sampling is field based it has not been repeated, but the detailed methods section allows the work to be repeated.
Randomization	Sites were visited in a random order in cycles such that one monad, dyad and triad was visited before a second monad, dyad or triad; the order of each site type within a cycle was also randomized; all sites were visited in a round before any site was repeated. In the 2nd year two sampling teams were operating simultaneously with separate cycles of a monad, dyad and triad. When all sites had been visited by either team (1 Round of sampling all 30 sites), the site orders were randomized in a different order and sampling continued as above.<br>At each site, on each visit, we sampled along six 35 m transects arranged as follows: six transects in the one monad habitat, three in each dyad habitat and two in each triad habitat. The transect start location and direction were randomly selected prior to arrival on site and changed on each of the four visits.
Blinding	Strawberry fruit were weighed and assigned classes blindly by an assessor with no knowledge of the originating field sites, to avoid assessment bias.<br>For all other sampling it was not possible to blind as the people sampling the field site knew where they were.

Did the study involve field work? ☒ Yes ☐ No

Field work, collection and transport

Field conditions	Floral, herbivore and seed feeder surveys were conducted in all conditions. Flower-visitor surveys between 9:00 and 17:30 in dry, warm (minimum 15 ^{o}C) conditions with little to no wind.
Location	30 sites across SW England and Southern Wales:<br>50°21'57.03"N 5° 8'51.01"W<br>51°13'23.34"N 3° 0'3.57"W<br>51°10'2.03"N 3°34'16.95"W<br>50°18'8.98"N 5°13'58.27"W<br>50°14'18.10"N 5°21'24.01"W<br>50°36'9.94"N 3°44'9.91"W<br>51°22'12.54"N 1°50'32.29"W<br>51°46'46.31"N 2°41'28.36"W<br>51°48'45.57"N 2°33'10.79"W<br>51°27'42.77"N 2°38'12.56"W<br>51° 5'25.68"N 4°12'17.30"W<br>51°19'4.91"N 2°59'8.69"W<br>50°40'28.61"N 1°56'50.10"W<br>51°40'21.03"N 4°16'20.19"W<br>50°14'29.27"N 4°57'56.87"W<br>50°35'33.62"N 3°45'15.46"W<br>50°32'25.41"N 4°41'25.38"W<br>51°13'11.14"N 3°32'9.10"W<br>51°12'4.17"N 3°43'44.80"W<br>50°37'30.82"N 3°51'26.36"W<br>50°43'46.89"N 2° 1'58.95"W<br>51° 9'46.91"N 4°12'27.04"W<br>51°33'37.95"N 4° 9'41.70"W<br>50°41'32.77"N 2° 1'43.02"W<br>51°28'20.20"N 3°38'21.78"W<br>51° 9'18.27"N 3°12'34.34"W<br>51°12'48.51"N 3°58'57.27"W<br>51°19'29.37"N 2°46'38.81"W

	50°39'16.12"N 3°22'15.55"W<br>51° 2'49.13"N 3°28'1.46"W
Access & import/export	No permits were required for us to sample the plants and insects. Access to sites was granted through verbal or written permission (typically e-mail) from all landowners which included the National Trusts, the Wildlife Trusts, Forestry England and private landowners.
Disturbance	We kept disturbance of sites to a minimum by staying on paths where possible and then moving to transect start points. using the most direct route from the path. We did not disturb organisms other than those that we sampled and this was restricted to the transect and 30 m radius around it for flower-visitors.

Reporting for specific materials, systems and methods

We require information from authors about some types of materials, experimental systems and methods used in many studies. Here, indicate whether each material, system or method listed is relevant to your study. If you are not sure if a list item applies to your research, read the appropriate section before selecting a response.

Materials & experimental systems

n/a	Involved in the study
☒	☐ Antibodies
☒	☐ Eukaryotic cell lines
☒	☐ Palaeontology and archaeology
☐	☒ Animals and other organisms
☒	☐ Clinical data
☒	☐ Dual use research of concern

Methods

n/a	Involved in the study
☒	☐ ChIP-seq
☒	☐ Flow cytometry
☒	☐ MRI-based neuroimaging

Animals and other research organisms

Policy information about studies involving animals; ARRIVE guidelines recommended for reporting animal research, and Sex and Gender in Research

Laboratory animals	No laboratory animals were used in this stuy
Wild animals	Insects were sampled with hand nets or by hand as described above, captured and killed with ethyl acetate or freezing for later identification by taxonomic experts. When flower-visitors were very numerous (e.g. pollen beetles) and morphologically similar we collected a subsample for identification and counted the excess.<br><br>A full species list of all insects caught can be found here: 10.5281/zenodo.11184586
Reporting on sex	We did not sex the insects and do not perform any analysis where sex is relevant
Field-collected samples	Strawberry plants returned from the field were housed in a pollinator-free green house, watered daily and fruit was picked when ripe for 28 days. No field-collected samples were used in this experiment.
Ethics oversight	Non-protected invertebrates (insects) only

Note that full information on the approval of the study protocol must also be provided in the manuscript.
