# Supplementary material for: Multi-habitat landscapes are more diverse and stable with improved function
Source: Nature. 2024 Aug 21;633(8028):114–9. doi: 10.1038/s41586-024-07825-y (PMC11374697; doi:10.1038/s41586-024-07825-y)
Supplement: Supplementary file 3 — Peer Review File [file 41586_2024_7825_MOESM3_ESM.pdf]

Peer Review File

Manuscript Title: Multi-habitat landscapes are more diverse and stable with improved function

Reviewer Comments & Author Rebuttals

Reviewer Reports on the Initial Version:

Referees' comments:

Referee #1 (Remarks to the Author):

I have read the manuscript by Hackett and colleagues with great interest. It is clearly an outstanding piece of work that addresses an important question: Does combining different habitat types in landscapes lead to greater stability of ecosystems formed by interacting species, as well as improved ecosystem functioning? In my opinion, the manuscript provides compelling evidence that this is indeed the case. Particularly noteworthy is the sophisticated study design, which combines a quasi-experimental selection of study landscapes (ranging from monads to dyads and triads of habitat types; distributed over a large area in the southwest of the UK) with detailed studies of several interaction types (plant-pollinator, three types of plant-herbivore interactions, and herbivore-parasitoid) and an experiment to measure pollinator function in the field. I have not seen such an ambitious study in some time (one of the few similarly ambitious studies may be that of Pocock et al. (2012, Science). In addition, the statistical methods appear to be well chosen and the conclusions based on solid results.

This being said, there are also some points for improvement. Overall, the manuscript appears to be somewhat biased in that the results on plant-pollinator interactions and pollination function are emphasized more than the results on plant-leaf miner, plant-caterpillar, plant-seed feeder, and herbivore-parasitoid interactions. One reason for this is that pollination was experimentally measured, whereas functions associated with the other plant-insect interactions (e.g., herbivory) were not directly assessed. Here, it would be interesting to hear from the authors whether similar positive effects of greater landscape heterogeneity on these functions are to be expected.

It is a great strength of the study that so many different types of species interactions were studied. As far as the word limit allows, it would be nice to discuss these different types of interactions in more detail. For example, greater landscape heterogeneity promotes the total number of species and the stability of the multilayer interaction networks (combining all interaction types) to species loss. However, it would also be interesting to determine whether certain interaction types are more stabilized by landscape heterogeneity than others (e.g., specialized plant-seed predator interactions versus more general plant-pollinator or plant-herbivore interactions).

I was wondering if the null model approach would also allow us to examine which specific combinations of habitat types lead to the greatest interaction complementarity. As I understand it, the model currently only provides evidence that (any type of) habitat combination leads to greater

complementarity than expected under the null assumption; however, it would obviously be interesting to know which specific types of combinations most effectively promote interaction complementarity and thus ecosystem functioning.

Further comments:

L31: “consistent robustness” is somewhat unclear – I think you mean “less variation in robustness”?

L32: sites = landscapes?

L44: May want to add “species” before interactions

L63: “stability” of what – network stability or stability of ecosystem functioning?

L73: With 9 ha area, the study landscapes were ca. 300 m x 300 m in size. I think the landscape size is fine for specialized species as well as herbivorous insects, but it may be rather small for mobile species, e.g., larger wild bees that forage on greater spatial scales. Can you please provide some thoughts on the size of the study landscapes, and the choice for the 9 ha area, and how this may affect your findings?

L77ff: The selection of habitat types in the dyads and triads was a bit unclear, even after reading the detailed methods. Were habitat types chosen such that all types were represented in similar numbers across dyads and triads in the end? Or were they randomly combined (which may result in some habitat types being included more often than others)?

L85ff: If possible, do not only indicate that there were differences between groups, but how the groups differed, including effect direction and strength (i.e., which group had the highest evenness, and by how much higher than the others).

L97-101: If I understand correctly, this means that you simulated primary and secondary extinctions across the whole multilayer network (combining all species interactions). This is great but did not fully become clear to me from the current wording. It became clearer after reading the detailed Methods section; however, it would be nice to highlight the novelty and strengths of your approach also in the main text (e.g. L456-461: the extension of Schleuning et al.’s approach to multipartite networks, with effects of species loss propagating through the multilayer network across different interaction types).

L102: If you simulate extinction of plants at a given site in order from rarest to greatest abundance, does this mean that the rarest species overall (across the maximum three habitat types) will go extinct first? I ask because some habitat types may have a greater proportion of rare species than others - meaning that at the beginning of the extinction cascade, (almost) exclusively these habitat types are affected by the simulated extinction. Intuitively, this would make sense (e.g., an extensively managed grassland is more likely to lose its many rare species than an intensively managed beech forest with mostly

abundant species), but it could also be one of the reasons why the results of the sequential extinction scneario differ from those based on random plant removal (I believe this is also suggested in L113).

L143-145: Given that this finding is so central to the manuscript (the “improved function” mentioned in the title), I think it also deserves a figure in the main text.

L148: As commented above - does your null model approach allow you to investigate which specific combinations of habitat types result in the greatest interaction complementarity?

L187-194: A more parsimonious hypothesis could be that the buffering effect of landscape heterogeneity is a simple portfolio effect – i.e., lower robustness in one habitat is compensated by higher robustness in another, so that with greater habitat diversity, variability in robustness is reduced. I am wondering whether the arguably more complex mechanistic explanation proposed (landscape heterogeneity affected interaction evenness, tuned by plant phylogenetic diversity) is therefore justified?

L209-215: These findings are really great. However, they seem to build mostly on the findings from the strawberry pollination experiment. I was wondering if you expect similar findings for ecosystem functions related to the plant-herbivore and herbivore-parasitoid interactions.

L340-343: Were all habitat types represented in equal proportion, without major biases in their representation (and combination) among monads, dyads and triads?

L385: How where these plants selected, e.g., by walking haphazardly across (or close to) the transects?

L398: Given the great variability in pollinator dependence for fruit set across different strawberry varieties, please also indicate the variety used in the experiment.

417: So, this would be one big, multilayered network, including all different interaction types per site?

424: Which evenness index was used (e.g., Pielou’s evenness)?

456-461: This is really great and should also be briefly mentioned in the main text.

L556: Please justify why you chose mean tree branch length as measure of phylogenetic diversity (I know there is a plethora of different indices, which are more or less complementary, it would be good why other, equally commonly used, indices, e.g., main pairwise distance or mean nearest taxon distance, were not chosen).

Figures

L287: Figure 1: Consider changing colours (e.g., red, green) to ease the interpretation for colour-blind readers.

L298: Figure 2: Would it be possible to also indicate pairwise differences of means (e.g., using a post-hoc test) directly in the figure?

L311: Figure 4: Personally, I found the results in the figure insets (panels B, D, F, H) much more important and interesting than those shown in the “main” panels (A, C, E, G). Also, the insets are very small. You may therefore consider taking the insets out of the panels – to enlarge them, and to give the more equal weight when visualizing the different results.

Best wishes,
Ingo Grass

Referee #2 (Remarks to the Author):

The authors present, by analyzing a myriad of interesting variables, a study on the effect of multi-habitat landscapes. While the reported response variables are important, and the dataset can be used to do good science, I'm afraid that either there is something critical aspect that I didn't understand, or the study presents severe problems in its conception and design. I hope I will be able to convey my concerns in a clear way.

Major Comments:

1. A problem of the natural scale. It is known that mosaic habitats are hotspots for biodiversity. Species from different habitats coexist, and certain species are specialized to these habitats. Border effects, as some of the authors definitively know, also play a huge role. The big problem is how do we define scale, and if that scale is defined by humans (9ha) or by the habitat itself (fragment size). In its current form, the manuscript comes to say that habitat fragmentation is a "good" thing, but they are not accounting for a beta-diversity effect nor for the size of the habitat fragment to which the observed habitats belong to.

In Fig. 1, I explain what I mean by the beta-diversity effect. Given two habitats White and Blue, each with 3 species, and a fragment size area of 9ha, it is expected that as fragment size decreases, the number of species in the habitat fragment will decrease. In the example below, if we reduce the habitat fragment from 9 to 4.5, then we loose one species (note that the identity of the species is different between habitats). Now, and this is the critical part. When we observe an area of 9ha, that contains both of the 4.5ha fragments of White and Blue habitats, now it turns out that we have 4 species, since, in the same—human-defined area—we have species that belong to habitat Blue and to habitat White.

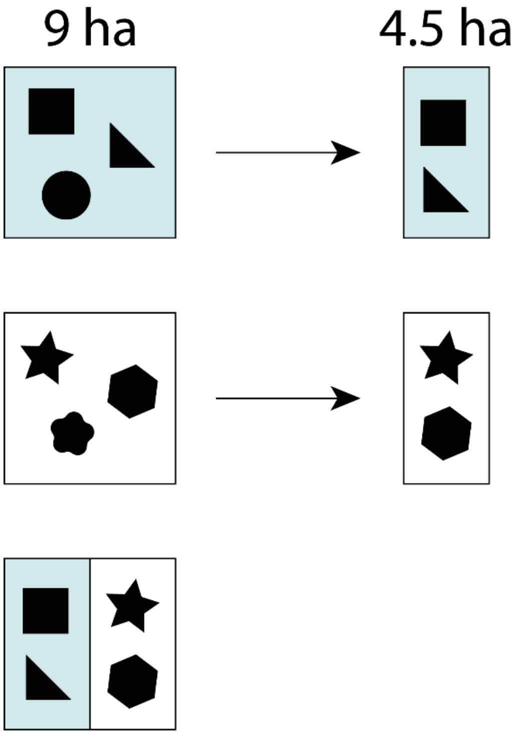

This is a major problem. Without knowing how the number of species in each of the habitats included in the study scales with the habitat fragment, it is impossible to disentangle the effects of area from the potentially emerging interactions between habitats. One would need to know how, each of the variables that the authors study, scale as a function of the true fragment size (not as a function of the studied area).

2. A problem of human scale. In the previous figure, the habitat size and the observation grid (9ah) coincide. But in reality, when we want to place the 9ha square in the map, the habitat fragments might and probably will be different. The authors must account for the area outside the sampling area. See figure 2:

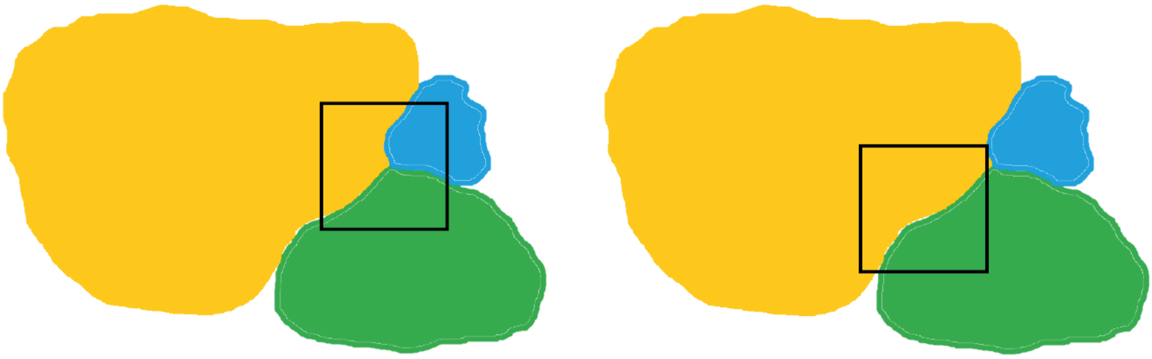

The 3 different colors represent 3 different habitat fragments of different size. The black square is the sampling area. The figure to the left is a "triad" according to the authors, and the figure to the right is a "dyad". This case, since the fragment sizes are the same, and controlled for, the sampling that the authors are doing would make sense, and one could compare across monads, dyads, and triads, always considering that the results might be an effect of the specific combination of fragment sizes shown here. Information about how all the response variables change with fragment size (fig 1) would help generalize to other combinations of fragment sizes. However, in their sampling/site designed by the authors, both things are confounded (number of habitats and fragment size of each habitat), invalidating all the results.

3. Given the limitations presented here, I find the discussion outrageous. If you consider all the unknowns, to what—if any—extent can we be sure that the results presented here are true?

4. A sliver of hope. With the data the authors have, and focusing on the monads sampling, if they manage to map (maybe using satellite images) the size of the habitat fragment where every sampling site is, and if they are lucky with the range of fragment sizes (since that is not how the study was designed), they have a very good dataset to test how all these response variables change as a function of:

- Fragment size (to explore the effects of habitat fragmentation)

- The identity of the surrounding habitats (this will come as an interaction factor with fragment size)

Other comments:

5. A The question / knowledge gap is unclear. Knowledge gaps are identified in lines 41-43, 48-51, and 58-61. The introduction needs to be restructured so that it flows better. By line 61 the reader is a bit confused by what is that the paper is about.

6. It would be nice, for non-specialists, if the authors define interaction evenness in the main text and explain why it matters.

7. Authors seem proud, and they should be, of the fact that their dataset encompasses different interaction types. However, I'm concerned that what it seems like a selling point it is a limitation in this case. It would be fantastic if the dataset would have information about all types of interactions, but it doesn't. So, while it is more complete than say, only a plant-pollinator community, is still a partial sampling of the community while it constraints the kind of stability analysis that can be done. While I see the value of the robustness analysis performed, and I understand that by combining interactions that was the only thing the authors could do, if they would have treated each interaction type separately the could have done far more interesting stability analysis. The question remains on what is there to be gained by incorporating some more interaction types without covering all of them while being constrained to that kind of robustness analysis. A comparison between extirpation experiments and say, Lyapunov stability and structural stability would be useful, albeit a standalone paper all together.

8. Strawberries. It is unclear whether strawberries where part of the the plant-pollinator network when it was sampled before the manipulative field experiment. If there is not an assessment of the potential pollinators and their preference for strawberries in each of the habitats (to know the preference one also needs to know the abundance of the other floral resources), then how do you know that, just looking at the strawberries is a good unbiassed way of measuring "pollination function"?

Line 107. What do you mean by variability in robustness? Please explain in the main text the first time it is mentioned.

Referee #3 (Remarks to the Author):

This study examined the effects of variation of landscape-scale habitat diversity on arthropod communities. The team accomplished this by designing a clever observational study that identified places in the S/SW UK landscape that, at the 9 ha scale, had either 1, 2 or 3 different “habitat” types. They then investigated in detail the structure and composition of arthropods and their food web interactions using a variety of analytical approaches, from traditional metrics of abundance, richness and evenness, to the interaction among species. They worked across four different arthropod food webs including plant-pollinator, parasite-host interactions and explored the attributes of the food web structure (as measured through functional and species-level networks) and how they varied across the different habitat-richness combinations. One of the most interesting findings of the study came from a null-model exploration of the expected network structure based on communities assembled from arthropods in landscapes with the different habitat diversity configurations. They found that the observed network configurations were non-additive and showed emergent properties that could not have been easily predicted.

The design and novelty of this study is exceptional. The number of different food webs examined, the variables explored, the experimental pollination and simulation study all contribute to a deep and exhaustive examination of the system. This is an interesting and I think unprecedented study.

Unfortunately, the details examined and discussed was also a downfall of this paper. The presentation gets completely lost in the forest for the trees, so to speak. I read through the study twice and even at the end I couldn’t easily summarize what the main findings were other that more diversity at the landscape scale changes (generally) food web structure in ways that are consistent with more general landscape-diversity hypotheses. My criticisms are primarily of the presentation of the work, not of the science or the content of the study. The methods they used were current and executed properly and the (details) of the conclusions defensible. I personally thought that the paper could have been split up into (at least) two different studies without losing the main point that was being made in this paper. In short, there is too much here, presented in too dense of a way, would strongly recommend a rewrite that simplifies the study and manuscript.

Some general suggestions for improvements:

The introductory 2 pages don't explain well enough what the drawbacks of prior studies are and why this study is such an improvement. E.g., L41-43, L47-48, L57-61
The format of the paper uses a combined results-discussion format. I think this makes the findings more difficult present and interpret. The statistical results are not elaborated upon; e.g. L92-96: no directionality given or explanation of results, L108-112: no explanation given to this statement; L137-139: what does this mean? L140-142: what does robustness to rarity effects mean?
L174-178: it is important to note that this study was entirely with insects; and the scale of analysis, 9 ha areas. I think this needs to be discussed somewhere; and how the conclusions about how this might apply to other taxa with different life-histories, dispersal ranges, etc.

L209-212, this is a well-stated key finding of the study that is buried in the paper – it appears in the last paragraph! This is what I mean by reworking the paper to highlight key statements like these and then providing the rationale for it.

Minor comments:

L50 - not clear what "with site replication" means
L160 - what is "better"

The simulation study was a nice addition. There are other studies that have taken these approaches (even with pollinators, e.g., Bennett, A.B., Gratton, C., 2013. Ecological Applications 23, 86–95. https://doi.org/10.1890/11-2029.1 that could be cited.

L200 - explain in simpler terms. More generalists means more redundancy?

Author Rebuttals to Initial Comments:

Response to Referee #1:

The manuscript appears to be somewhat biased in that the results on plant-pollinator interactions and pollination function are emphasized more than the results on plant-leaf miner, plant-caterpillar, plant-seed feeder, and herbivore-parasitoid interactions. One reason for this is that pollination was experimentally measured, whereas functions associated with the other plant-insect interactions (e.g., herbivory) were not directly assessed. Here, it would be interesting to hear from the authors whether similar positive effects of greater landscape heterogeneity on these functions are to be expected.

> *The analyses are somewhat biased towards the pollination network, simply because this is the largest network in our dataset. This is unavoidable based on the nature of the communities and logistical differences in sampling methods. While we were unable to assess pest control function experimentally, as we did with pollination function, we were able to use our biodiversity and structural data as a proxy for ecological function. We plotted the effect of number of habitats on the diversity and structural measures for each web (flower visitors, leaf miners, caterpillars and seed feeders and associated parasitoids) in the Extended Data (figures S5-S8). For these plots we combined herbivory and parasitism. We have now added two additional plots detailing the effects on herbivory and parasitism independently, but combined across the different webs (Extended Data Figure S9&10). The herbivory plots show similar, albeit weaker, overall trends as the whole network combined, particularly in species evenness, while parasitoid plots are more variable. Therefore, even though we didn't quantify herbivory or parasitism experimentally, these additional plots suggest general positive effect of multi-habitat landscapes on functions.*

It is a great strength of the study that so many different types of species interactions were studied. As far as the word limit allows, it would be nice to discuss these different types of interactions in more detail. For example, greater landscape heterogeneity promotes the total number of species and the stability of the multilayer interaction networks (combining all interaction types) to species loss. However, it would also be interesting to determine whether certain interaction types are more stabilized by landscape heterogeneity than others (e.g., specialized plant-seed predator interactions versus more general plant-pollinator or plant-herbivore interactions).

> *Different types of networks exhibit different level of interaction evenness (detailed in SI 5-10 as described above), and this is a network feature that is likely to shape the response of the corresponding community in terms of robustness (mean and variability). These differences make more uneven networks more prone to extinctions since in our robustness mode rewiring options are more limited (note, the way we hypothesise rewiring options depends on how similar one species' diet is to others'). This is likely to make the trajectory of secondary extinctions (the robustness curves) more variable since it would depend more on which plant species is removed from the network. However, robustness differences among guilds was not the focus of our work as we wanted to combine the networks to allow for shared species (larval herbivory and adult pollination). This unified approach is a strength of our work and to investigate all of the*

subcomponents of each web, habitat and landscape would be beyond the scope of this study, and also likely beyond the power of our data. Specifically, it would be circular reasoning to compare networks along a gradient of specialisation when the objective assessment of specialisation would need to be derived from the network.

I was wondering if the null model approach would also allow us to examine which specific combinations of habitat types lead to the greatest interaction complementarity. As I understand it, the model currently only provides evidence that (any type of) habitat combination leads to greater complementarity than expected under the null assumption; however, it would obviously be interesting to know which specific types of combinations most effectively promote interaction complementarity and thus ecosystem functioning.

We thank the referee for this exciting suggestion, it is a good idea and one we thought about implementing when analysing the data. Unfortunately though, it isn't possible to do this reliably with our null model of monad data, nor with our sampled dataset. One of the conclusions from our null model analysis is that the real combinations of habitats show emergent properties that are not predicted from the null model. To further this analysis, we would need multiple replicates of real habitat combinations in the field to investigate the impact of specific habitat combinations. As it stands, even with our 10 triads, we only have 1 repeated habitat combination. We would therefore need to have sampled an order of magnitude more sites before we could confidently make suggestions about which specific combination of habitats was most effective for complementarity and ecosystem function. This level of sampling simply wasn't possible for this project.

L31: "consistent robustness" is somewhat unclear – I think you mean "less variation in robustness"?

We have edited this sentence for clarity (line 31)

L32: sites = landscapes?

We have changed sites to landscapes (line 32)

L44: May want to add "species" before interactions

We have added species (line 44)

L63: "stability" of what – network stability or stability of ecosystem functioning?

We have clarified that this is the community stability (measured as robustness to species loss) in line 65

L73: With 9 ha area, the study landscapes were ca. 300 m x 300 m in size. I think the landscape size is fine for specialized species as well as herbivorous insects, but it may be rather small for mobile species,

e.g., larger wild bees that forage on greater spatial scales. Can you please provide some thoughts on the size of the study landscapes, and the choice for the 9 ha area, and how this may affect your findings?

> *There is likely to be a range of dispersal distances in pollinator communities with larger bumblebees travelling farther for foraging than smaller solitary bees and hoverflies. However, in the UK there are 24 species of bumblebees compared to c. 300 solitary bees and c. 280 hoverflies, which are far smaller. The selection of 9 ha area was partly for practicality reasons of being able to effectively sample all of our guilds (i.e., plants, herbivores and pollinators) on the site. It is a larger study plot than most previous work, both in our group and elsewhere. For example Morrison et al. (2020) sampled 4 ha sites to create hybrid networks, or 0.5 ha for moth-parasitoid networks in Hawaii (Henneman & Memmott, 2001) or 0.25 ha for host-parasitoid networks in Ecuador (Tylianakis et al. 2007). Where sampled plots are larger (e.g. Pocock et al. 2012 sampled transects in a 125 ha farm or Hackett et al. 2019 sampled smaller plots within 162 ha and 225 ha peninsular), there was no or very minimal site replication. There is necessarily a trade-off between plot size, site replication and visit replication, however the same compromise (e.g., ten 9 ha plots visited 4 times) has been made for monads, dyads and triads so there is no bias across our question. We have added text to clarify this decision in the methods section (lines 349-353).*

L77: The selection of habitat types in the dyads and triads was a bit unclear, even after reading the detailed methods. Were habitat types chosen such that all types were represented in similar numbers across dyads and triads in the end? Or were they randomly combined (which may result in some habitat types being included more often than others)?

> *We selected habitat combinations such that the same habitats were mostly equally represented in the monads, dyads and triads. That said, we could only use landscapes which were actually available in the south-west UK - thus we sampled fewer sand dunes and salt marshes across monads, dyads and triads, and more grassland and scrub. This was necessary as some habitat combinations were not found in accordance with our criteria and others consistently occur together (e.g., sand dunes and salt marshes often border grasslands). We have clarified this in lines 368-374 and all habitat combinations are in table S1 in the Extended data.*

L85: If possible, do not only indicate that there were differences between groups, but how the groups differed, including effect direction and strength (i.e., which group had the highest evenness, and by how much higher than the others).

> *We have added direction of effect as well as mean and standard deviations in the main text (lines 91-102)*

L97-101: If I understand correctly, this means that you simulated primary and secondary extinctions across the whole multilayer network (combining all species interactions). This is great but did not fully become clear to me from the current wording. It became clearer after reading the detailed Methods section; however, it would be nice to highlight the novelty and strengths of your approach also in the

main text (e.g., L456-461: the extension of Schleuning et al.'s approach to multipartite networks, with effects of species loss propagating through the multilayer network across different interaction types).

We now include this information in the main text as well (Lines 110-114).

L102: If you simulate extinction of plants at a given site in order from rarest to greatest abundance, does this mean that the rarest species overall (across the maximum three habitat types) will go extinct first? I ask because some habitat types may have a greater proportion of rare species than others – meaning that at the beginning of the extinction cascade, (almost) exclusively these habitat types are affected by the simulated extinction. Intuitively, this would make sense (e.g., an extensively managed grassland is more likely to lose its many rare species than an intensively managed beech forest with mostly abundant species), but it could also be one of the reasons why the results of the sequential extinction scenario differ from those based on random plant removal (I believe this is also suggested in L113).

Yes, when we removed species from least abundant to most abundant, rarer species are removed first across the landscape (rather than within each habitat). We have clarified this in lines 108-110. And, as you state, we indicate in lines 119-213 that an explanation for the difference in the order of removal (rare-to-common vs. random) is the distribution of rare species across the landscape.

L143-145: Given that this finding is so central to the manuscript (the "improved function" mentioned in the title), I think it also deserves a figure in the main text.

We have added this as figure 13D in the Extended Data. Adding this to our main text would be over the main text figure limit, but we are very happy to move figure 13 to the main text should the editor agree.

L148: As commented above – does your null model approach allow you to investigate which specific combinations of habitat types result in the greatest interaction complementarity?

While this is an interesting suggestion, unfortunately, as detailed above (see the question and comment at the top of pg 2), this just isn't possible with our data.

L187-194: A more parsimonious hypothesis could be that the buffering effect of landscape heterogeneity is a simple portfolio effect – i.e., lower robustness in one habitat is compensated by higher robustness in another, so that with greater habitat diversity, variability in robustness is reduced. I am wondering whether the arguably more complex mechanistic explanation proposed (landscape heterogeneity affected interaction evenness, tuned by plant phylogenetic diversity) is therefore justified?

We allude to this buffering effect, but we now add a more direct statement to this effect in line 206-208.

L209-215: These findings are really great. However, they seem to build mostly on the findings from the strawberry pollination experiment. I was wondering if you expect similar findings for ecosystem functions related to the plant-herbivore and herbivore-parasitoid interactions.

- *It is hard to predict a similarly supported functional response in herbivory and pest control without dedicated experiments. However, using a manipulative experiment MacFadyen et al. 2009 didn't find a difference in parasitism rates between organic farms (which typically have more habitat heterogeneity from increased semi-natural vegetation) and conventional ones. They did find a difference in the variability of parasitism rates, with pest control being less variable on the organic farms. See also: Gagic et al. 2012 and Paredes et al. 2022 which both examined agricultural intensification/agricultural landscape simplification effects on pest and parasitoids. However, extending this to our results is difficult due to the different contexts (e.g., agricultural vs. natural habitats and landscape management vs. number of habitats in the landscape). In short, it would certainly be interesting to investigate, but we weren't able to do so here.*

L340-343: Were all habitat types represented in equal proportion, without major biases in their representation (and combination) among monads, dyads and triads?

See our response above at the top of page 3, relating to question about L77.

L385: How where these plants selected, e.g., by walking haphazardly across (or close to) the transects?

Plants were selected haphazardly within 10 m of the transect while moving along it collecting the rest of the transect data (plant community, leaf-miners and caterpillars). This is now clarified in Lines 430-432.

L398: Given the great variability in pollinator dependence for fruit set across different strawberry varieties, please also indicate the variety used in the experiment.

They were wild native strawberries – Fragaria vesca - so there is no variety as these are not used commercially. This species was selected as they are much more robust to being left unattended in the field compared to commercial varieties and because they are perpetual flowering.

417: So, this would be one big, multilayered network, including all different interaction types per site?

Yes, this is correct.

424: Which evenness index was used (e.g., Pielou's evenness)?

Yes, we used Pielou's evenness; this is now clarified in the text in line 470

456-461: This is really great and should also be briefly mentioned in the main text.

We have added in more detail about our robustness model in the main text (lines x-xx).

L556: Please justify why you chose mean tree branch length as measure of phylogenetic diversity (I know there is a plethora of different indices, which are more or less complementary, it would be good why other, equally commonly used, indices, e.g., main pairwise distance or mean nearest taxon distance, were not chosen).

Our goal was to describe the phylogenetic diversity of each null triad and compare it to the observed communities in order to understand if interaction evenness and interaction complementarity effects were simply the outcome of higher phylogenetic diversity. We were thus interested in a metric that would describe the phylogenetic diversity of the overall community and its divergences. As suggested by Tucker et al. (2017), the mean distance/tree branch length at the community level is perfectly fit for that purpose, and different results with alternative metrics describing community phylogenetic divergence are not to be expected.

L287: Figure 1: Consider changing colours (e.g., red, green) to ease the interpretation for colour-blind readers.

Thank you for bringing this to our attention. We selected our colour palates in the other figures to be colour-blind friendly, but we somehow missed this one. We have updated this aspect of the figure to be colour-blind friendly as well.

L298: Figure 2: Would it be possible to also indicate pairwise differences of means (e.g., using a post-hoc test) directly in the figure?

While we appreciate the value of having directionality in a figure, this figure is already conveying a huge amount of information (habitat composition, data points from each site and 6 biodiversity and structural measurements across our 3 landscapes of interest). Moreover, because of the nature of MANOVA analyses, the post-hoc differences are between both number of habitats and each biodiversity measures and so are particularly complicated to illustrate in this figure. We feel adding this would make the figure busy and difficult to interpret, but we are happy to make the necessary edits if requested to do so by the editor.

L311: Figure 4: Personally, I found the results in the figure insets (panels B, D, F, H) much more important and interesting than those shown in the "main" panels (A, C, E, G). Also, the insets are very small. You may therefore consider taking the insets out of the panels – to enlarge them, and to give the more equal weight when visualizing the different results.

We now include only the previously inset null model figures here and, in response to Referee 3's suggestion to simplify the manuscript, we have moved the phylogenetic analysis figures to Extended Data Figure 17. We agree, that this does indeed make the key results of the null model easier to interpret and gives them appropriate weighting.

Response to Referee #2

1. A problem of the natural scale. It is known that mosaic habitats are hotspots for biodiversity. Species from different habitats coexist, and certain species are specialized to these habitats. Border effects, as some of the authors definitively know, also play a huge role. The big problem is how do we define scale, and if that scale is defined by humans (9ha) or by the habitat itself (fragment size). In its current form, the manuscript comes to say that habitat fragmentation is a "good" thing, but they are not accounting for a beta-diversity effect nor for the size of the habitat fragment to which the observed habitats belong to.

In Fig. 1, I explain what I mean by the beta-diversity effect. Given two habitats White and Blue, each with 3 species, and a fragment size area of 9ha, it is expected that as fragment size decreases, the number of species in the habitat fragment will decrease. In the example below, if we reduce the habitat fragment from 9 to 4.5, then we loose one species (note that the identity of the species is different between habitats). Now, and this is the critical part. When we observe an area of 9ha, that contains both of the 4.5ha fragments of White and Blue habitats, now it turns out that we have 4 species, since, in the same—human-defined area—we have species that belong to habitat Blue and to habitat White.

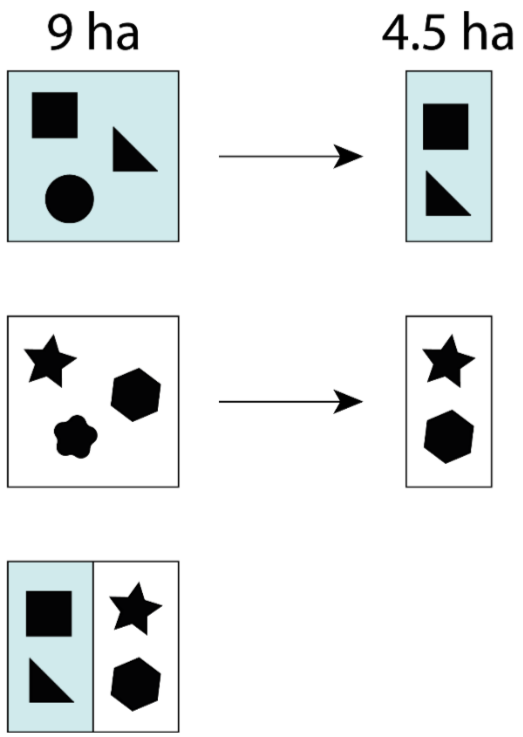

This is a major problem. Without knowing how the number of species in each of the habitats included in the study scales with the habitat fragment, it is impossible to disentangle the effects of area from the potentially emerging interactions between habitats. One would need to know how, each of the variables that the authors study, scale as a function of the true fragment size (not as a function of the studied area).

We thank the referee for raising this issue. We by no means intend to argue that habitat fragmentation is a "good" thing. Habitat fragmentation has two components: change in area of a natural habitat and change in configuration (e.g., isolation, number of fragments). We make no inferences about either of these processes, instead, we look at mosaics of naturally-occurring habitat types in different combinations.

The among-habitat beta diversity of species that the referee mentions is precisely the reason for our hypothesis that triad networks will differ from the mean of their component monads. In terms of the size of the fragment of each habitat, we based our sampling regime on previous evidence that sampling intensity is the main driver of species-area relationships, whereas the influence of patch size on per-unit-area (i.e., alpha) diversity is weak or absent (Hill et al. 1994; Schoereder et al. 2004). In other words, larger patches typically have more species in total because they contain a variety of microhabitats, each differing in species composition. Repeated sampling across these larger patches would capture a greater number of the microhabitats, and therefore reveal high between-sample (i.e., beta) diversity (Schoereder et al. 2004). Therefore, capturing these area effects on richness requires sampling effort proportional to patch size (Hill et al. 1994; Schoereder et al. 2004). Here though, we deliberately standardised the number of samples per habitat (irrespective of patch size) so that our results would not be significantly influenced by patch size. We have now added text to the methods to explain this rationale (Line 383-391).

2. A problem of human scale. In the previous figure, the habitat size and the observation grid (9ah) coincide. But in reality, when we want to place the 9ha square in the map, the habitat fragments might and probably will be different. The authors must account for the area outside the sampling area. See figure 2:

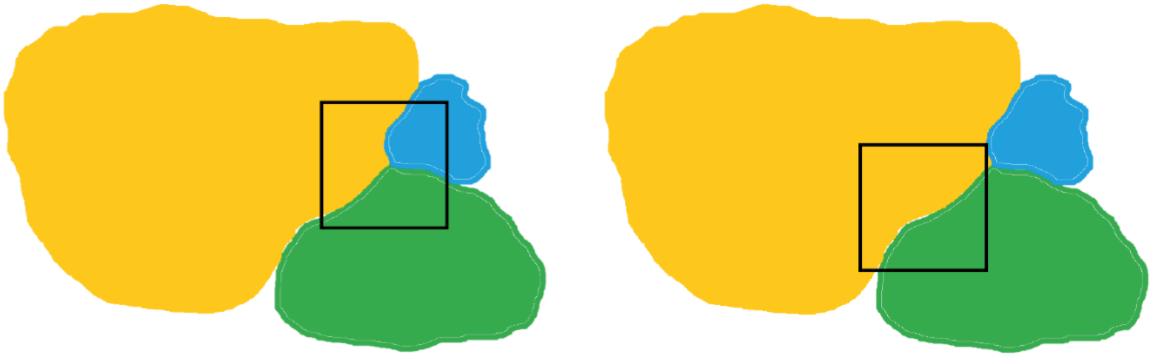

The 3 different colors represent 3 different habitat fragments of different size. The black square is the sampling area. The figure to the left is a "triad" according to the authors, and the figure to the right is a "dyad". This case, since the fragment sizes are the same, and controlled for, the sampling that the authors are doing would make sense, and one could compare across monads, dyads, and triads, always considering that the results might be an effect of the specific combination of fragment sizes shown here. Information about how all the response variables change with fragment size (fig 1) would help generalize to other combinations of fragment sizes. However, in their sampling/site designed by the authors, both things are confounded (number of habitats and fragment size of each habitat), invalidating all the results.

We thank the referee for highlighting this potential confusion in how we selected our field sites. However, the triads and dyads (and indeed monads) were not sampled as indicated in the figure here. All sites were surrounded by either urban/built-up areas, agricultural land, water, or more of the same habitats as those within the sampled area (e.g., a heathland/woodland dyad surrounded by heathland and woodland). So, our monads were single patches of a habitat, our dyads were pairs of habitat patches, and our triads were triplets of habitats - and in each case the(se) habitat(s) were surrounded by a matrix habitat of agricultural land, urban areas or water. We clarify this in lines 345-354. We therefore did not sample multiple parts of the same landscape but with our plots placed to sample specific habitat combinations in the available landscape of Southwest UK. Of course, in an ideal world, we would have all plots and surrounding area the same, but this was not possible for natural field sampling. Moreover, the focus of our work was the effect of the number of contiguous habitats rather than the surrounding landscape outside of our plots (which would have been a different research question altogether). To clarify we now include satellite images in Figure 1 that illustrate this.

3. Given the limitations presented here, I find the discussion outrageous. If you consider all the unknowns, to what—if any—extent can we be sure that the results presented here are true?

As we explain above, these two main concerns can be alleviated with additional explanation of our experimental design; our sampling methodology was designed so that our results would not be significantly influenced by patch size and our sample sites were selected to avoid the bias of an unsampled neighbouring habitat. This is recognized by the other two referees who understood that we designed our sampling protocol to test the effect of the number of habitats in the landscape on community structure, stability and function. Thus Referee 1 points out, "It is clearly an outstanding piece of work that addresses an important question…the statistical methods appear to be well chosen and the conclusions based on solid results" and referee 3 states that "The design and novelty of this study is exceptional. The number of different food webs examined, the variables explored, the experimental pollination and simulation study all contribute to a deep and exhaustive examination of the system. This is an interesting and I think

4. A sliver of hope. With the data the authors have, and focusing on the monads sampling, if they manage to map (maybe using satellite images) the size of the habitat fragment where every sampling site is, and if they are lucky with the range of fragment sizes (since that is not how the study was designed), they have a very good dataset to test how all these response variables change as a function of:

- Fragment size (to explore the effects of habitat fragmentation)

- The identity of the surrounding habitats (this will come as an interaction factor with fragment size)

unprecedented study".

While we thank the referee for suggesting interesting follow-up analyses that our data could be used for, they are very different questions and outside the intended focus of our study. As the referee points out, the data were not collected to examine the effects of fragment size or habitat fragmentation nor the effects of the surrounding habitats, which we controlled for as best we could with careful site selection. As such, we can't answer a question on fragment size effectively.

Other comments:

5. A The question / knowledge gap is unclear. Knowledge gaps are identified in lines 41-43, 48-51, and 58-61. The introduction needs to be restructured so that it flows better. By line 61 the reader is a bit confused by what is that the paper is about.

We have edited the introduction and goals in line with this and comments by Referee 3 (lines 38-74). We also now include subheadings for clarification.

Line 107. What do you mean by variability in robustness? Please explain in the main text the first time it is mentioned.

This is the variance, as measured by Brown-Forsythe's test (indicated with the statistics) of the calculated robustness at each site. We have edited this paragraph for clarity, and define robustness at the beginning of this paragraph (line 104-126).

6. It would be nice, for non-specialists, if the authors define interaction evenness in the main text and explain why it matters.

We have added an explanation of interaction evenness in line 70.

7. Authors seem proud, and they should be, of the fact that their dataset encompasses different interaction types. However, I'm concerned that what it seems like a selling point it is a limitation in this case. It would be fantastic if the dataset would have information about all types of interactions, but it doesn't. So, while it is more complete than say, only a plant-pollinator community, is still a partial sampling of the community while it constraints the kind of stability analysis that can be done. While I see the value of the robustness analysis performed, and I understand that by combining interactions that was the only thing the authors could do, if they would have treated each interaction type separately the could have done far more interesting stability analysis. The question remains on what is there to be gained by incorporating some more interaction types without covering all of them while being constrained to that kind of robustness analysis. A comparison between extirpation experiments and say, Lyapunov stability and structural stability would be useful, albeit a standalone paper all together.

In an ideal world we would have sampled all possible interactions from all taxa. However, this would be a mammoth undertaking and Referee 1 indicates "I have not seen such an ambitious study in some time". While we didn't sample all the interactions in the plots, we did sample 11,482 pairwise interactions between 954 species of insects and 324 species of plants, these being embedded within four different types of ecological network.

With regard to the robustness analysis, combining interaction types allows us to simulate cascading effects across taxonomic groups; however we retained the information about each component web (see figure 1 B, C, and D). The guilds we selected (e.g. plants, pollinators, herbivores and parasitoids) are inextricably linked both through shared plant resources, shared species due to ontogenetic dietary changes (e.g. larval herbivory to adult pollination), and

competitive interactions. Our sampling design was therefore not aimed at capturing all possible types of interactions in these communities, but rather at depicting how different guilds are interlinked in these landscapes. Robustness within each interaction type has been examined in many contexts. Doing so across multiple interaction types is rarer (but see Pocock et al. 2012); rarer still with rewiring incorporated and, to our knowledge, not at all done before for all of the above plus multiple habitats and with good site replication too! We consider our approach of simulating primary and secondary extinctions across all species interactions with rewiring and accounting for shared species in different networks based on data from 30 sites, to be a strength of our approach not a weakness. Although robustness doesn't capture all dimensions of community stability, it provides a very useful approach to understanding community response to scenarios which affects community richness (e.g., such as community simplification through landscape homogenization). The calculation of robustness is a well established method for examining spatial stability in communities of interacting species (see Baldock et al. 2019, Grass et al. 2018), and we would be unable to examine structural stability because the calculation requires data on dynamics and interaction strengths. As mentioned by the referee, comparing different metrics evaluating different dimensions of stability would indeed be interesting but is a line of research that is beyond the scope of this paper

8. Strawberries. It is unclear whether strawberries where part of the the plant-pollinator network when it was sampled before the manipulative field experiment. If there is not an assessment of the potential pollinators and their preference for strawberries in each of the habitats (to know the preference one also needs to know the abundance of the other floral resources), then how do you know that, just looking at the strawberries is a good unbiassed way of measuring "pollination function"?

We were not measuring the preference of pollinators, but rather using strawberry plants as experimental bioassays to test the effects of the surrounding habitats on pollination function (i.e. the quantity and quality of pollination). Strawberry flowers are simple open structures and are visited by a wide range of pollinators (including bees, flies and beetles) (e.g., Morrison et al. 2020). Although partially wind pollinated, as described in lines xx-xx and the caption of Figure S2, the size and quality of Fragaria vesca *fruits are improved by multiple pollinator visits (Klatt et al. 2014) and* F. vesca *occurs naturally throughout the study area (i.e., Southwest UK). As such, strawberry bioassays provide an effective approach to measure pollination function, as shown in previous studies e.g. Orford et al. 2016).*

Response to Referee #3

The presentation gets completely lost in the forest for the trees, so to speak. I read through the study twice and even at the end I couldn't easily summarize what the main findings were other that more diversity at the landscape scale changes (generally) food web structure in ways that are consistent with more general landscape-diversity hypotheses. My criticisms are primarily of the presentation of the work, not of the science or the content of the study. The methods they used were current and executed properly and the (details) of the conclusions defensible. I personally thought that the paper could have been split up into (at least) two different studies without losing the main point that was being made in this paper. In short, there is too much here, presented in too dense of a way, would strongly recommend a rewrite that simplifies the study and manuscript.

> *We thank the referee for the support regarding our content, methods and conclusions. We have edited the text throughout to clarify our goals and improve understanding. We now include subheadings that improve the readability of the paper and highlight the different aspects of our work (i.e., community structure, community stability, community function and the additive v. emergent properties of multiple habitats in the landscape). To aid in comprehension, we have focused the null model analysis results around the key findings and moved aspects of the phylogenetic diversity to Extended Data. To this end, we have edited figure 4 for clarity around the key messages and added Extended Data Figure 17. However, we prefer not to split it into multiple submissions (although we appreciate the acknowledgement of how much work went into this manuscript) as all the analyses are about the effect of the number of habitats on the structure, stability and function of the plant and insect community, and together they provide an integrated understanding of the link from landscape structure, through the structure to ecosystem function (e.g., robustness and pollination).*

The introductory 2 pages don't explain well enough what the drawbacks of prior studies are and why this study is such an improvement. E.g., L41-43, L47-48, L57-61

> *We have added additional context to the current knowledge gap (lines 38-74)*.

The format of the paper uses a combined results-discussion format. I think this makes the findings more difficult present and interpret.

> *This is the format of the journal, but we have added subheadings to clarify the key aspects of our work and edited the text for clarity.*

The statistical results are not elaborated upon; e.g. L92-96: no directionality given or explanation of results

> *We have added the direction and magnitude of effect in line with this comment and a similar one from Referee 1 (lines 89-102; 115-119; 137-142; 152-154).*

L108-112: no explanation given to this statement

We have edited this statement for clarity (lines 115-119), but we are a little unclear what is confusing the referee here as the details of the different models are provided in the methods and figure legends?

L137-139: what does this mean?

We plotted the community of pollinators in multidimensional space as a function of their dietary dissimilarity using principal coordinate analysis. We then calculated the dispersion (i.e., the mean distance to the centroid or 'multivariate average') of this cloud of species to give a dissimilarity measure for each community (interaction complementarity). We have edited the main text (lines 149-154) to clarify this and it is explained in detail in the Methods.

L140-142: what does robustness to rarity effects mean?

We were originally concerned that the results would be affected by sampling completeness and so we rarefied our dataset and re-ran this analysis. This confirmed that even when using only the very well sampled community (10+ species and first >50% and the >70% sampling completeness), the conclusions remained unchanged (i.e., sampling completeness was not an issue). This is explained in detail in the SI2. However, because it was just confirming that our results were robust to a potential bias, we prefer to leave this explanation in the Supplementary Information. We have clarified our explanation in the main text though (line 156), and hopefully the new wording "this observation is robust to the removal of rare or under-sampled species" is now clearer.

L174-178: it is important to note that this study was entirely with insects; and the scale of analysis, 9 ha areas. I think this needs to be discussed somewhere; and how the conclusions about how this might apply to other taxa with different life-histories, dispersal ranges, etc.

We have added details to the methods regarding the plot size (lines 350-354). Please see the similar comment from Referee 1 for context about the selection of 9 ha sites. Other taxa (e.g., top predators, vertebrate herbivores or any other species with a significantly larger home range) may respond to multiple habitats in the landscape differently to the guilds we selected here. But it's challenging to use our data here to predict how a large carnivore might respond to different landscapes, without some fairly rampant speculation. This is because the ecological context, resource use and life-history strategies of top predators, vertebrate herbivores etc., are very different.

L209-212, this is a well-stated key finding of the study that is buried in the paper – it appears in the last paragraph! This is what I mean by reworking the paper to highlight key statements like these and then providing the rationale for it.

We have edited the paragraph leading to this conclusion (lines 217-227) and highlighted it as a key finding in a new paragraph (lines 228-234). We hope this better frames our results and clarifies our support for this as a key conclusion bringing together multiple aspects of the paper.

L50 - not clear what "with site replication" means

We have edited this sentence for clarity (line 49-52).

L169 - what is "better"

We have edited this sentence for clarity (line 184-187).

The simulation study was a nice addition. There are other studies that have taken these approaches (even with pollinators, e.g., Bennett, A.B., Gratton, C., 2013. Ecological Applications 23, 86–95. https://doi.org/10.1890/11-2029.1 that could be cited.

We have added the suggested reference to the methods section (line 550).

L200 – explain in simpler terms. More generalists means more redundancy?

More generalism in terms of species interactions is a possible explanation for sampled triads having lower complementarity – as with increased generalism, diet overlap is more likely – than null triads (where the same number of interactions were randomly sampled from monad communities). However, the sampled triads had higher complementarity than the actual sampled monads. Thus, triads show both real world differences from monads (increased interaction evenness and complementarity), plus there is the emergent property from real habitat combinations (increased interaction evenness and increased redundancy). Moreover, the experimental results from the strawberry bioassay indicate improved pollination function. We have edited this sentence and the paragraph in general for clarity (lines 217-227)

References:

Baldock, K. C. R. *et al.* A systems approach reveals urban pollinator hotspots and conservation opportunities. *Nature Ecology & Evolution* 1 (2019).

Bascompte, J., Jordano, P., Olesen J. M. Asymmetric Coevolutionary Networks Facilitate Biodiversity Maintenance. Science 312, 431 (2006).

Gagic, V. et al. Agricultural intensification and cereal aphid–parasitoid–hyperparasitoid food webs: network complexity, temporal variability and parasitism rates. Oecologia 170, 1099–1109 (2012).

Grass, I., Jauker, B., Steffan-Dewenter, I., Tscharntke, T. & Jauker, F. Past and potential future effects of habitat fragmentation on structure and stability of plant–pollinator and host–parasitoid networks. *Nature Ecology and Evolution* **2**, 1408–1417 (2018).

Hill, J. L., Curran, P. J. & Foody, G. M. The Effect of Sampling on the Species-Area Curve. *Global Ecology and Biogeography Letters* **4**, 97–106 (1994).

Klatt, B. K. *et al.* Bee pollination improves crop quality, shelf life and commercial value. *Proceedings of the Royal Society B: Biological Sciences* **281**, 20132440 (2014).

Orford, K. A., Murray, P. J., Vaughan, I. P. & Memmott, J. Modest enhancements to conventional grassland diversity improve the provision of pollination services. *Journal of Applied Ecology* **53**, 906–915 (2016).

Macfadyen, S. *et al.* Do differences in food web structure between organic and conventional farms affect the ecosystem service of pest control? *Ecology Letters* **12**, 229–238 (2009).

Paredes, D., Rosenheim, J. A. & Karp, D. S. The causes and consequences of pest population variability in agricultural landscapes. Ecological Applications 32, e2607 (2022).

Schoereder, J. H. *et al.* Should we use proportional sampling for species–area studies? *Journal of Biogeography* **31**, 1219–1226 (2004).

Tucker, Caroline M., Marc W. Cadotte, Silvia B. Carvalho, T. Jonathan Davies, Simon Ferrier, Susanne A. Fritz, Rich Grenyer, et al. 'A Guide to Phylogenetic Metrics for Conservation, Community Ecology and Macroecology'. *Biological Reviews of the Cambridge Philosophical Society* 92, no. 2 (2017): 698–715.

Tylianakis, J.M, Tscharntke, T. & Lewis, O.T. Habitat modification alters the structure of tropical host–parasitoid food webs. *Nature* **445,** 202-205 (2007).

Reviewer Reports on the First Revision:

Referees' comments:

Referee #1 (Remarks to the Author):

The authors addressed many of the questions, concerns and suggestions that were raised, and I am on overall happy with the way the authors have addressed my comments.

Amongst others, the changes include a revised introduction; more information on study site selection and null model analysis; results now include effect strengths and directions; figures have been revised for greater clarity; and the interpretation of results has become much clearer. This is an excellent paper and I congratulate the authors on their work!

Regarding the statistics, I could not find any problems, and the description of error bars and probability values appears to be fine throughout the manuscript (some very minor details should be added, see comments below).

A few minor points remain:

L165: „plant-pollinator“ instead of „plants-pollinator“

L179-181: I do not understand the lower case numbers behind the „r“ of the correlation coefficients (e.g. r9969))

L212: Plant phylogenetic diversity is not part of the revised Figure 4 anymore - so this should probably not refer to Fig 4A&E but the new Extended Data Figure 17.

L323ff, Figure 2: I suggest to to include letters indicating statistically significant pairwise differences between monads, dyads, triads (you may simply extent the y-axis of each panel and then plot the letters above the whiskers)

L329: Please also include a short explanation of the colored dots in panel A.

L334: Include explanation of the stars code (e.g., *** = $p < 0.001$)

Best wishes,
Ingo Grass

Referee #2 (Remarks to the Author):

After carefully reading the revised version of the manuscript, supporting information, extended data, and the authors point-by-point reply, I still have concerns about the relevance of the results (if those results hold after further scrutiny), but more importantly about whether the results hold in light of the sampling design and the variables the authors are controlling for. I recognize the huge effort that went into a sampling campaign this big, and the combination with experiments and theory, but that on its own should not be enough. I hope that I can articulate my concerns in a constructive way.

Major comments:

1. In the methods section, the authors mention: “We designed our data collection under the assumption that sampling intensity is the main driver of species-area relationship, while the influence of patch size on per-unit-area (i.e. alpha) diversity is weak or absent”. And they go on to say that “Thus, larger patches typically have more species in total because they contain a variety of microhabitats”. While I agree that when comparing two communities sampled at different efforts, the effect of the sampling will be much greater than almost anything else, this doesn’t mean that habitat size should be ignored. And standardizing the number of samples per site, as the authors did, will not avoid patch size bias, as the authors claim, at a minimum, that should be demonstrated. Let me divide this first major point into two for the sake of clarity.

1.1. Rarefaction curves:

I thank the authors for showing the rarefaction curves for the number of interactions. My concern is that while the use of Chao’s estimator is a fantastic tool to compare levels of biodiversity at equal completeness, I’m not sure that the same can be applied to then infer network properties. If the idea would be just to count the number of interactions and the number of species, to say compare connectance, it would be ok. However, since the idea is to talk about network properties and calculate robustness, it is not the number of species or the number of interactions what matter, but how they are arranged.

It would be necessary to know how the properties that the authors are interested in scale with sampling effort. A sensitivity analysis is a must if we want the results to be credible. We need to know how robustness, evenness, and the reported difference between monads and triads scale with completeness.

This is of particular importance given the fact that the rarefaction curves reported for the number of interactions show that most communities were sampled nowhere near the saturation point.

1.2. Effect of patch size:

Some of the authors themselves have been the ones proving the effect of patch size not only for

number of species (as shown countless other times), but for interactions and community properties in plant-pollinator networks (Aizen, Sabatino, Tylianakis, Science 2012). We can hold onto this paper because I think it will be relevant to discuss some of the results of the current study, but I just wanted to point it out because the effect of fragment size, even if for whatever reason doesn't exist in the studied area, since it is known to be prevalent everywhere else, it needs to be ruled out, or it should be included in the statistical models. If it doesn't affect the results, it will be great, but it needs to be demonstrated, not just argued away.

What I would do is the following:
- Get the size of the habitat fragment where the monads are by using satellite images. 9ha were sampled in a forest, let's say, but how big was that forest fragment.
- Then test, for each habitat type (or including habitat type as a categorical factor), the relationship between fragment size and species richness, number of interactions, robustness, and evenness.
- If the size is shown to matter, then it should be included in the statistical model. For that, the habitat fragments that form the dyads and the triads should also be tracked. One should see if the range of those habitat fragment fall in the range of the habitat fragments from the monads (that would be the best). Otherwise, the assumption that the same relationship holds for out-of-sample sizes should be mentioned.
- This opens also the opportunity to explore interactions between fragment size and the monad-dyad-triad continuum.

If after this the results still hold, then great. We can move on with the next major point.

(Another way to have controlled by size would have been to sample the monads inside adjacent fragments, and then the dyads and the triads at the interface between those same fragments, but it is too late for that now).

(I'm not going to comment on the effect of isolation, as it is more difficult to quantify for complex communities)

2. My next concern is about the relevance of the results themselves.

- Rare species and specialist species are more likely to go extinct first. If there is a species-habitat specialization, and in the dyads and triads a smaller area of the habitat is sampled, it is expected that those rare and specialist species will tend to show in fewer numbers in dyads and triads. If that is not the case, it needs to be shown. If the specialist rare species are not shown in the sampling, when calculating robustness, dyads and triads are going to be more robust than monads, since the species that would go extinct first (specialist and rare species) have not been found. This may create the illusion of emergent properties (always exciting to find) that cannot be explained by the combination of monads found in different habitats. At this point, I'm afraid that we don't know if those emergent properties are really there or if this is an effect of the sampling as mentioned in this comment. The authors should have the

information to test this.

Also, regarding emergent properties, if they are really there, why (looking at Fig. 4) sometimes are higher and sometimes lower than the 1-to-1 line? Why they wouldn't be consistent in their departure from the null model?

- What is the biological meaning of a reduction in the variability in robustness between monads, dyads and triads? How do you know that, as mentioned before, this reduction in variability is not due to the lack of specialist and rare species in the dyads and triads due to the fact that a smaller area was sampled in each constituent habitat?

A test would be to see if there is any relationship, for each habitat type, between the average degree of the species found in 9ha in a monad, 4.5ha in a dyad, and 3ha in a triad. If the average degree does not decline with sampled habitat, then we can maybe talk about emergent properties. If the average degree declines with sampled habitat, then both effects are likely to be confounded.

- According to the results, the higher the number of habitats in a given area, the better. Therefore, mosaics are better than continuous habitat, and fragmentation is a good thing. Given the aforementioned shortcomings, I think it is a dangerous message.

Minor comments:

Title: "more diverse and stable". The results do not show an increase in stability, but a decrease in the variance of the quantified robustness, therefore the title is not aligned with the results.

Lines 154-156: This finding is compatible with my major comment 2.2, a group of generalist species will have more complementary diets, which might be a true effect, but also a sampling effect. Also, it might be influenced by edge effects, if certain specialist species need of larger continuous habitats specific habitats.

Lines 215-216: Authors put in a positive light the multy-habitat effect, but if that increase in robustness is due to a reduction on the number of specialists species, we will have more robust communities only at the cost of loosing certain species. Not only that, but we could anticipate which species are going to be lost due to the relationship between area (fragmentation) and network structure and composition (Aizen, Sabatino, Tylianakis, Science 2012).

Line 233: The results do not support the claim of a greater stability.

Lines 593-595: "if the boxplot overlaps…" This should be copy-pasted as it is into the figure caption. It is a much clearer explanation.

Extended data, figure 14. In the figure caption it says “number of pollination interactions”, but the x-axis says “number of individuals”. It is unclear what is the variable in the x-axis. It is easier if it says “number of individuals sampled” in the x-axis and Interaction sample coverage in the y-axis.

Figure 1. It seems that the networks do not form a giant component, which in combination with the reported rarefaction curves, shows that the networks were not fully sampled.

Referee #3 (Remarks to the Author):

Thank you for a much improved version of this manuscript. I think it is much easier now to understand the main take home messages and to recognize the value of this interesting study. Well done.

In my opinion, there is still a lot to be done to better present the Results/Discussion. Although this section is nominally a combined section, it is really presented as a Results first (L87-184), Discussion later (L185-235). I think that given the more succinct version the manuscript and story would benefit from a more integrated version. For example, I think the presentation of the main empirical findings of the study (e.g., L89-93) beg for an immediate discussion of whether these patterns are novel/interesting/supported by other studies or theory, and how to interpret these studies. Some of these points are made later on in what I have labeled the “discussion”, so it wouldn’t be much work to weave these two sections together. There could be a then a “Conclusion” section that would allow you to broaden out and talk about the caveats to the study (there are many and I don’t think you have adequately addressed some of them in the initial responses) and what this could mean for conservation, landscape planning, and the theories around ecosystem functioning and the provisioning of ecosystem services. This last part is touched on here and there and generally is not very well organized. Given the broad audience for this journal, it is reasonable to address these broader themes.

Specific comments, suggestions (Lines):

45, delete apostrophe in “species’ “
73-75, suggest “habitats affects one ecological function—pollination of a plant by insects. Finally, we use a modelling approach to investigate if landscape-scale networks have emergent properties that cannot be explained simply by summing their component habitat networks.
84, this would be a good place to make some of the points from the Methods and as brought up by reviewers about the experimental design and selection of sites
90-94 Example here of a good place to explain the interpretation of these results. Then you can use the narrative lower down to talk about the meaning and relevance of this finding
94 “increases” … of what?
150 change to “differences”
156-57, suggest “thus sites with a pollinator community with more dispersed diets is expected to have

higher interaction complementarity"
160, suggest "... , they hosted sets …"
191, this would be a good place, again, to clarify what this finding means

I would suggest adding a "Conclusion" section at L193 as described above

Author Rebuttals to First Revision:

Response to Referee #1:

The comments from Referee 1 were relatively minor and our responses are as follows:

L165: “plant-pollinator“ instead of “plants-pollinator“

This is now fixed. (Line 165)

L179-181: “I do not understand the lower case numbers behind the „r“ of the correlation coefficients (e.g. r9969))”

We did a repeated measures correlation test to account for multiple null model values based on the data from each site (Bakdash and Marusich, 2017). The subscripts are the degrees of freedom. We have edited the text and methods to clarify. (Line 179-182)

L212: “Plant phylogenetic diversity is not part of the revised Figure 4 anymore - so this should probably not refer to Fig 4A&E but the new Extended Data Figure 17”.

This is now corrected. (Line 213)

L323ff, “Figure 2: I suggest to include letters indicating statistically significant pairwise differences between monads, dyads, triads (you may simply extent the y-axis of each panel and then plot the letters above the whiskers)”

Because of the nature of MANOVA analyses, the post-hoc differences are between both number of habitats and each biodiversity measure. Thus, the significant pair-wise differences are both within and among each panel (e.g., there are differences in all measures together between monads and triads, and species evenness differences contributing to the overall MANOVA output). The full output is in Extended Data Supplementary Table S2.

L329: “Please also include a short explanation of the colored dots in panel A.”

We have added an explanation of the individual data points in the caption of Fig. 3. (Line 332-333)

L334: “Include explanation of the stars code (e.g., *** = p < 0.001)”

We have clarified the asterisk meanings in the caption to Fig. 3. (Line 337)

Response to Referee #2:

1.1. The correlation between sampling completeness and robustness/ interaction evenness

"I thank the authors for showing the rarefaction curves for the number of interactions. My concern is that while the use of Chao's estimator is a fantastic tool to compare levels of biodiversity at equal completeness, I'm not sure that the same can be applied to then infer network properties. If the idea would be just to count the number of interactions and the number of species, to say compare connectance, it would be ok. However, since the idea is to talk about network properties and calculate robustness, it is not the number of species or the number of interactions what matter, but how they are arranged. It would be necessary to know how the properties that the authors are interested in scale with sampling effort. A sensitivity analysis is a must if we want the results to be credible. We need to know how robustness, evenness, and the reported difference between monads and triads scale with completeness. This is of particular importance given the fact that the rarefaction curves reported for the number of interactions show that most communities were sampled nowhere near the saturation point".

The effect of both species and interaction sampling completeness are important considerations in network ecology. Therefore, our study design ensured that sampling effort was equal across all sites, such that differences in abundances and frequencies would represent real differences. However, controlling for sampling effort does not prevent differences in sampling completeness associated with specific within-site habitats (Figure 1 on the following page).

One way in which we addressed this problem was by using quantitative network metrics because, as demonstrated by Vizentin-Bugoni et al. (2015): "quantitative metrics, such as interaction evenness, specialization (H2'), weighted nestedness (wNODF) and modularity (Q; QuanBiMo algorithm) were less biased by sampling incompleteness than binary metrics.". Banašek-Richter et al. (2004) and Henriksen et al. (2018) also found quantitative metrics to be less sensitive to sampling. Moreover, Rivera-Hutinel et al. (2012) demonstrated that "nestedness, modularity, and robustness to species loss were less influenced by insufficient sampling than connectance, path length, and centralization". Therefore, in addition to standardised sampling effort, the measures in question have been demonstrated to be less sensitive to sampling effort than many network measures.

Nevertheless, to investigate if sampling completeness was significantly contributing to our results, we plotted both robustness and interaction evenness against interaction sampling completenesss, with colour-coded datapoints for monads, dyads and triads. There is no relationship between sampling completeness and robustness and this relationship does not change between site types. For interaction evenness, we ran a repeated measures correlation analysis testing for a correlation between interaction evenness and sampling completeness across replicated monads, dyads and triads. This also showed no significant correlation (r_{26} = -0.257; p = 0.187 ; Figure 2 on the following page).

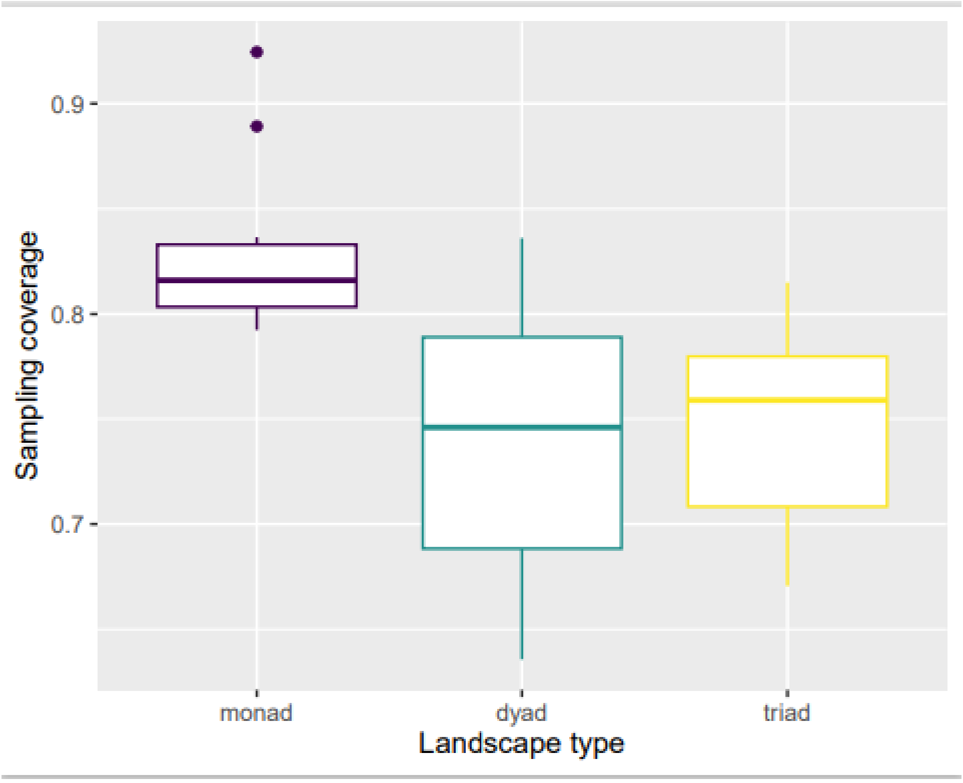

Figure 1: Boxplots of the interaction sampling completeness (or sampling coverage) for monads, dyads and triads. Monads do indeed have higher interaction sampling completeness than dyads and triads.

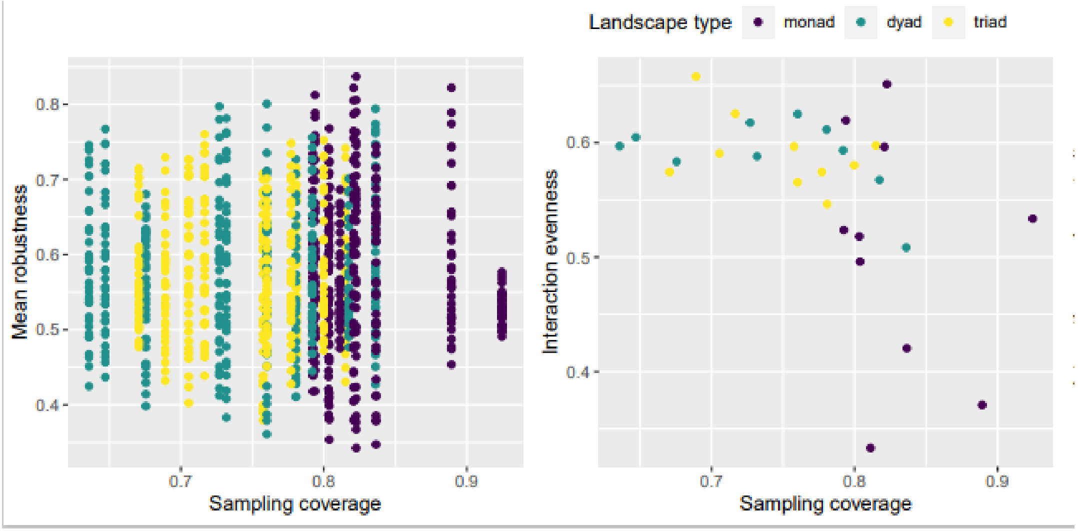

Figure 2: Robustness calculations of all extinction and threshold scenarios (left) for each monad (purple), dyad (teal) and triad (yellow) plotted against interaction sampling coverage. Robustness

does not correlate with differences in sampling completeness. Interaction evenness (right) for each monad, dyad and triad plotted against sampling completeness. A repeated measures correlation analysis shows that this correlation is also not significant.

1.2. The effect of patch size:

"Some of the authors themselves have been the ones proving the effect of patch size not only for number of species (as shown countless other times), but for interactions and community properties in plant-pollinator networks (Aizen, Sabatino, Tylianakis, Science 2012). We can hold onto this paper because I think it will be relevant to discuss some of the results of the current study, but I just wanted to point it out because the effect of fragment size, even if for whatever reason doesn't exist in the studied area, since it is known to be prevalent everywhere else, it needs to be ruled out, or it should be included in the statistical models. If it doesn't affect the results, it will be great, but it needs to be demonstrated, not just argued away."

As requested in the referee's instructions (see Referee Comments), we investigated a potential effect of habitat patch size on our results. To this end, for all monads we calculated the contiguous patch size of the surrounding habitat (grassland, heathland, salt marsh, sand dune, scrub, and woodland) on ArcGIS v.10.8. We used the Land Cover 2007 data for 7 sites (confirmed against ground truthing, photos and site maps from the time of sampling). For the remaining 3 sites ("Wyeswood Common", "Pewsey Down" and "Dartmoor Scrub"), the Land Cover data did not match our ground truthing at the time and was calculated using a combination of photos, site maps, Google Earth data and details from Natural England or the Gwent Wildlife Trust. We then plotted the surrounding patch size against our six structural measures included in the MANOVA, indicating the habitat (see Figure 3 below). Accounting for habitat means that the power of any correlation analysis would be weak, but there is no discernible effect of patch size on any metric.

We have added this analysis and figure to Supplementary Information Section 4.

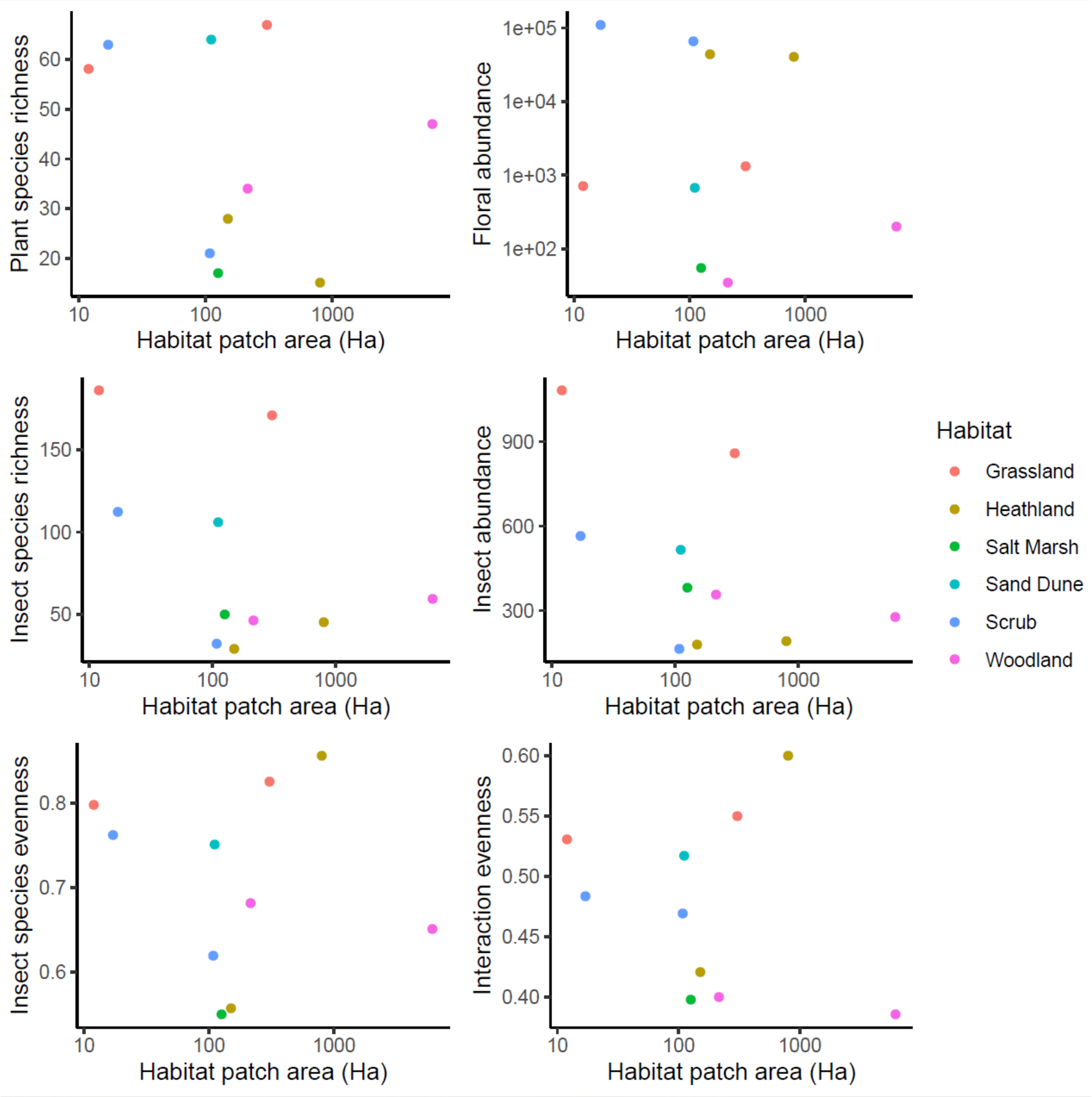

Figure 3: Surrounding habitat patch area of all monad sites plotted against the six structural measures (plant species richness, floral abundance, insect species richness, insect abundance, insect species richness, and interaction evenness) from our MANOVA. The habitat (grassland, heathland, salt marsh, sand dune, scrub and woodland) is indicated by the colour of the point. There is no trend in any of the metrics.

3: The effect of landscape type (monad, dyad & triad) on the presence of singletons.

"Rare species and specialist species are more likely to go extinct first. If there is a species-habitat specialization, and in the dyads and triads a smaller area of the habitat is sampled, it is expected that

those rare and specialist species will tend to show in fewer numbers in dyads and triads. If that is not the case, it needs to be shown".

To investigate if there are in fact more rare species sampled at monads than dyads than triads, we extracted both the number and proportion of singletons (species sampled only once at the local site level) recorded at each site across all webs. As can be seen in Figure 4 below, neither the number nor the proportion changed across treatment.

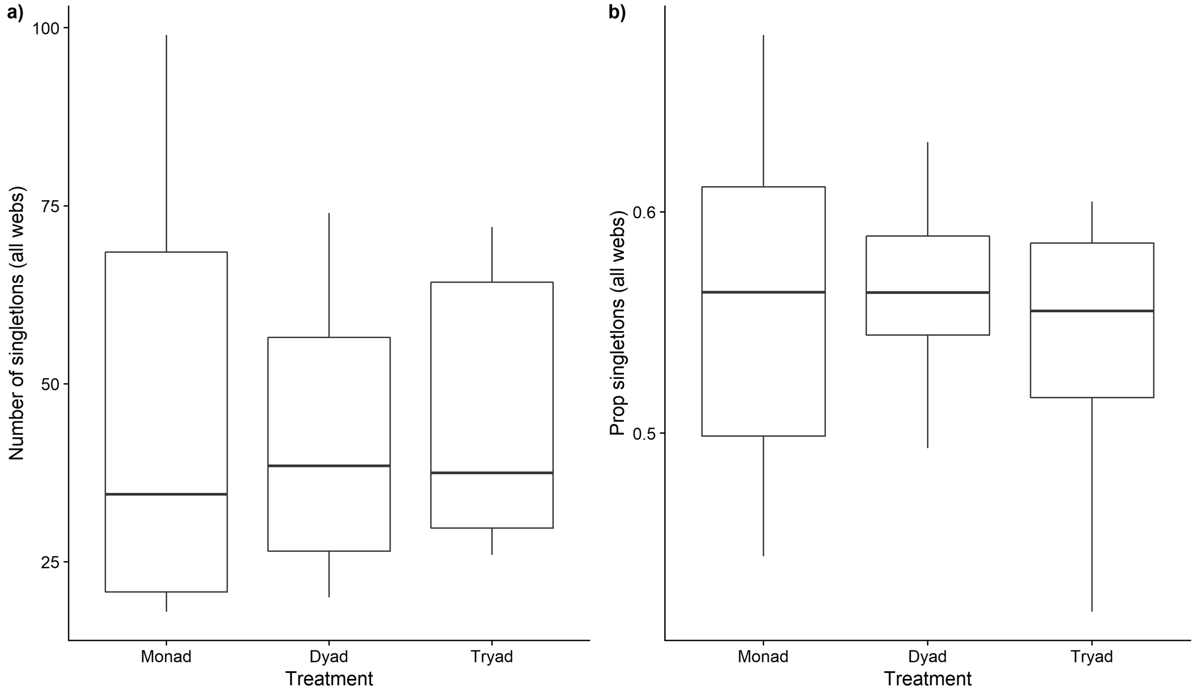

Figure 4: The number (left) and proportion (right) of singletons (species recorded only once at their site) for monads, dyads and triads. There is no effect of treatment on our sampling of rare species.

4: Emergent properties, including robustness (it's biological meaning and the impact of specialists)

"Also, regarding emergent properties, if they are really there, why (looking at Fig. 4) sometimes are higher and sometimes lower than the 1-to-1 line? Why they wouldn't be consistent in their departure from the null model?"

Each boxplot represents the null model from habitat combinations on one of our sites. For example, most sites (7 out of 10) show higher interaction evenness than their respective null model, but this is not true for all sites/habitat combinations (2 are equivalent to null and 1 shows slightly less). This was sufficiently consistent to be statistically significant, but there is variation among sites in the extent to which they deviate from null – such variation is the norm in

biological systems. Our intent was to test specific hypotheses, rather than to attempt to explain all variance in our dataset, and attempting to explain environmental noise would simply be conjecture. The variation amongst sites might be due to the specific combination of habitats (e.g., the two woodland/heathland/scrub sites deviate most from null), but we would need a much larger number of sites with many replicates of every habitat combination to test for this. All we can confidently say with these data is that most sites/habitat combinations are showing this emergent property in the same direction. Our other more constrained null models start to get closer to the contributing aspects (e.g., plant species richness or phylogenetic diversity), but none fully explain the effect and the emergent properties remain, although weakened, in most instances. We clarified this in the Fig. 4 caption (Lines 344-346) and discuss it in Lines 169-184. It would be a really interesting follow-on question to investigate the specific factors that are driving this trend in most of our sites (but not all); and collecting data on replicate habitat combinations would be a good first step. However, it is, unfortunately, beyond the scope of our current data from 30 independent field sites.

"What is the biological meaning of a reduction in the variability in robustness between monads, dyads and triads? How do you know that, as mentioned before, this reduction in variability is not due to the lack of specialist and rare species in the dyads and triads due to the fact that a smaller area was sampled in each constituent habitat? A test would be to see if there is any relationship, for each habitat type, between the average degree of the species found in 9ha in a monad, 4.5ha in a dyad, and 3ha in a triad. If the average degree does not decline with sampled habitat, then we can maybe talk about emergent properties. If the average degree declines with sampled habitat, then both effects are likely to be confounded"

The biological meaning of the reduction in variability in robustness is stated in Lines 203-209:

"The higher variability in single-habitat landscapes means that extremes of robustness are more common, putting communities at individual sites more frequently at risk of cascading effects of species loss. At the landscape-scale, the benefits of multi-habitat configurations therefore allow for a buffering effect, even if there is no average loss of robustness in landscapes with fewer habitats; more robust habitats might compensate for lower robustness in contiguous habitats resulting in a landscape-level decrease in robustness variability".

As shown above (cf., Figure 3: The effect of landscape type (monad, dyad & triad) on the presence of singletons), rare species are not more likely to be sampled in monads than dyads or triads, thus we can be confident that this is not a confounding effect.

Minor comments:

“According to the results, the higher the number of habitats in a given area, the better. Therefore, mosaics are better than continuous habitat, and fragmentation is a good thing. Given the aforementioned shortcomings, I think it is a dangerous message”.

First, there is substantial evidence that habitat diversity promotes various measures of biodiversity, essentially because beta diversity increases as alpha diversity decreases (e.g., Kerr et al 2001; Hendrickx et al 2007; Pacheco et al. 2012). This is what Tscharntke et al. (2012) termed ‘The dominance of beta diversity hypothesis’: “The landscape-moderated dissimilarity of local communities determines landscape-wide biodiversity and overrides negative local effects of habitat fragmentation on biodiversity.” Also relevant to our manuscript is their ‘Landscape-moderated insurance hypothesis’: “Landscape complexity provides spatial and temporal insurance, i.e., higher resilience and stability of ecological processes in changing environments.” (see also many references therein). The referee’s perspective is thus at odds with the landscape ecology literature, which has increasingly recognised the role of beta diversity in driving counterintuitive relationships between landscape structure and simple measures of biodiversity like species richness.

Second, we are not suggesting that fragmentation is a good thing, but rather that our data and analyses support the conclusion that habitat diversity is important across the landscape and maintaining diverse mosaics where they exist could be an important aspect of landscape management and conservation. We have addressed the above comments and have edited the text to clarify this point (Lines 193-200)

We note also that our habitats are largely natural (Most of our study sites were in fact national parks or protected areas), whereas habitat fragmentation would typically involve an increased proportion of disturbed habitats, which typically have lower species turnover (i.e., the same set of recurring generalists). It is therefore not appropriate to draw a parallel between diverse natural habitats and land-use change. If our study landscapes were homogenized to a single habitat type, there would absolutely be a loss of diversity.

“Title: “more diverse and stable”. The results do not show an increase in stability, but a decrease in the variance of the quantified robustness, therefore the title is not aligned with the results”.

Decreasing variability is a measure of stability (Ives & Carpenter 2007), so we are somewhat perplexed by this comment.

*Nevertheless, we have changed the title to “**Multi-habitat landscapes support diversity, stability and improved function".** (Line 1)*

“Lines 154-156: This finding is compatible with my major comment 2.2, a group of generalist species will have more complementary diets, which might be a true effect, but also a sampling effect. Also, it might be influenced by edge effects, if certain specialist species need of larger continuous habitats specific habitats”.

This finding holds true though, with just the core of the community - species with 10+ individuals sampled and both 50% and 70% sampling completeness (as outlined in Supplementary Information Section 2). Moreover, as demonstrated above, there is no evidence of an effect of treatment on the detection of rare or specialist species. And while some specialist species might rely on large continuous habitats, others might also rely on multiple contiguous habitats as outlined in Lines 196-200.

"Lines 215-216: Authors put in a positive light the multi-habitat effect, but if that increase in robustness is due to a reduction on the number of specialists species, we will have more robust communities only at the cost of losing certain species. Not only that, but we could anticipate which species are going to be lost due to the relationship between area (fragmentation) and network structure and composition (Aizen, Sabatino, Tylianakis, Science 2012)".

As indicated above, we did not find any effect of either size of surrounding habitat patch or detection of rare or specialist species. A key difference from the Aizen et al (2012) system is that their sierras were the only diverse habitat within a matrix of highly-intensive agriculture (essentially functioning as islands). In contrast, our landscapes contain a diversity of natural habitats (sand dunes, salt marshes, heathland, woodland, grassland, scrub), so we would not expect systematic changes in the number of specialist species.

Line 233: "The results do not support the claim of a greater stability".

We have edited this text accordingly (Line 235).

Lines 593-595: "if the boxplot overlaps…" This should be copy-pasted as it is into the figure caption. It is a much clearer explanation.

We have edited the figure caption as suggested (Lines 344-346).

"Extended data, figure 14. In the figure caption it says "number of pollination interactions", but the x-axis says "number of individuals". It is unclear what is the variable in the x-axis. It is easier if it says "number of individuals sampled" in the x-axis and Interaction sample coverage in the y-axis".

We clarify in the caption that the number of interaction events is a measure of the number of individuals, since individuals were not sampled except when engaging in an interaction (to ensure that they could be associated with a specific network). Thus, we did not have an independent measure of individuals other than those recorded interacting, and we have edited the caption to clarify this distinction.

"Figure 1. It seems that the networks do not form a giant component, which in combination with the reported rarefaction curves, shows that the networks were not fully sampled".

The aim of Figure 1 is to clearly demonstrate how contiguous habitats are connected by shared species using multiple habitats, a point often missed when sampling within habitats and/or interaction types. In reality all published networks are samples of their communities and as such are not "fully sampled". Here we have presented data collected from each of our 30 sites, gathered by a very careful and consistent sampling effort over a two-year period, and in reference to the new analyses outlined above, unconfounded by sampling completeness, patch size or variation in the numbers of rare species.

Response to Referee #3:

In my opinion, there is still a lot to be done to better present the Results/Discussion. Although this section is nominally a combined section, it is really presented as a Results first (L87-184), Discussion later (L185-235). I think that given the more succinct version the manuscript and story would benefit from a more integrated version. For example, I think the presentation of the main empirical findings of the study (e.g., L89-93) beg for an immediate discussion of whether these patterns are novel/interesting/supported by other studies or theory, and how to interpret these studies. Some of these points are made later on in what I have labelled the "discussion", so it wouldn't be much work to weave these two sections together. There could be a then a "Conclusion" section that would allow you to broaden out and talk about the caveats to the study (there are many and I don't think you have adequately addressed some of them in the initial responses) and what this could mean for conservation, landscape planning, and the theories around ecosystem functioning and the provisioning of ecosystem services. This last part is touched on here and there and generally is not very well organized. Given the broad audience for this journal, it is reasonable to address these broader themes.

> *The format we use is common in recent ecological Nature articles (e.g. Brown et al. 2023; Fredston et al. 2023; Brodie et al. 2023) and needed to adhere to the tight word limit (2,500 words). Our results build on each step (i.e. from structure to robustness to a manipulative experiment to emergent properties and a null model). Therefore, we feel that to discuss each result without the others would confuse the main message and push us over the word limit by causing us to repeat conclusions that apply to multiple results. For instance, the structural differences in interaction evenness and the interaction complementarity results following from the strawberry experiment are the properties analysed in the null model. All of these results work together to support our conclusion that multiple contiguous habitats in the landscape support communities that are more even, more consistently robust, have better pollination function and that this cannot be explained by just summing up the habitat components.*
>
> *We strive to be honest about the caveats to our results throughout the manuscript (e.g., the potential effects of sampling completeness differences that we control for in the interaction complementarity analyses), conduct additional analyses to ensure our conclusions are not confounded by other effects (e.g., plant phylogenetic diversity in the null model), and run multiple scenarios to ensure we are capturing true effects (e.g., multiple robustness multiples with different extinction thresholds, dietary flexibility and removal scenarios). We use the whole manuscript to build to the conclusion paragraph starting in Line X and adding in a dedicated caveats section would lengthen the paper beyond the allowable limits. If the editor is amenable to extending the word limit for this purpose, however, we would of course be happy to add it to the manuscript.*

Specific comments, suggestions (Lines):

45, delete apostrophe in “species’ “

The apostrophe indicates this as a plural possessive: we are referring to the interactions of many species.

73-75, suggest “habitats affects one ecological function—pollination of a plant by insects. Finally, we use a modelling approach to investigate if landscape-scale networks have emergent properties that cannot be explained simply by summing their component habitat networks.

We have edited the wording accordingly and for clarity (Lines 71-72).

84, this would be a good place to make some of the points from the Methods and as brought up by reviewers about the experimental design and selection of sites

We present the experimental design and site selection in detail in the methods. The format of this journal does not enable us to go into the detail of the methods outside the main methods section (though again, we would be happy to add this should the editor be amenable); that said we do provide an overview of the methods in the main text.

90-94 Example here of a good place to explain the interpretation of these results. Then you can use the narrative lower down to talk about the meaning and relevance of this finding

As we discuss above, if we alter the manuscript to discuss each result independently it will considerably increase the word count.

94 “increases” … of what?

We have edited the wording for clarity (Line 95).

150 change to “differences”

Dispersion is the metric that is calculated. Our analysis plotted the pairwise distance among all pollinators’ diet in multi-dimensional space and then calculated the spread of all species from the origin (i.e., dispersion). Therefore, dispersion is the correct term in this instance.

156-57, suggest “thus sites with a pollinator community with more dispersed diets is expected to have higher interaction complementarity”

It is not the diets that are more dispersed, but rather communities that host species with more dissimilar diets will have higher dispersion when projected in partner space. This is the equivalent of having higher interaction complementarity.

160, suggest “... , they hosted sets …”

The manuscript reads more clearly if the verb tenses are consistent, so we have not changed this.

191, this would be a good place, again, to clarify what this finding means

As discussed above, since the interaction complementarity finding links with the emergent properties and null model analyses, we prefer to discuss this all together to avoid the repetition (it is also necessary, in order to remain within the word limit)

I would suggest adding a "Conclusion" section at L193 as described above

If the editor is amenable to expanding the word limit, we could both expand on our conclusions and include more of the caveats too.

REFERENCES

Bakdash, J. Z. & Marusich, L. R. Repeated Measures Correlation. *Frontiers in Psychology* **8**, (2017).

Banašek-Richter C, Cattin MF, Bersier LF. Sampling effects and the robustness of quantitative and qualitative food-web descriptors. Journal of Theoretical Biology. 2004 Jan 7;226(1):23-32.

Brodie, J.F., Mohd-Azlan, J., Chen, C. *et al.* Landscape-scale benefits of protected areas for tropical biodiversity. *Nature* **620**, 807–812 (2023). https://doi.org/10.1038/s41586-023-06410-z

Brown, P.T., Hanley, H., Mahesh, A. *et al.* Climate warming increases extreme daily wildfire growth risk in California. *Nature* (2023). https://doi.org/10.1038/s41586-023-06444-3

Fredston, A.L., Cheung, W.W.L., Frölicher, T.L. *et al.* Marine heatwaves are not a dominant driver of change in demersal fishes. *Nature* **621**, 324–329 (2023). https://doi.org/10.1038/s41586-023-06449-y

Henriksen MV, Chapple DG, Chown SL, McGeoch MA. The effect of network size and sampling completeness in depauperate networks. Journal of Animal Ecology. 2019 Feb;88(2):211-22.

Hendrickx F, Maelfait, JP, Van Wingerden W, Schweiger O, Speelmans M, Aviron S, Augenstein I, Billeter R, Bailey D, Bukacek R, Burel F. How landscape structure, land-use intensity and habitat diversity affect components of total arthropod diversity in agricultural landscapes. Journal of Applied Ecology. 2007 Apr;44(2):340-51.

Ives AR, Carpenter SR. Stability and diversity of ecosystems. Science. 2007 6;317(5834):58-62.

Kerr JT, Southwood TR, Cihlar J. Remotely sensed habitat diversity predicts butterfly species richness and community similarity in Canada. Proceedings of the National Academy of Sciences. 2001 Sep 25;98(20):11365-70.

Pacheco R, Vasconcelos HL. Habitat diversity enhances ant diversity in a naturally heterogeneous Brazilian landscape. Biodiversity and Conservation. 2012 Mar;21:797-809.

Rivera-Hutinel A, Bustamante RO, Marín VH, Medel R. Effects of sampling completeness on the structure of plant–pollinator networks. Ecology. 2012 Jul;93(7):1593-603.

Tscharntke T, Tylianakis JM, Rand TA, Didham RK, Fahrig L, Batáry P, Bengtsson J, Clough Y, Crist TO, Dormann CF, Ewers RM. Landscape moderation of biodiversity patterns and processes-eight hypotheses. Biological reviews. 2012 Aug;87(3):661-85.

Vizentin-Bugoni J, Maruyama PK, Debastiani VJ, Duarte LD, Dalsgaard B, Sazima M. Influences of sampling effort on detected patterns and structuring processes of a Neotropical plant–hummingbird network. Journal of Animal Ecology. 2016 Jan;85(1):262-72.

Reviewer Reports on the Second Revision:

Referees' comments:

Referee #2 (Remarks to the Author):

Major comment:

1.- The newly created figures 1, 2, and 3 of the rebuttal should be part of the extended data or the Supporting Information.

2.- Regarding completeness, it makes sense from Vizentin-Bugoni's paper to use weighted metrics as the authors say. However, the authors do not show how robustness and interaction evenness scale with completeness in the current dataset. Have those two metrics saturated at the level of completeness that the dataset provides? Are converging at different speeds for different sites, meaning that the results could potentially change if the sites were to be sampled more (or less)?

Looking at Figure 3 in the rebuttal, which the authors kindly created at my request, it is clear that the effect of size is inconsistent across habitats. However, it seems that the lack of replication at different habitat sizes prevents us from further investigating the effect of patch size for each habitat type individually.

These two potential problems with the current data make me think that factors like habitat size or sample completeness might be having an effect that cannot be quantified. The results in this paper, while correct for the current data, without being able to account for all these factors, might not hold true if the same sampling were done elsewhere. In the absence of a broader theoretical framework, this paper just represents a case study.

While I see value in the data, I don't find that the results are particularly supported beyond the in place A, under sampling effort B. Maybe it is a matter of how the study is currently presented.

As I wrote to the editor during my first assessment, I feel that this paper is not suggesting a new first principle, testing a first principle, discovering something new, or providing underwhelming evidence that either confirms or challenges existing knowledge. Those are the kind of things that I would expect in Nature.

Referee #3 (Remarks to the Author):

Thank you for this revision.

Given the complexity of the design, I applaud you for working hard, and with transparency as provided in the supplemental materials, to analyze these data. I don't have anything else to add on the methodologies and robustness of the study. I think this paper will be a valuable contribution to the field of landscape ecology and how biodiversity patterns respond to heterogeneity at different levels. This is an expansive study, well designed and well analyzed and I think it will be highly cited. I complement you, again, on this important work.

Minor points:

I actually think that some of the responses to Rev. 2's comments helped in explaining some of the interesting conclusions of the paper, especially as they related to questions of the value of landscape heterogeneity. I think that the questions raised about whether fragmentation is "good" forced the authors to better explain the ramifications of their findings. However, the response on L193 is a bit confusing as written: it states that preventing landscape fragmentation is a goal, *yet* … we need multi-habitat landscapes … Their response was better said in the rebuttal document to Rev. 2 which stated that we need to maintain *high-quality natural habitats in a diverse landscape* to get the features observed in this study. In short, I think invoking "fragmentation" is unnecessary in this sentence.

I still think that adding a short *Conclusion* section would benefit this paper. It would allow you to talk about the strengths and potential weaknesses of the study, and the ramifications for management and conservation. I will leave this to the editor to decide if it is worth the effort and extra length.

Author Rebuttals to Second Revision:

Referees' comments:

Referee #2 (Remarks to the Author):

Major comment:

1.- The newly created figures 1, 2, and 3 of the rebuttal should be part of the extended data or the Supporting Information.

We have added these figures to the Supporting Information. Figure 1 had already been added as SI Figure 4.1 and we have now added Supporting Information 5 to address the potential effects of sampling completeness, thereby providing the reader with clear evidence that sampling completeness is not confounding our results.

2.- Regarding completeness, it makes sense from Vizentin-Bugoni's paper to use weighted metrics as the authors say. However, the authors do not show how robustness and interaction evenness scale with completeness in the current dataset. Have those two metrics saturated at the level of completeness that the dataset provides? Are converging at different speeds for different sites, meaning that the results could potentially change if the sites were to be sampled more (or less)?

In the previous round of reviewing, we provided the proof that neither robustness, nor interaction evenness, were correlated with sampling completeness across landscape types (monad, dyad or triad) i.e. in relation to our experimental treatment. This is now included in Supplementary Information 5. Moreover, as Referee 2 points out, the weighted metrics that we use minimise any influence of sampling completeness. Nonetheless, given how concerned Referee 2 appears to be about this issue, we have performed a further site-level analysis for evenness (see below) which demonstrates that our conclusions are robust: even though there is some variation in the relationship between evenness and sampling completeness among sites, our conclusions hold across varying levels of completeness. In short, we feel that we have exhausted this issue and provided extensive reassurance that sampling completeness does not confound our conclusions.

Site level analysis of evenness and robustness

To further confirm a lack of confounding effect of sampling completeness on interaction evenness at each site in relation to sampling completeness. We simulated interaction accumulation using the iNext package (20 replicates for each of the 24 sampling completeness values tested at each site). Although rarefying the networks might reveal some relationship between increasing sampling completeness and interaction evenness (ranging from no effect to larger one when networks are particularly species poor, and with an inconsistent direction of

effect), restricting measurements to an equal level of completeness (e.g. 75%) would not affect our conclusions (Figure 1 below). This is consistent with our response in the previous round that differences in sampling completeness are not confounding our results. These plots and explanation are now included in Supplementary Information 5.

Undertaking the equivalent calculation for robustness, with 20 replicates for each of the 24 sampling completeness values tested at each site, for each of the 100 robustness simulations for all 30 sites, under 12 scenarios for both orders of removal (34,560,000 simulations in total!) is neither logical nor relevant for our results on robustness variability. Moreover, we have already clearly demonstrated that there is no discernible trend overall (i.e. it is a redundant analysis), see Supplementary information figure 5.2.

All of this is in line with of our previous analyses into potential implications of sampling completeness on our results, where we have consistently provided evidence that sampling completeness is not confounding our results nor affecting our conclusions. The potential for sampling completeness effects on interaction complementarity is already addressed in Supplementary Information 2. The null models were specifically used to account for any differences by selecting monad interactions at a sampling intensity equivalent to those recorded in triads. And we now include a Supporting Information Section to conclusively demonstrate that it is not confounding our structural nor robustness results. Note that the pollination experiment did not involve insect sampling and is thus unaffected by any sampling completeness differences.

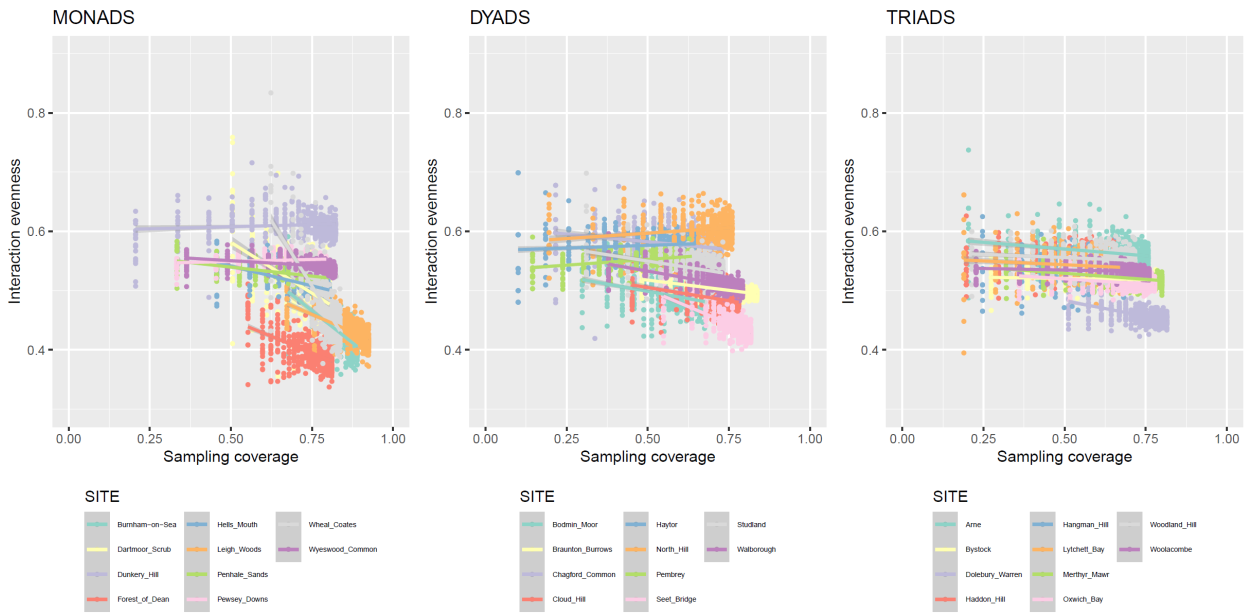

Figure 1. *Interaction accumulation curves with interaction evenness calculations plotted against sampling completeness for each monad (left), dyad (middle) and triad (right) site. For each site, networks were subsampled (20 times) to 24 values of sampling completeness below the maximum attained when preserving sampling effort. Although rarefying the networks may show some relationship between increasing sampling coverage and interaction evenness, it is not consistent and if sampling completeness were calculated at a consistent level (e.g. 75%), the conclusions would not change.*

3.- Looking at Figure 3 in the rebuttal, which the authors kindly created at my request, it is clear that the effect of size is inconsistent across habitats. However, it seems that the lack of replication at different habitat sizes prevents us from further investigating the effect of patch size for each habitat type individually.

> *The underlying rationale for selecting our field sites from a pool of different habitat types was to prevent a habitat effect occurring in the first place. It is this sampling design that Referees 1 and 3 praise so highly and in previous responses, we clearly demonstrate that there is no effect of patch size on any of the six structural measures (plant species richness, floral abundance, insect abundance, insect species richness and interaction evenness). The plots (Figure 2 copied below and in Supplementary Information 4) show no signal of a confounding effect. Referee 2 has already acknowledged that there is no consistent effect of patch size across habitats, which leaves us puzzled as to why this line of reasoning is continuing. In particular, each habitat type contributes to a limited number of total sites so that even if there were differences in the effect of patch size among habitats, in practice it would only contribute noise to the data set, rather than bias – in that sense our design is robust to putative patch size effects, whilst offering a good level of replication for detecting monad/dyad/triad effects.*

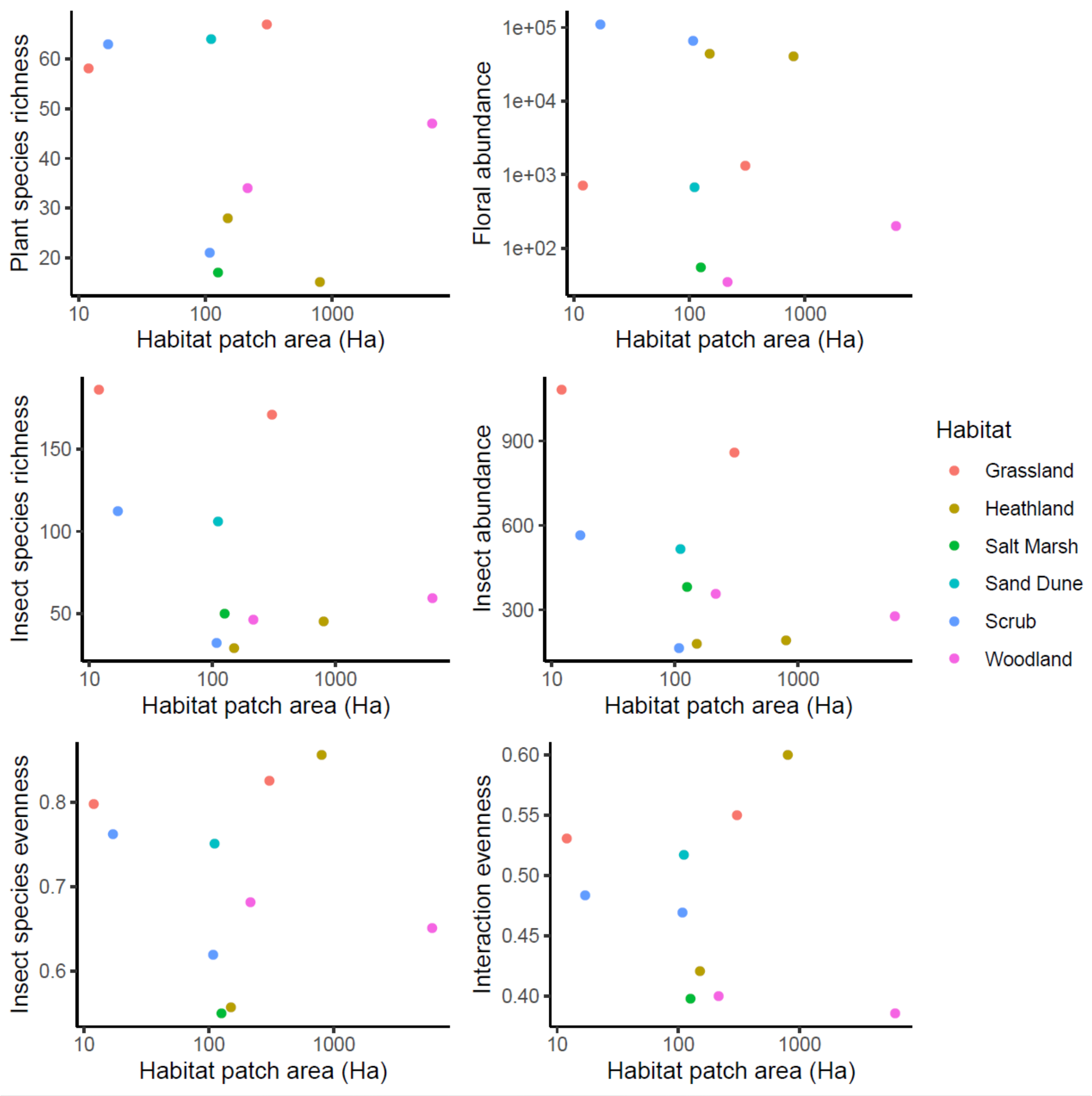

Figure 2: Surrounding habitat patch area of all monad sites plotted against the six structural measures (plant species richness, floral abundance, insect species richness, insect abundance, insect species richness, and interaction evenness) from our MANOVA. The habitat (grassland, heathland, salt marsh, sand dune, scrub and woodland) is indicated by the colour of the point. Accounting for habitat means that the power of any correlation analysis would be weak, but there is no discernible effect of patch size on any metric.

These two potential problems with the current data make me think that factors like habitat size or sample completeness might be having an effect that cannot be quantified. The results in this paper, while correct for the current data, without being able to account for all these factors, might not hold true if the same sampling were done elsewhere. In the absence of a broader theoretical framework, this paper just represents a case study.

As demonstrated above, neither potential problem is actually confounding our results. Our sampling was carried out at 30 independent field sites located across SW England and Wales, and results from these data are validated firstly by a manipulative field experiment which ran at 20 of these field sites, and secondly by a sophisticated set of null models. We also use a comprehensive robustness analysis, uniquely calculated across interaction types, accounting for shared species in multiple webs and investigating different levels of dietary flexibility and extinction thresholds. All of which support our conclusions on the benefits of multiple habitats in the landscape. We are concerned about what "effect that cannot be quantified" actually means in a scientific context? The most parsimonious explanation for an effect that cannot be quantified, is that there is no effect.

While I see value in the data, I don't find that the results are particularly supported beyond the in place A, under sampling effort B. Maybe it is a matter of how the study is currently presented. As I wrote to the editor during my first assessment, I feel that this paper is not suggesting a new first principle, testing a first principle, discovering something new, or providing underwhelming evidence that either confirms or challenges existing knowledge. Those are the kind of things that I would expect in Nature.

Surely all empirical field studies occur in place A under sampling protocol B? In our case, place A is 30 independent field sites and sampling effort B consists of sampling plants, three guilds of herbivores, parasitoids and pollinators (11,482 interactions among species) and considering data on six measures of community structure, complex modelling, and with a field experiment measuring pollination quantity and quality.

As highlighted by Referees 1 and 3, our sophisticated experimental design along with our robustness analyses, field experiment, interaction complementarity analysis and null models are a major step forward, and our results are consistent and convincing. Our work is novel because it provides a mechanistic understanding (i.e. a "broad theoretical framework") of how the number of habitats in a landscape contributes to community structure and function. Understanding this is key to the landscape-scale management of ecosystem services that depend on species interactions, such as pollination and pest control, and to maintaining functioning ecosystems more generally.

Our results will be of considerable interest to a wide audience. For the academic, there is a new conceptual framework as to how emergent properties such as robustness, interaction evenness and interaction complementarity are affected by the number of habitats while providing a mechanistic link from landscape to community structure to community function. For the conservation practitioner, our data show that adjacent habitats can significantly improve the stability and function of their reserve and provide a continuity of resources (e.g. nectar and pollen) that cannot be provided by their reserve alone. And for the rest of humanity, our data provides clear evidence that multiple habitats can support stability and provide better ecosystem service; information of considerable interest when the world is facing a biodiversity crisis.

Referee #3 (Remarks to the Author):

I actually think that some of the responses to Rev. 2's comments helped in explaining some of the interesting conclusions of the paper, especially as they related to questions of the value of landscape heterogeneity. I think that the questions raised about whether fragmentation is "good" forced the authors to better explain the ramifications of their findings. However, the response on L193 is a bit confusing as written: it states that preventing landscape fragmentation is a goal, *yet* … we need multi-habitat landscapes … Their response was better said in the rebuttal document to Rev. 2 which stated that we need to maintain *high-quality natural habitats in a diverse landscape* to get the features observed in this study. In short, I think invoking "fragmentation" is unnecessary in this sentence.

We have removed the direct reference to fragmentation in this sentence and edited it as suggested. We have also edited the text from the original version to ensure that we are not putting our results in contrast to effects of habitat fragmentation or preserving large natural habitat patches, but rather an important additional, but potentially overlooked, consideration. (Lines 190-199).

I still think that adding a short *Conclusion* section would benefit this paper. It would allow you to talk about the strengths and potential weaknesses of the study, and the ramifications for management and conservation. I will leave this to the editor to decide if it is worth the effort and extra length.

We are, of course, happy to add a Conclusion section if the editor requests it and allows the extra length.

Reviewer Reports on the Third Revision:

Referees' comments:

Referee #2 (Remarks to the Author):

All good. I think the authors have strengthened their manuscript dramatically during the review process. My most sincere congratulations.
